# Genome-Wide Identification, Structure Characterization, Expression Pattern Profiling, and Substrate Specificity of the Metal Tolerance Protein Family in *Canavalia rosea* (Sw.) DC

**DOI:** 10.3390/plants10071340

**Published:** 2021-06-30

**Authors:** Tao Zou, Ruoyi Lin, Lin Pu, Qiming Mei, Zhengfeng Wang, Shuguang Jian, Mei Zhang

**Affiliations:** 1 Key Laboratory of South China Agricultural Plant Molecular Analysis and Genetic Improvement & Guangdong Provincial Key Laboratory of Applied Botany, South China Botanical Garden, Chinese Academy of Sciences, Guangzhou 510650, China; zoutao@scbg.ac.cn (T.Z.); linry@scbg.ac.cn (R.L.); pulin@scbg.ac.cn (L.P.); qmei4597@scbg.ac.cn (Q.M.); wzf@scbg.ac.cn (Z.W.); jiansg@scbg.ac.cn (S.J.); 2 Center of Economic Botany, Core Botanical Gardens, Chinese Academy of Sciences, Guangzhou 510650, China; 3 University of the Chinese Academy of Sciences, Beijing 100039, China; 4 CAS Engineering Laboratory for Vegetation Ecosystem Restoration on Islands and Coastal Zones, South China Botanical Garden, Chinese Academy of Sciences, Guangzhou 510650, China; 5 Center for Plant Ecology, Core Botanical Gardens, Key Laboratory of Vegetation Restoration and Management of Degraded Ecosystems, Chinese Academy of Sciences, Guangzhou 510650, China; 6 Southern Marine Science and Engineering Guangdong Laboratory (Guangzhou), Guangzhou 511458, China

**Keywords:** metal tolerance protein, metal specificity, ecological adaptation, *Canavalia rosea* (Sw.) DC

## Abstract

Plant metal tolerance proteins (MTPs) play key roles in heavy metal absorption and homeostasis in plants. By using genome-wide and phylogenetic approaches, the origin and diversification of MTPs from *Canavalia rosea* (Sw.) DC. was explored. *Canavalia rosea* (bay bean) is an extremophile halophyte with strong adaptability to seawater and drought and thereby shows specific metal tolerance with a potential phytoremediation ability. However, *MTP* genes in leguminous plants remain poorly understood. In our study, a total of 12 *MTP* genes were identified in *C. rosea*. Multiple sequence alignments showed that all CrMTP proteins possessed the conserved transmembrane domains (TM1 to TM6) and could be classified into three subfamilies: Zn-CDFs (five members), Fe/Zn-CDFs (five members), and Mn-CDFs (two members). Promoter *cis*-acting element analyses revealed that a distinct number and composition of heavy metal regulated elements and other stress-responsive elements existed in different promoter regions of CrMTPs. Analysis of transcriptome data revealed organ-specific expression of CrMTP genes and the involvement of this family in heavy metal stress responses and adaptation of *C. rosea* to extreme coral reef environments. Furthermore, the metal-specific activity of several functionally unknown CrMTPs was investigated in yeast. These results will contribute to uncovering the potential functions and molecular mechanisms of heavy metal absorption, translocation, and accumulation in *C. rosea* plants.

## 1. Introduction

Heavy metals (HMs) play essential roles as the necessary cofactors of some metabolism-related enzymes, transcription factors, and signal transduction pathways at low levels. In contrast, when they are present at high concentrations, these HMs can cause harmful effects on plant growth and cellular metabolism. Essential HMs include copper (Cu), zinc (Zn), manganese (Mn), nickel (Ni), and cobalt (Co). Other nonessential HMs, such as cadmium (Cd), mercury (Hg), silver (Ag), and lead (Pb), even at an extremely low level, are highly toxic to plants [1]. Basically, plants need essential HMs and acquire them from the environment, including soil, water, and air, but because their absorption process is not perfect, plants may also take up nonessential HMs or over-absorb essential HMs [2]. The toxic effects of nonessential HMs or excess essential HMs to plants include causing oxidative damage and metabolic disorders, resulting in chlorosis, necrosis, and growth inhibition [3].

Plants have developed a complex network of metal uptake, efflux, chelation, trafficking, and storage mechanisms to ensure precise metal homeostasis. Cation diffusion facilitator transporters (CDF, TC 2. A.4) are designated as metal tolerance proteins (MTPs) in plants and have been identified as one of the most important HM transporter families [4,5]. The CDF transporters were first identified by Nies and Silver [6], and numerous studies have indicated that this family exists widely in all kingdoms of life and can be classified into three subfamilies: Zn-CDF, Fe/Zn-CDF, and Mn-CDF, mainly based on their substrate specificity [5]. Plant MTPs were further classified into seven groups according to their amino acid sequence homologies [7]. Corresponding to the former classification, the Zn-CDF subfamily comprises groups 1, 5, and 12; the Fe/Zn-CDF subfamily comprises groups 6 and 7, and the Mn-CDF subfamily comprises groups 8 and 9. In terms of structural features, most of the MTPs contain four to six transmembrane domains (TMDs) with cytoplasmic N and C termini, a modified signature, and a C-terminal cation efflux domain (PF01545) [5]. 

Until now, many studies have revealed that plant MTPs play vital roles in the absorption and detoxification of HMs, promoting their accumulation in plants [4]. The first plant *MTP* member was designated *ZAT* (Zn transporter of *Arabidopsis thaliana*), which is an Arabidopsis orthologue of animal *ZnT* genes (Zn transporters) [8]. *ZAT* was later renamed *AtMTP1*, and its mutation in Arabidopsis resulted in plant sensitivity to Zn, while overexpression of *AtMTP1* showed a tolerant phenotype to Zn in transgenic Arabidopsis [9]. As an important divalent metal transporter, the substrate and metal specificity of different MTP members generated interest in both nutrient metal uptake and phytoremediation of HM contamination [10,11,12,13,14,15]. Arabidopsis contains 12 *MTP* genes. In addition to AtMTP1, AtMTP3 has been proven to be involved in Zn and cobalt (Co) tolerance [16], and AtMTP11 is a manganese (Mn) transporter [17,18]. AtMTP12 can interact with AtMTP5t1 and transport Zn into the Golgi [19]. AtMTP8 transports both Mn and iron (Fe) [20,21,22]. Rice is a cadmium (Cd)-concentrating crop, and OsMTP1 shows a certain Cd-transporting ability, mediates Cd absorption in plants, and transports Zn and Ni [3,23]. *OsMTP1* overexpression in tobacco also showed enhanced Cd accumulation and tolerance, implying that it could be used in phytoremediation [12]. Both OsMTP8 and OsMTP11 can transport Mn across a transmembrane and are involved in Mn tolerance in rice [24,25,26,27,28,29,30]. In addition, nonhyperaccumulator cucumber *MTP* members showed Zn transporting features and regulatable subcellular localization patterns [31]. 

Notably, in some metal hyperaccumulators, *MTP* members seem to contribute more greatly to metal tolerance and enrichment in plants. *Thlaspi goesingense* can hyperaccumulate Ni/Zn in vivo, and *TgMTP1* shows a high level of expression in *T*. *goesingense* and functions to enhance Ni, Zn, Co, and Cd tolerance when overexpressed in yeast [32,33]. In transgenic Arabidopsis, *TgMTP1* is likely to act in transporting Zn into the vacuole, therefore enhancing both Zn accumulation and tolerance [34]. *Arabidopsis halleri* is a natural Zn hyperaccumulator. AhMTP1 can transport Zn, and compared to its homolog *AtMTP1* in Arabidopsis, *AhMTP1* shows a much higher expression level in *A. halleri* than *AtMTP1* in Arabidopsis [35]. Further research showed that gene expansion has occurred in the *AhMTP1* locus in the *A. halleri* genome [36]. Furthermore, other plant *MTPs* isolated from metal hyperaccumulators, such as *ShMTP1* from *Stylosanthes hamata* (Mn tolerant) [37], *PtdMTP1* from hybrid poplar (Zn/Ni tolerant) [10], *SaMTP1* from *Sedum alfredii* Hance (Zn tolerant) [38], and *BjCET2/3/4* from *Brassica juncea* (Zn/Cd tolerant) [39,40], showed typical HM transporting or accumulation features. 

As a mangrove-associated and halophyte species distributed widely in tropical and subtropical seashore and arid coral reefs, *Canavalia rosea* (Sw.) DC. exhibits tenacious vitality under high salinity, extreme drought, and even some HM-polluted coastal areas. It has been proposed that halophytes can also enrich HMs and that their ability to tolerate HM stress relies on similar physiological and molecular mechanisms to those exhibited under high saline–alkaline stress [41]. Halophyte can cope with several abiotic constraints occurring simultaneously in their natural environment by sequestering absorbed toxic ions (including salty ions and HMs) in trichomes or vacuoles, as well as maintaining regular biomass production and relatively stable plant water status for plant growth. These characteristics cause some halophytes to be promising candidates for phytoremediation management of heavy-metal-polluted areas in saline environments [42]. Some saline shrubs or mangrove species have been proven to possess the abilities to absorb and accumulate salt and toxic metals from saline soils efficiently and therefore can be used as potential candidate engineering plants for the restoration of saline soils contaminated with heavy metals [43,44,45,46,47]. Some reports have also proven that halophytes adopt similar physiological, biochemical, and molecular mechanisms to cope with the toxic levels of salinity and heavy metals [41,42,48]. In China and many other countries, coastal wetlands show severe HM pollution due to emissions from inland rivers [49]. Mangrove species can be used for ecological restoration and phytoremediation of vegetation in tropical and subtropical coral sand and coastal beaches because of their strong tolerance for salt and HM mixed pollution. Therefore, it is necessary to determine the molecular mechanisms of this special habitat’s halophyte, *C. rosea*, for adsorption and enrichment of HM from polluted water and soil. In addition to many genome sequences for plant species, characterization of gene families has also been concerned with gene expression and biochemical functional identification, especially from evolutionary biology. Based on our previous genomic sequence project on *C. rosea*, the *CrMTP* sequences were isolated from its genome data, and a total of 12 *CrMTP*s were selected for further research. In this study, *CrMTP*s’ evolution and structural features were systematically analyzed, as well as the promoters’ characteristics and gene expression patterns. RNA-seq experiments were also performed to identify the possible biological functions of this gene family responding to HMs or other abiotic stressors. Moreover, their potential metal substrates were also explored by a yeast heterologous expression assay with a series of mutant strains. This study provides a basis for the systematic understanding of the *C. rosea MTP* family involved in HM translocation, offering potential scientific evidence for halophytes applying for phytoremediation purposes, and further illuminates the roles of plant *MTP*s involved in adaptation to extreme adversity.

## 2. Results

### 2.1. An Overview of C. Rosea CrMTP Genes

**Figure 1:**
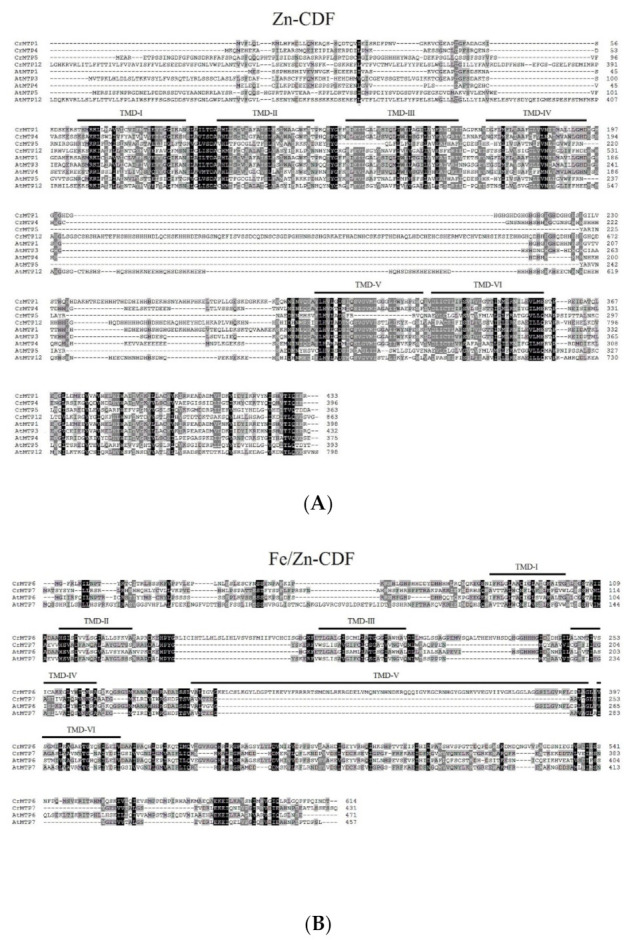

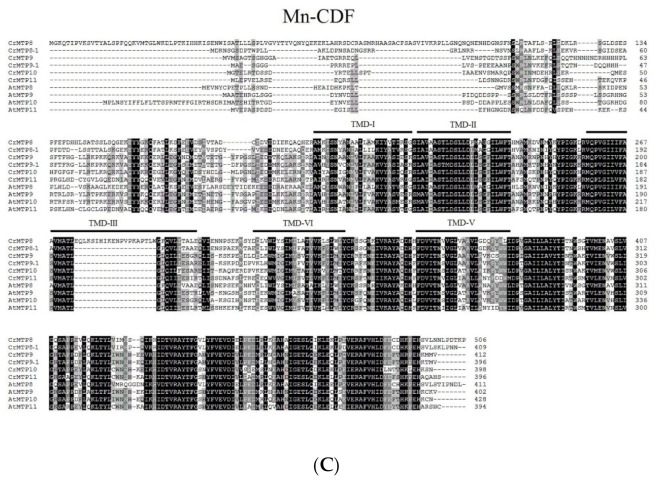
Multiple sequence alignment of CrMTP and AtMTP proteins. A total of 12 CrMTP proteins were identified in *Canavalia rosea* (Sw.) DC. and AtMTP proteins from *Arabidopsis thaliana* were classified into Zn-CDFs (**A**), Fe/Zn-CDFs (**B**), and Mn-CDFs (**C**) and then aligned using ClustalW. The transmembrane domains (TMDs I–VI) were marked with bold lines.

A total of 12 *CrMTP* genes were identified from the *C. rosea* genome. Bioinformatics analysis showed that all CrMTPs contained the Cation_efflux domain (PF01545), thereby suggesting that these possess a basic characteristic of the MTP family. Among them, seven CrMTPs possessed a ZT_dimer domain (PF16916) near their C-terminals, which often lie within the cytoplasm and form homodimers during protein activities. Based on their sequence similarity to Arabidopsis MTPs and the gene nomenclature system, the *C. rosea* MTPs were correspondingly designated CrMTP1 to CrMTP12 (Table 1). The length of the *CrMTP* coding DNA sequences ranged from 1032 bp (CrMTP1) to 2592 bp (CrMTP12) with 343–863 amino acid residues. Members of the *CrMTP* gene family were subdivided into classes Zn-CDF, Mn-CDF, and Zn/Fe-CDF according to their homologies with AtMTPs (Figure 1).

The physicochemical parameters, including the molecular weight (MW), theoretical isoelectric point (pI), and grand average of hydropathicity (GRAVY) values of the CrMTP proteins were predicted in Table 1. The MWs ranged from 40.758 kD (CrMTP5) to 97.802 kD (CrMTP12), and most of them were within 40–50 kD. Their theoretical pI values, between 5.08 and 7.77, showed that CrMTPs are basically neutral (Table 1). The GRAVY results, ranging from −0.137 (CrMTP10) to 0.146 (CrMTP4), indicated that the CrMTPs generally had weak hydrophilicity, which was consistent with the MTPs’ biochemical functions as transmembrane transporters (Table 1). Transmembrane helices (TMHs) predication using TMHMM and PHYRE^2^ was almost consistent with little differences (Figure S1A,B), and the topologies, including the embedded directions of transmembrane helices in CrMTPs, indicated the functional diversity of different CrMTP members diffusing the metals (Table 1 and Figure S1C).

Subcellular localization prediction for CrMTPs was performed with different programs (Table 1). The results indicated that CrMTPs were widely distributed in different endomembrane parts of organelles, as well as in cell plasma membranes. Generally speaking, as a kind of membrane protein, the presence of transmembrane domains in each CrMTP member provides necessary conditions for cation cross-membrane transportation (Figure S1), while the detailed subcellular localization status of specific CrMTP might be determined more by protein modification, protein interaction, or even metal binding, which was presumed as a dynamic and regulated process and also as a manner for metal distribution in vivo when under excess HM stress.

**Figure 2:**
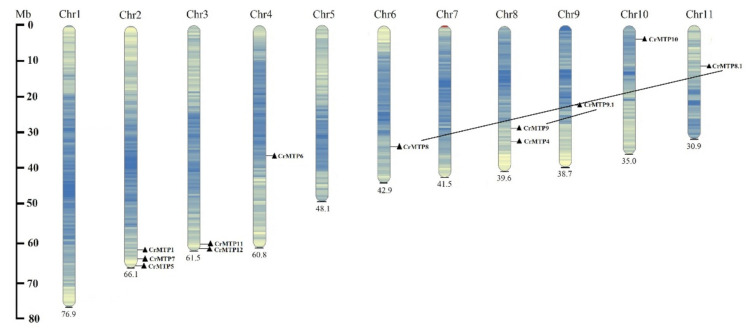
Chromosomal location and gene duplication of 12 CrMTP genes in the *C. rosea* genome.

The physical map of the *CrMTP* family showed an uneven distribution of *CrMTP* genes on the 11 chromosomes of *C. rosea—*the most genes on chromosome 2 (three genes), followed by chromosomes 3 and 8 with two genes each, whereas chromosomes 1, 5, and 7 carried no *CrMTP* genes (Figure 2). Synonymous (Ks) and nonsynonymous (Ka) values were calculated to explore the selective pressures on the duplication of *CrMTP*s based on all nucleotide sequences. The results revealed that two gene pairs, CrMTP8/CrMTP8.1 and CrMTP9/CrMTP9.1, possessed Ka/Ks ratios greater than 0.1 that were lower than 1, indicating that they underwent some purifying selection and demonstrated segmental duplications, which might suggest their evolution and functional divergence (Table 2).

### 2.2. Multiple Sequence Alignment and Phylogenetic Analysis of the CrMTP Family

CrMTP protein sequences were further aligned with AtMTPs according to a previous report (Figure 1) [14]. The results indicated that the MTPs were highly conserved in phylogeny even in different plant species. As shown in Figure 1, 12 CrMTP proteins were classified into three major subgroups, namely, Zn-CDF, Fe/Zn-CDF, and Mn-CDF. The transmembrane domains (TMDs) of the CrMTPs predicted by TMHMM that were critical for cation channel establishment are marked in Figure 1. These domains displayed higher conservation than other sequences, while the N-terminus of all CrMTPs showed great variation, which might be related to specific post-translational modifications, protein interactions, or subcellular localization. In the Zn-CDFs subgroup, most of the members in this subgroup had a variable histidine-rich (His-rich) loop between TMD-IV and TMD-V, which has been proven to be critical to metal selectivity or as sensors of the cellular metal concentration in plant MTPs [15,50]. The His-rich loops of Zn-CDFs from Arabidopsis and *C. rosea* varied greatly in length and histidine content (Figure 1A), further indicating the fine regulation mechanisms of MTPs caused by metal substrates. The Zn/Fe-CDF subgroup showed the greatest variability both in TMDs and other sequences, and more specifically, the CrMTP6 showed an extra sequence (KELCSLKGYLDGPTIKEVYFRRRRTSMDNLRKRGDELVMQNYNHNDKRQQQIGVKGCRNHGYGGNKVVEGVIIVGKLGGLAG) in the putative TMD-V (Figure 1B), which was quite different from other MTP6s [14] and might destroy the topological structure of CrMTP6 and affect the biological function for transporting metals. In addition, Mn-CrCDF subgroups, including CrMTP8, CrMTP8.1, CrMTP9, CrMTP9.1, CrMTP10, and CrMTP11, were more conserved than the other two subgroups (Figure 1C).

**Figure 3:**
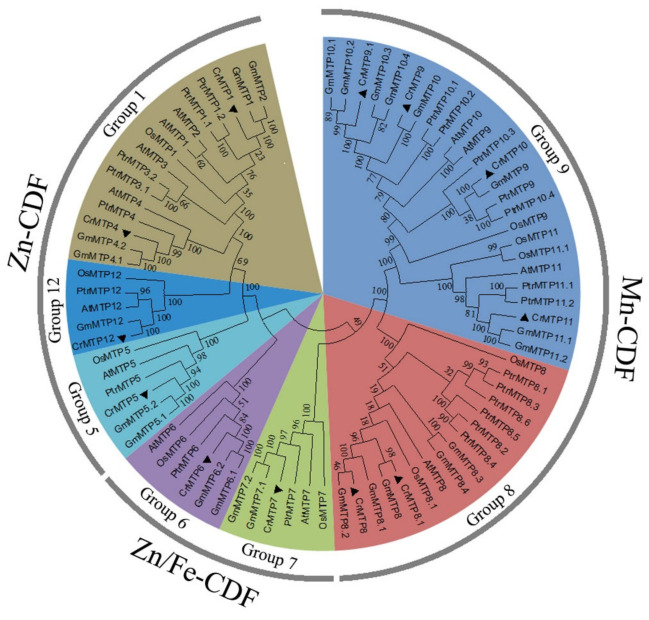
Phylogenetic tree of CrMTPs from *C. rosea*, Arabidopsis, soybean, rice, and poplar. The neighbor-joining (NJ) tree was constructed using Clustal W and MEGA 6.0 software with 1000 bootstrap replicates.

To study the evolutionary relationships between *C. rosea* MTP proteins and known MTPs from Arabidopsis, soybean, rice, and poplar, an unrooted neighbor-joining phylogenetic tree was created based on multiple alignments of the predicted sequences of the MTP proteins from these plants. There were no *AtMTP2* or *AtMTP3* orthologous genes found in the *C. rosea* genome, similar to those found in the soybean genome (Figure 3), while the Mn-CDF subgroup members, CrMTP8, CrMTP8.1, CrMTP9, and CrMTP9.1, seemed to have doubled compared with other plant species [14], which is in agreement with previous reports [51,52,53]. This indicates gene duplication events. Ka versus Ks substitutions (Ka/Ks) were estimated with nucleotide sequences of *CrMTP8/8.1* and *CrMTP9/9.1* gene pairs, which showed segmental duplication events within the *C. rosea* genome as paralogs (Table 2).

### 2.3. Gene Structure and Conserved Motifs of CrMTP Members

**Figure 4:**
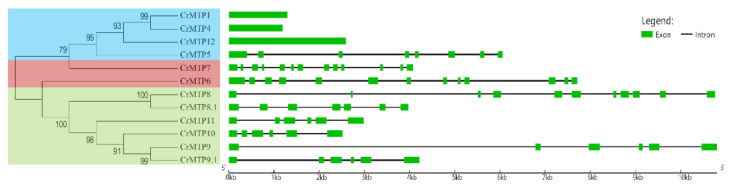
CrMTPs’ classification and gene structures of CrMTP genes in *C. rosea*. The left part shows that the CrMTPs phylogenetic tree is classified into three subgroups (Zn-CrCDFs, in blue; Fe/Zn-CrCDFs, in red; Mn-CrCDFs, in green). The left part shows the exon–intron gene structures of all *CrMTP*s.

The gene exon–intron organization for each *CrMTP* was examined to analyze the evolution of this gene family further. As shown in Figure 4, in the coding region sequences of *CrMTP* members, Zn-CDF members, CrMTP1, CrMTP4, and CrMTP12 were intron-less, while other *CrMTP* members contained 5–12 introns. *CrMTP* genes that clustered closely showed similar exon numbers and gene structures (Figure 4), which was consistent with the results of phylogenetic analysis and classification of MTP families in other species [14,51,52,54]. Notably, the gene structure of different *MTP* families also represented a certain degree of similarity and conservation in different plant species.

**Figure 5:**
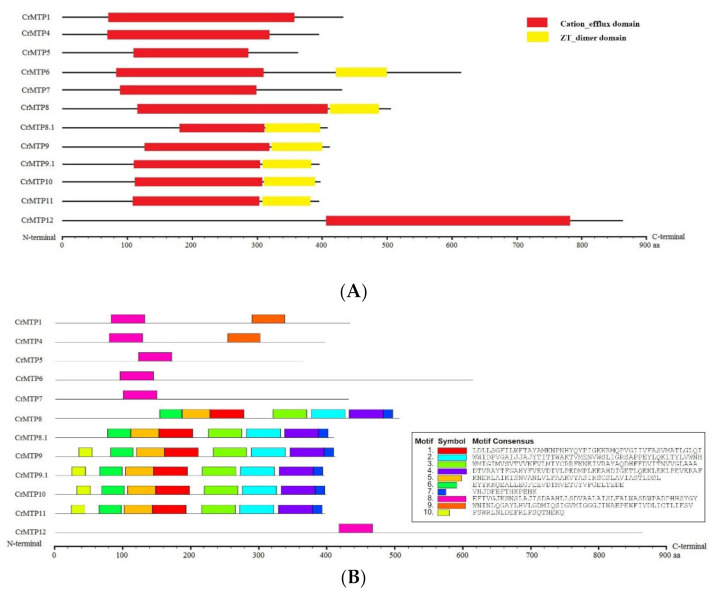
Conserved domains searched by the Pfam database and their conserved motifs discovered by Multiple Em for Motif Elicitation (MEME) motif discovery server of CrMTP proteins in *C. rosea*. (**A**) The protein families (PFAM) domain diagrams of CrMTPs, searched by PFAM database; (**B**) Conserved motifs of CrMTPs, identified using Multiple EM for Motif Elicitation (MEME) database. The websites of PFAM and MEME databases were listed in “Materials and Methods” part.

To understand the possible biochemical roles and substrate specificities of *C. rosea* MTP proteins, the conserved CDF domain (PF01545) and ZT_dimer domain (PF16916) were investigated (Figure 5A). Protein domain analyses supported that all CrMTP proteins possessed the typically conserved functional CDF domain, which contained signature sequences of CDF members (Figure 1) [5]. Half of the CrMTP members (CrMTP6 and five Mn-CDF-type CrMTPs) had the ZT dimer, which belongs to an important zinc transporter dimerization domain (Figure 5A). The conserved motifs search also indicated that the members of the same cluster or group in the CrMTP family had similar motif types and distributions (Figure 5B).

### 2.4. Heavy-Metal- and Abiotic-Stress-Related cis-Acting Elements in CrMTP Promoters

To obtain further insight into the putative biological function of *CrMTP* genes in *C. rosea*, *cis*-acting elements located in the promoter and untranslated regions (UTR) of each gene were investigated. The regulation of *CrMTP* expression remains a key mediator of biological functions, especially in response to HM stresses. In this study, putative *cis*-acting elements were identified in the promoter regions of all *CrMTP*s by scanning the CDS upstream 2000 bp sequences of *CrMTP* members with the online PlantCARE program.

**Figure 6:**
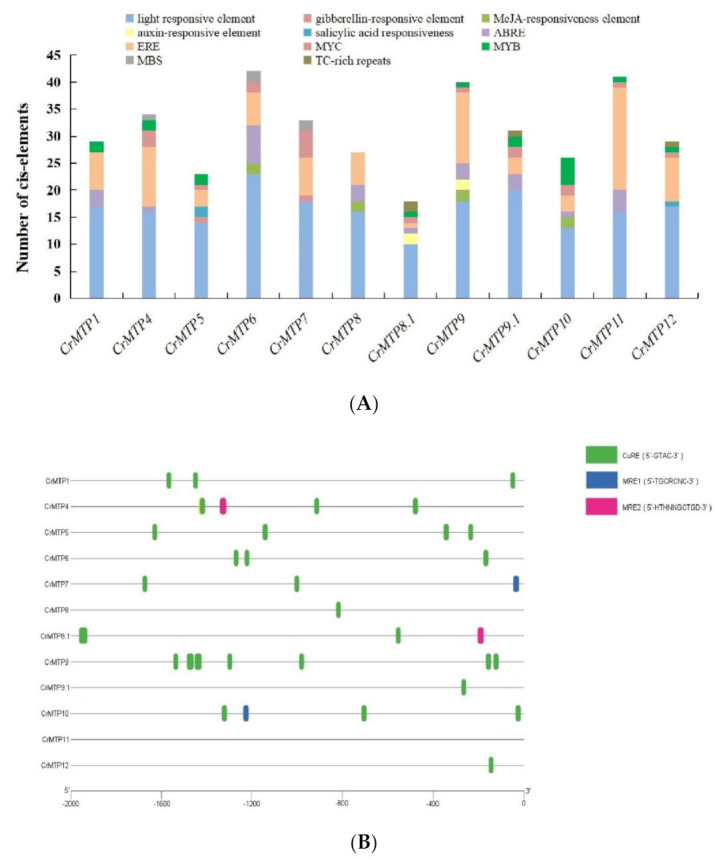
Predicted *cis*-acting elements in CrMTP promoter regions of *C. rosea*. The 2000 bp sequence upstream of 12 CrMTP genes’ coding region was analyzed by PlantCARE and manually searched with MRE1, MRE2, and CuRE: (**A**) different *cis*-acting elements were identified and plotted against a bar diagram. The abundance of different regulatory elements on each of the promoters is shown in different colors; (**B**) the location of metal stress responding *cis*-acting elements (MRE1, MRE2, and CuRE) in 12 CrMTP promoter regions.

The *cis*-acting regulatory elements in the *CrMTP* promoter regions searched online were simply classified into 11 categories: light-responsive elements, gibberellin-responsive elements, MeJA-responsiveness elements, auxin-responsive elements, salicylic acid-responsiveness elements, ABA-responsive elements (ABRE), ethylene-responsive elements (ERE), MYC-binding elements (MYC), MYB binding site involved in drought-inducibility (MBS), and TC-rich repeats (*cis*-acting element involved in defense and stress responsiveness). In summary, the numbers of these elements in each *CrMTP* promoter region were specific (Figure 6A), indicating that the expression of each *CrMTP* gene could be systematically regulated by a series of environmental or developmental factors. In addition, because *MTP*s play an important role in maintaining plant metal tolerance and homeostasis, three kinds of metal-responsive elements (MRE1, MRE2, and CuRE) were manually searched. These HM stress-related *cis*-acting elements were summarized within 12 *CrMTP* promoter regions (Figure 6B), and the results showed that CuRE was the most dominant and was present in almost all *CrMTP* promoter regions (except *CrMTP11*). MRE1 existed in *CrMTP7* and *CrMTP10* promoter regions, while MRE2 existed in *CrMTP4* and *CrMTP8.1* promoter regions. Further functional studies are still necessary to confirm the functions of these *cis*-acting elements in the regulation of *CrMTP*s’ expression patterns.

### 2.5. Expression Profiles of CrMTPs in Different Tissues or under Different Stress Treatments

**Figure 7:**
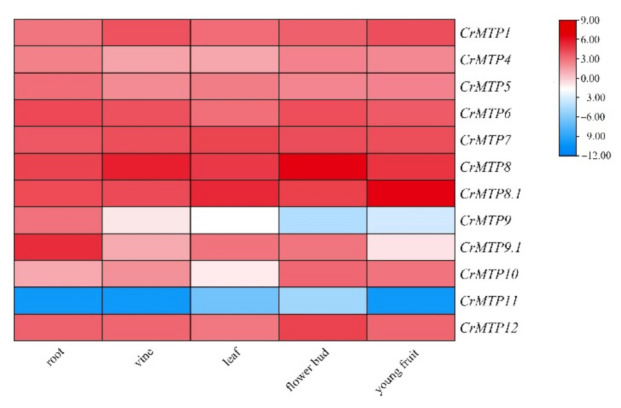
Heatmaps showing the expression patterns of *CrMTPs* in various tissues (root, vine, leaf, flower bud, and young fruit) of *C. rosea* plants. The expression level of each gene is shown with the values of FPKM (log2). Red denotes high expression levels, and blue denotes low expression levels.

To investigate whether the predicted *CrMTP* genes were actually transcribed, their transcription levels were examined in different tissues using RNA-seq. As shown in Figure 7, eight genes (*CrMTP1*, *4*, *5*, *6*, *7*, *8*, *8.1*, and *12*) were highly expressed in all five tissues. The expression levels of the other members (*CrMTP9*, *9.1*, *10*, and *11*) varied among these tissues.

**Figure 8:**
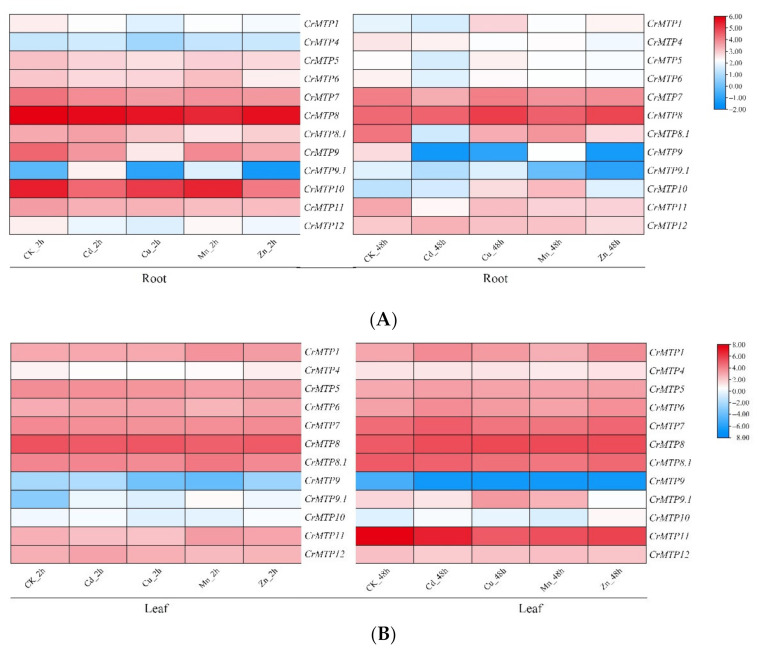
Heatmaps showing the expression patterns of *CrMTPs* under different HM challenges (0.1 mM CdCl_2_, 0.1 mM CuSO_4_, 1 mM MnCl_2_, and 0.5 mM ZnSO_4_) both in roots (**A**) and in leaves (**B**). The expression level of each gene is shown with the values of FPKM (log2). Red denotes high expression levels, and blue denotes low expression levels.

To explore the biological functions of *CrMTP* genes further, expression changes in the entire MTP family of *C. rosea* seedlings were analyzed in the presence of different metal ions, including Zn, Cd, Mn, and Cu. The *CrMTP*s showed various responses to the same metal ion either in the roots or leaves (Figure 8), and the expression of certain genes slightly changed under different metal treatments (Figure 8 and Figure S2). In root samples, except *CrMTP8* and *CrMTP10*, the other 10 *CrMTP*s showed slightly downregulated expression levels under different HM challenges, either at 2 or 48 h (Figure 8A). However, overall, the expression levels of *CrMTP* genes remained relatively stable in leaves, and only *CrMTP1* and *CrMTP9.1* showed slightly elevated expression changes under different HM challenges, both at 2 and 48 h (Figure 8B). When comparing two control samples (CK 2 h and CK 48 h) in root samples, *CrMTP8*, *CrMTP9*, and *CrMTP10* generally showed elevated expression levels in the CK 48 h sample, compared to the CK 2 h sample. In leaf samples, only *CrMTP11* showed greatly induced expression in the CK 48 h samples, compared to the CK 2 h samples. Considering that *C. rosea* is a mangrove-associated species, seawater logging is normal in their growing habitats; therefore, the obvious expression changes in specific *CrMTP* members are possibly an adaptive mechanism for the normal growth of *C. rosea* plants.

**Figure 9:**
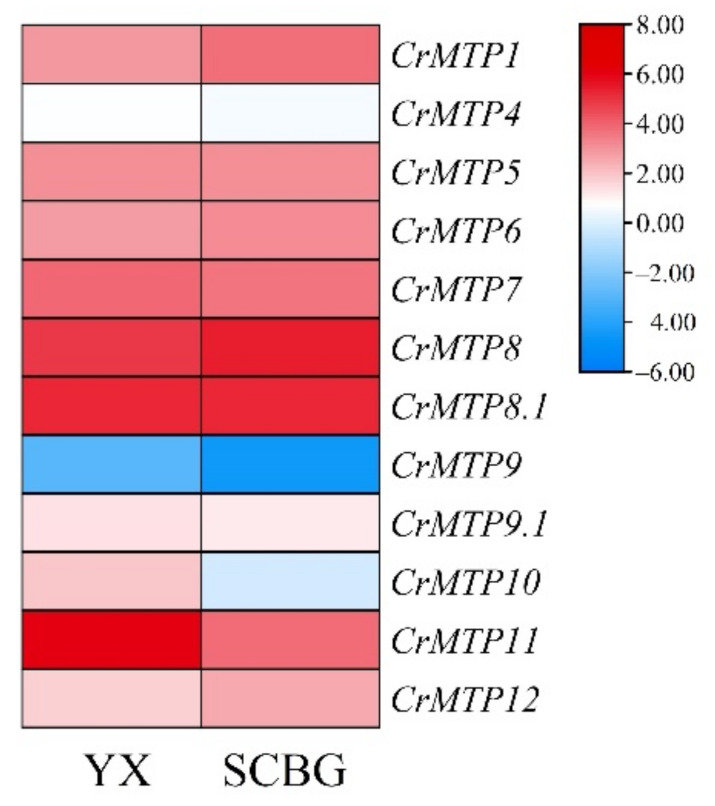
Heatmaps showing the expression differences of the *CrMTPs* in mature *C. rosea* leaves planted in South China Botanical Garden (SCBG) and in Yongxing Island (YX). The expression level of each gene is shown with the values of FPKM (log2). Red denotes high expression levels, and blue denotes low expression levels.

To further explore the possible roles of *CrMTP*s in *C. rosea*’s adaptation to special habitat with extreme adversity on tropical coral reefs, RNA-seq data for *CrMTP* members were also extracted from transcriptome sequencing for mature leaves captured from YX Island and SCBG (Figure 9). Only *CrMTP1* and *CrMTP12* showed slightly downregulated expression changes in the YX Island leaf sample, while in the SCBG sample, *CrMTP10* and *CrMTP11* showed greatly induced expression.

Four-week-old *C. rosea* seedling plants were subjected to high salinity and ABA treatments, and the expression changes were also detected by qRT-PCR assays. The relative expression levels of *CrMTP*s in response to salt stress and ABA in *C. rosea* roots, vines, and leaves varied to different degrees (Figure S3). Generally, high salinity decreased the expression levels of most *CrMTP* members, and ABA upregulated some *CrMTP* members’ expression in all three tested tissues of *C. rosea* seedlings (*CrMTP1*, *CrMTP4*, *CrMTP6*, and *CrMTP9*).

### 2.6. Functional Characterization of CrMTPs in Yeast

**Figure 10:**
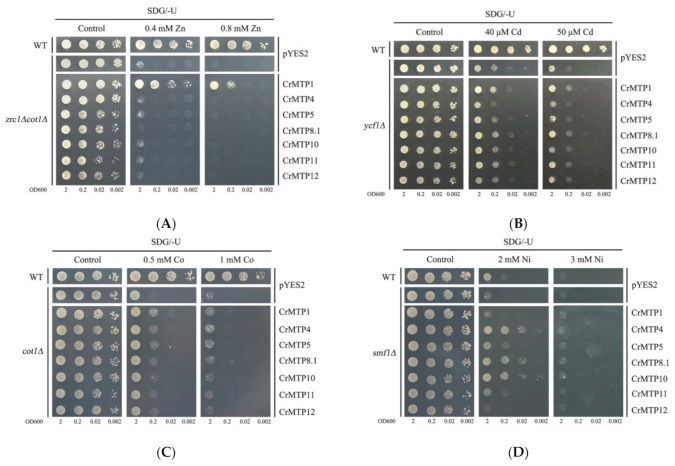

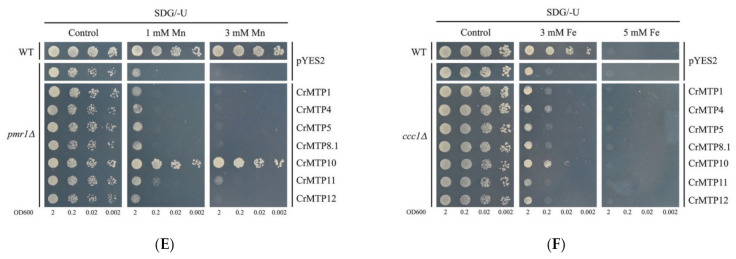
Complementation of yeast mutants on solid medium containing heavy metals. *S. cerevisiae* wild-type strain BY4741 was transformed with the empty vector pYES2, and mutant strains were transformed with the empty vector pYES2 or with the vectors carrying the *CrMTP* gene. Yeast cultures were adjusted to OD600 = 2, and 2 μL of serial dilutions (10-fold, from left to right in each panel) were spotted on SDG–Ura medium supplemented with 0.4 or 0.8 mM ZnSO_4_ (**A**), 40 or 50 μM CdCl_2_ (**B**), 0.5 or 1 mM CoCl_2_ (**C**), 2 or 3 mM NiCl_2_ (**D**), 1 or 3 mM MnCl_2_ (**E**), and 3 or 5 mM FeCl_2_ (**F**) on SDG–Ura plates. The corresponding yeast spots growing on SDG–Ura plates without heavy metals were used as controls. The plates were incubated for 2–5 days at 30 °C. The images are representative of three independent experiments.

For functional characterization of different *CrMTP* members, the complete open reading frame (ORF) of the seven *C. rosea* MTPs were PCR amplified. The yield recombinant vectors for CrMTPs’ heterologous expression in yeast (CrMTPs-pYES2) are shown in Figure S4. Interestingly, by using cDNA from *C. rosea* seedling roots or leaves as a template, five *CrMTP* members could not be amplified successfully. Specifically, CrMTP6 and CrMTP7 had relatively high expression in different *C. rosea* tissues (Figure 10). This was likely caused by primer specificity or more detailed expression patterns of these *CrMTP* members. Furthermore, the other seven CrMTP members amplified by RT-PCR showed identical sequences with bioinformatic analysis results.

After the ORFs were inserted into yeast expression vector pYES2, the role of CrMTPs in Zn^2+^, Cd^2+^, Co^2+^, Ni^2+^, Mn^2+^, and Fe^2+^ transport was determined by testing the complementation of the yeast mutants’ phenotype. The double mutant *zrc1∆cot1∆* was defective for both Zn^2+^ transporter *ZRC1* and Co^2+^ transporter *COT1* and highly sensitive to Zn [15]. As shown in Figure 10A, the expression of CrMTP1 could rescue the sensitivity of *zrc1∆cot1∆* to Zn, while other members could not. The complementary capability of CrMTPs to the Cd-sensitive phenotype of *zrc1∆cot1∆* was also tested; however, the effects were not visible (data not shown). *ycf1∆* was highly sensitive to Cd, and none of these seven CrMTP members could completely complement the Cd sensitivity phenotype of *ycf1∆* mutant, although CrMTP1, CrMTP8.1, and CrMTP11 seemed to slightly improve the Cd tolerance (Figure 10B). Similarly, CrMTP1, CrMTP4, and CrMTP5 slightly improved the Co tolerance of *cot1∆* (Figure 10C). At relatively lower Ni concentration (2 mM), CrMTP4, CrMTP8.1, and CrMTP10 significantly improved the Ni tolerance of *smf1∆*, while at higher Ni concentration (3 mM), the effects were not visible (Figure 10D). Although CrMTP8.1, CrMTP10, and CrMTP11 are all Mn-CDF members, only CrMTP10 obviously improved the Mn tolerance of *pmr1∆*, and CrMTP11 only showed a slight effect (Figure 10E). The only two Fe/Zn-CDF members CrMTP6 and CrMTP7 were not successfully cloned, while in the Fe sensitivity assay of yeast mutant *ccc1∆*, both CrMTP4 and CrMTP10 enhanced yeast growth on plates with lower Fe (3 mM) (Figure 10F). Overall, the ability to transport different bivalent cations of CrMTPs depends on both member specificity and concentration of cations.

## 3. Discussion

The cation diffusion facilitators (CDFs) family is a ubiquitous family of membrane-bound HM transporters involved in metal tolerance/resistance. Cation diffusion facilitators are found in all major phyla of living organisms, including Archaea, Eubacteria, and Eukaryotes [7,55]. Plant CDFs are commonly called metal tolerance proteins (MTPs). To date, the *MTP* gene family has been reported in a number of plant species, but information on this family is limited in leguminous plants. In this study, the *C. rosea* genome was comprehensively searched for *MTP* family analysis, which resulted in the identification of 12 putative *CrMTP* genes. 

Plant MTP family members belong to divalent cation (Zn^2+^, Co^2+^, Fe^2+^, Cd^2+^, Ni^2+,^ and Mn^2+^) transporters, which are mainly involved in metal ion efflux from the cytoplasm, either to the outside of the cell or into subcellular compartments [5]. In plants, MTPs usually form three clusters (Zn-CDF, Fe/Zn-CDF, and Mn-CDF) according to their substrate specificity or seven groups (1, 5, 6, 7, 8, 9, and 12) according to the results of the phylogenetic analysis and annotation of Arabidopsis MTPs [7]. In the present study, 12 *CrMTP* genes were successfully identified from the *C. rosea* genome, which was similar to that of other Arabidopsis (12) and rice (10) [7]. Compared with MTP family identification of other plant species, only two gene pairs (CrMTP8/8.1, CrMTP9/9.1) showed gene expansion, and different subfamilies of Zn-CDFs (five), Fe/Zn-CDFs (five), and Mn-CDFs (two) were reasonable. Thus far, along with whole-genome sequencing in plants, the *MTP* gene families have been systematically identified in several plant species. For example, the typical gene expansion of the *PtrMTP* family was obvious in *Populus trichocarpa*, which contained 22 *PtrMTP* members [51]. The MTP family in *Brassica rapa* var. *rapa* (turnip) was more expanded than that in Arabidopsis, including 18 *BrrMTP* members [54]. *N. tabacum* contained at least 26 *NtaMTPs*, which were mostly due to its allotetraploid feature. Correspondingly, in *N. sylvestris* and *N. tomentosiformi*s, there were only 13 *NsyMTPs* and 12 *NtoMTPs* that could be detected in their diploid genomes [52]. Similarly, there were at least 20 *TaMTP* sequences in modern wheat *Triticum aestivum* (hexaploid, AABBDD) [56]. However, in sweet orange (*Citrus sinensis*) and grape (*Vitis vinifera*) genomes, there were 12 and 11 members of CitMTPs and VvMTPs, respectively [14,57]. Additionally, there were 13 *CsMTPs* identified from Mn hyperaccumulator tea plants (*Camellia sinensis*) [53]. *Canavalia rosea* is a typical diploid species with 11 pairs of chromosomes and 12 *CrMTP* members in total. Our findings in this study suggest that the *C. rosea* MTP family is relatively conserved in terms of the number and type of members.

As a mangrove-associated species, *C. rosea* holds great potential for HM storage and recycling, similar to other halophyte or mangrove species [41,46,58]. Exploring the functional characteristics of the metal ion transporters in *C. rosea* is of great interest. *Canavalia rosea* is a seashore halophytic plant with excellent extreme saline–alkaline resistance and can be used as an ecological restoration pioneer species, both on artificial islands and reefs for vegetation construction and on estuaries and littoral zones for HM phytoremediation in tropical and subtropical regions. After *CrMTP* genes’ identification, the expression patterns of 12 *CrMTP* genes of *C. rosea* were analyzed (Figure 7, Figure 8, Figure 9, Figure 10). The results demonstrated variation in the transcriptional levels among different *CrMTP* clusters. In general, three *Zn-CDF* members (*CrMTP1*, *CrMTP4*, *CrMTP5*, and *CrMTP12*) and two *Fe/Zn-CDF* members (*CrMTP6* and *CrMTP7*) in *C. rosea* showed almost constitutively high expression in all tested tissue types, and only two Mn-CDF members (*CrMTP8* and *CrMTP8.1*) also had a similarly high expression level (Figure 7). Highly expressed genes usually play important roles in plant development and stress responses. Our results indicated that these eight *CrMTP*s might be important regulatory factors for *C. rosea* plant growth. In contrast, the other four Mn-CDF members (*CrMTP9*, *CrMTP9.1*, *CrMTP10*, and *CrMTP11*) were expressed at obviously different levels in the five tested tissue types of *C. rosea*, implying that these *CrMTP* genes could be transcriptionally regulated by growth and developmental specific factors and that they might have specific functions in those corresponding tissues. The *CrMTP* expression patterns generally varied slightly under different HM treatments both in leaves and roots (Figure 8), which was quite different from previous reports [38,53,54], in which some *MTP*s were obviously upregulated following exposure to excessive amounts of some HMs. In some plant species, the identified *MTP* members showed relatively stable expression patterns even under multiple HM challenges, and only a small minority of HM stressors could affect the expression of *MTP*s [51,52]. In this study, most *CrMTP*s showed relatively higher expression levels in seedling leaves than in roots (Figure 8), indicating that the HM diffusion mediated by MTPs in aerial parts is more necessary and important than that in underground parts when under HM challenge. Conversely, as CrMTPs are dynamically regulated endomembrane transporters for cations, the changeable expression in roots seems to be more effective for controlling the absorption or diffusion of excess HMs.

*Cis*-acting elements are a series of nucleotide motifs that bind to specific transcription factors (TFs), thereby regulating transcription in plants [59]. The expression pattern of genes mostly depends on the way that TFs bind to the promoter regions. Here, the numbers and categories of *cis*-acting elements in *CrMTP*s’ promoter regions were systematically analyzed and summarized. Most of the promoter regions of *CrMTP*s contained some specific *cis*-acting elements, such as ABRE, MYC, MYB, MBS, and TC-rich repeats, which are involved in binding some specific TFs or responding to some biotic or abiotic stressors. Three are also some hormone-related responsive elements, suggesting that the *CrMTP*s could be regulated or affected by different stressors (Figure 6). Three kinds of metal-responsive elements (MRE1, MRE2, and CuRE) were also manually analyzed in detail. The promoter regions of *CrMTP*s showed more variability than the gene coding region, which corresponded with their gene-specific expression pattern (Figure 5B, Figure 6). The contribution of single *CrMTP*s to HMs or other stress tolerances and environmental adaptations in *C. rosea* needs to be explored further.

*Canavalia rosea* is highly adaptive to tropical coastal areas and therefore can be used as pioneer species for ecological restoration of the intertidal zones and coral islands or reefs. Although there is limited experimental evidence that plant MTPs are involved in high salinity and drought tolerance, some previous reports indicated that MTPs might be related to some adaptations to abiotic stressors, except HMs. The expression of Arabidopsis *MTP*s was determined under various abiotic stressors in both root and shoot tissues by RNA-seq, and *AtMTP9* showed the highest level of expression in response to salt and high osmotic stress [57]. A cation diffusion facilitator gene from soybean, *GmCDF1* (*GmMTP12*), negatively regulates salt tolerance by regulating K^+^-Na^+^ homeostasis [60]. A novel CDF (MceT) identified from a moderate halophile, *Planococcus dechangensis*, evolved from a traditional Zn^2+^ diffusion facilitator and acted as a Na^+^(Li^+^, K^+^)/H^+^ antiporter [61]. In our study, further habitat-specific RNA-seq data indicated that five *CrMTP* members could be transcriptionally regulated by special habitats with high salinity and drought characteristics (YX sample) (Figure 9), which might be an adaptive consequence for *C. rosea* growing and breeding in tropical coral islands or reefs.

The roles of MTPs have been extensively researched previously in many other plant species. These proteins are mainly involved in the transmembrane transport of divalent metal ions, mainly including ion absorption and efflux, cellular distribution, and other stress response. The first identified plant MTP was AtMTP1, which was demonstrated to be a transition metal transporter that extrudes metal ions from the cytoplasm to vacuolar regions, playing pivotal roles in maintaining zinc homeostasis in Arabidopsis [62]. Subsequently, several *MTP* members were identified from metal hyperaccumulator plants *Stylosanthes hamata*, *Arabidopsis halleri*, and *Thlaspi goesingense* [33,35,37]. These *MTP*s have certain features in common, that is, their relatively high expression in vivo is positively associated with their metal accumulating characteristic. Another experimental system with yeast as a host for a heterologous expression assay also confirmed this conclusion [23,28,33]. Here, the metal substrate specificity of CrMTPs was detected with a series of yeast mutant strains. Our results indicated that CrMTP1 is a typical Zn transporter with a slight capability for Cd and Co transport (Figure 10A–C). Similar findings were also found in rice OsMTP1 [3,23], and as a consequence, *OsMTP1* showed great Cd hyperaccumulating activity in tobacco and was endowed with promising potential for phytoremediation [12]. Although CrMTP4, CrMTP5, and CrMTP12 were classified as Zn-CDF, they did not show Zn transport in yeast, which was consistent with previous studies [51,52]. Although two Fe-CDFs (CrMTP6 and CrMTP7) were not cloned successfully, CrMTP10 showed typical Fe transporting capability in yeast (Figure 10F). Three Mn-CDFs (CrMTP8.1, CrMTP10, and CrMTP11) were cloned, while only CrMTP10 showed a Mn-transporting capability in yeast (Figure 10E). CrMTP4, CrMTP5, CrMTP8.1, CrMTP10, and CrMTP11 showed various degrees of a Ni-transporting capability, indicating that these members possessed metal substrate specificity (Figure 10D). This may be due to their unique amino acid composition in their His-loops (CrMTP4 and CrMTP5) or other loops and transmembrane domains. In general, this protein family might fulfill an important function in HM tolerance and regulate the environmental adaptation of *C. rosea* to tropical coastal regions, although more research is needed for further clarification.

## 4. Materials and Methods

### 4.1. Plant Materials and Stress Treatments

*Canavalia rosea* plants growing in the South China Botanical Garden (SCBG, 23°18′76′′ N, 113°37′02′′ E) and leaf samples taken from *C. rosea* plants growing on Yongxing Island (YX, 16°83′93′′ N, 112°34′00′′ E) were used in this study. The seeds of *C. rosea* were gathered from coastal regions of Hainan Province, China, and then cultivated under normal conditions (22–26 °C with a photoperiod of 16 h light/8 h darkness, and 50–60% relative air humidity) in a growth chamber, in which the cultured plants were kept in artificially controlled, optimum growing conditions. The seeds were germinated and cultivated in a soil/vermiculite mixture for one month before exposure to the various heavy metal (HM) or other stressors for further transcriptional analyses of *CrMTP* members. In brief, *C. rosea* seedlings with similar growth vigor and status were used in the following treatments. The seedlings were removed from the pots and carefully washed with distilled water to remove soil from the roots and transferred into different treatment solutions (1/2 Hoagland solution). For HM stress treatments, *C. rosea* seedlings were subjected to 0.1 mM CdCl_2_, 0.5 mM ZnSO_4_, 1 mM MnCl_2_, and 0.1 mM CuSO_4_ with the roots submerged. For high salinity and ABA treatment, seedlings were soaked in 600 mM NaCl solution (1/2 Hoagland solution). For ABA treatment, the *C. rosea* seedling leaves were sprayed with a freshly prepared working solution of 100 µM exogenous ABA. Plant tissues were collected at different time points (2 h and 48 h for RNA-seq, and 2 h and 24 h for qRT-PCR). All samples were immediately frozen in liquid nitrogen and stored at −80 °C for subsequent gene expression analysis. Three independent biological replicates were used.

### 4.2. Identification, Ka/Ks Calculation, and Evolutionary Analyses of the CrMTP Family in C. Rosea

Whole-genome sequencing was performed with *C. rosea* plants growing in Hainan Province, China. The assembled genome data of *C. rosea* were annotated with different programs, including InterPro [63] and Pfam [64] databases for gene family identification and DIAMOND [65] and InterProscan [63] to acquire all *C. rosea* proteins’ information with conserved domains and motifs (e < 1*×*10^-5^). Subsequently, Pfam ID (Cation_efflux, PF01545) was used to search for CrMTP family members, and putative sequences of CrMTP proteins were identified and submitted to the NCBI Conserved Domain Database (https://www.ncbi.nlm.nih.gov/Structure/cdd/wrpsb.cgi, accessed on 1 May 2021) to confirm the presence of the Cation_efflux domain. Finally, candidate *CrMTPs* were named based on their sequence homology with Arabidopsis *MTP*s or other plant species *MTP*s and *C. rosea* genome annotation.

Metal tolerance protein (MTP) sequences from *Arabidopsis thaliana*, *Oryza sativa*, *Glycine max*, and *Populus trichocarpa* were obtained from the Arabidopsis Information Resource (TAIR, http://www.arabidopsis.org, accessed on 1 May 2021), the Rice Genome Annotation Project (RGAP, http://rice.plantbiology.msu.edu/index.shtml, accessed on 1 May 2021), and Phytozome (https://phytozome.jgi.doe.gov/pz/portal.html, accessed on 1 May 2021), respectively. The obtained *MTP* nucleotide and protein sequences from *C. rosea* and *G. max* are listed in Table S1. The protein sequences of 12 AtMTPs from Arabidopsis, 10 OsMTPs from rice, 22 PtrMTPs from *P. trichocarpa*, 24 GmMTPs from *G. max,* and 12 CrMTPs from *C. rosea* were used to construct a neighbor-joining (NJ) phylogenetic tree using Clustal W and MEGA 6.0 software with 1000 bootstrap replicates.

The *CrMTP* genomic DNA and cDNA sequences were downloaded from the *C. rosea* genome database. Gene segmental duplication events of the *CrMTP* family were analyzed using MCScanX software (http://chibba.pgml.uga.edu/mcscan2/, accessed on 1 May 2021). The number of synonymous substitutions per synonymous site (Ka), the number of nonsynonymous substitutions per nonsynonymous site (Ks), and the probability (P-value) of Fisher’s exact test of neutrality were calculated using the Nei–Gojobori model with 1000 bootstrap replicates [66]. The diagrams of exon/intron organization, protein structure, chromosomal location, and gene duplication events were drawn by TBtools software [67]. The exon–intron structures within the coding sequences and the genomic sequences of each *CrMTP* gene were predicted with the Gene Structure Display Server (GSDS, http://gsds.cbi.pku.edu.cn, accessed on 1 May 2021). The conserved motifs of CrMTPs were detected using Multiple Em for Motif Elicitation (MEME) software (http://meme-suite.org/tools/meme, accessed on 1 May 2021), with the maximum number of motifs set at 10.

### 4.3. Protein Properties and Sequence Analyses

The molecular weight and isoelectric points of predicted CrMTPs were detected using the ExPASy proteomics server (https://web.expasy.org/protparam/, accessed on 1 May 2021). The TMHMM Server 2.0 program (http://www.cbs.dtu.dk/services/TMHMM/, accessed on 1 May 2021) and Protein Fold Recognition Server tool (PHYRE^2^, http://www.sbg.bio.ic.ac.uk/phyre2/html/page.cgi?id=index, accessed on 1 May 2021) were employed to predict the transmembrane helices and topologies of CrMTPs, and PHYRE^2^ was also used to perform the 3D prediction of CrMTPs. WoLF_PSORT and the online Plant-mPLoc server (http://www.csbio.sjtu.edu.cn/bioinf/plant-multi/, accessed on 1 May 2021) were used for subcellular localization prediction.

### 4.4. cis-Acting Elements Analysis of CrMTP Promoters

The promoter regions (2000 bp upstream from the translation start site, TSS) of all *CrMTPs* were retrieved from the genome database of *C. rosea*. The *cis*-acting elements present in these regions were predicted with PlantCARE (http://bioinformatics.psb.ugent.be/webtools/plantcare/html/, accessed on 1 May 2021). MRE1 (5′-TGCRCNC-3′) (metal-response element 1) [68], MRE2 (5′-HTHNNGCTGD-3′) (metal-response element 2) [69], and CuRE (5′-GTAC-3′) (copper response element) [70], which are related to metal stress, were also searched manually in each *CrMTP* promoter region. These *cis*-acting elements were summarized with Microsoft Excel 2010 software (Microsoft Corp., Albuquerque, NM, USA), and several selected *CrMTP* promoters were visualized using TBtools.

### 4.5. RNA-Seq of Different C. Rosea Tissues or under Different Stress Treatments

*C. rosea* RNA-seq datasets were constructed using Illumina HiSeq X sequencing technology. First, seven different tissues from *C. rosea* plants (root, vine, young leaf, flower bud, and young silique samples) were collected from *C. rosea* plants growing in the SCBG; mature leaf samples from *C. rosea* growing in SCBG and on YX Island were examined using FastQC (http://www.bioinformatics.babraham.ac.uk/projects/fastqc/, accessed on 1 May 2021) based on the primary 40 Gb clean reads and were mapped to the *C. rosea* reference genome using Tophat v.2.0.10 (http://tophat.cbcb.umd.edu/, accessed on 1 May 2021). Secondly, the *C. rosea* seedling tissues under different HMs and other abiotic stress challenges were also sequenced at the transcriptome level, and all EST information was mapped to the *C. rosea* reference genome. Gene expression levels were calculated as fragments per kilobase (kb) of transcript per million mapped reads (FPKM) according to the length of the gene and the read counts mapped to the gene: FPKM = total exon fragments/[mapped reads (millions) × exon length (kb)]. Expression levels of CrMTPs were visualized as clustered heatmaps (log2) using TBtools, which were directly shown with FPKM values by Microsoft Excel 2010.

### 4.6. Quantitative RT-PCR Analysis

Total RNA of the root, vine, and leaf samples were extracted separately using the plant RNA extraction kit (TIANGEN BIOTECH, Beijing, China) according to the manufacturer’s instructions. RNA concentration and quality were tested by NanoDrop 1000 (Thermo Fisher Scientific, Waltham, MA, USA), with the integrity checked on 0.8% agarose gel. The expression levels of 12 *CrMTP*s were determined by quantitative reverse transcription PCR (qRT-PCR), and three biological replications for each treatment were conducted. In brief, a total of 1 μg of RNA was reverse transcribed into cDNA in a 20 μL reaction volume using AMV reverse transcriptase (TransGen Biotech, Beijing, China) according to the supplier’s instructions. To quantify the relative transcript levels of selected *CrMTP* genes, qRT-PCR was performed with gene-specific primers using the LightCycler480 system (Roche, Basel, Switzerland) and TransStart Tip Green qPCR SuperMix (TransGen Biotech, Beijing, China) according to the manufacturer’s instructions. The gene-specific primers used for this analysis are listed in Table S2. All gene expression data obtained via qRT-PCR were normalized to the expression of *CrEF-1α* (Table S2).

### 4.7. Functional Identification with a Yeast Expression System

The full-length cDNA sequences of the *CrMTP* genes were obtained from the genome database of *C. rosea*. Then, the open reading frames (ORFs) of *CrMTP* genes were PCR amplified from different cDNA samples of *C. rosea* with gene-specific primer pairs (listed in Table S2). After several PCR procedures, the PCR fragments were purified and cloned into the *Bam*HI and *Eco*RI sites of pYES2 to yield recombinant plasmids of pYES2-CrMTPs and sequenced. The *Saccharomyces cerevisiae* wild-type (WT) strain BY4741 (Y00000) and five deletion mutants *ycf1∆* (Y04069), *pmr1∆* (Y04534), *cot1∆* (Y01613), *smf1∆* (Y06272), and *ccc1∆* (Y04169) were obtained from Euroscarf (http://www.euroscarf.de, accessed on 1 May 2021). The double-mutant yeast strain *zrc1Δ*/*cot1Δ* was kindly provided by Yuan and Sanders [15]. The plasmids were introduced into yeast using the LiAc/PEG method [71]. Yeast growth and metal sensitivity tests were performed as previously described, with minor modifications [23]. Briefly, transformed yeast was grown on solid SDG–Ura medium (synthetic dropout medium plus 2% galactose, uracil deficiency) plates for two days, and single colonies of yeast transformants were selected and used to inoculate liquid SDG–Ura medium. It was then incubated overnight or longer at 30°C, diluted with fresh prewarmed SDG medium (volume ratio 1:10), and then incubated with vigorous shaking for two days at 30 °C to reach an optical density of 2 at OD600 (optical density at 600 nm). The cells were then serially diluted in 10-fold steps, and 2 μL aliquots of each were finally spotted onto SDG medium plates with or without metal stressors. Plates were incubated at 30 °C for two to five days and photographed.

### 4.8. Statistical Analyses

All analyses were conducted in triplicate, with the results shown as the mean ± SD (*n* ≥ 3). The Excel 2010 (Microsoft Corporation, Albuquerque, NM, USA) Statistics program was used to perform statistical analyses.

## 5. Conclusions

In this study, a total of 12 *CrMTP* genes were identified from the *C. rosea* genome using bioinformatic methods. Subsequently, according to their phylogenetic relationship with Arabidopsis MTP members, they were named and their corresponding gene structure, chromosomal location, duplication events, and conserved motif were analyzed. The 12 CrMTPs were divided into 3 clusters (Zn-CDF, Fe/Zn-CDF, and Mn-CDF) according to their protein sequences, which had various structures and substrate specificities. The expression profiles of *CrMTP* genes based on transcriptome data were also analyzed in detail to explore the possible biological functions. The expression profiles of *CrMTP*s varied with different degrees among *C. rosea* tissues and in response to a series of HM treatments, which suggested that *CrMTP*s performed a range of functions in specific tissues responding to environmental stress. Furthermore, RNA-seq data were used to verify whether *CrMTP*s played roles in the abiotic stress adaptation of different habitats. The functional identification of several CrMTP members in yeast confirmed substrate specificity for transporting transition metals. These results supply basic and important information for understanding the putative functions of *CrMTP*s in *C. rosea* involved in phytoremediation, which concerns HM pollution. Moreover, this is the first study to confirm that *CrMTP*s play possible roles in salt/alkaline tolerance, providing a basis for further understanding the role of *CrMTP* family in *C. rosea* plants’ adaptation to extreme adversity on tropical coral islands or reefs.

## Figures and Tables

**Table 1:** Identified CrMTPs of *Canavalia rosea* (Sw.) DC. and their molecular characteristics.

Gene	Protein Length	MW (kDa)	PI	GRAVY	TMHs&Topologies *	Subcellular Localization	
						Plant-mPLoc	WoLF_PSORT
CrMTP1	433	48.182	6.19	0.034	6/in to in	Vacuole	plas: 7, E.R.: 4, cyto: 1, mito: 1, vacu: 1
CrMTP4	396	43.767	6.18	0.146	6/in to in	Vacuole	plas: 5, vacu: 4, E.R.: 4, golg: 1
CrMTP5	363	40.758	7.31	0.071	5/in to out	Vacuole	plas: 6, nucl: 2, E.R.: 2, chlo: 1, cyto: 1, vacu: 1, golg: 1
CrMTP6	614	67.268	7.77	−0.084	2/out to out	Vacuole	chlo: 4, mito: 4, nucl: 2, golg: 2, cyto: 1, plas: 1
CrMTP7	431	47.853	7.32	−0.013	4/in to in	Vacuole	plas: 5.5, E.R.: 4, cyto_plas: 3.5, mito: 2, chlo: 1, pero: 1
CrMTP8	506	56.719	6.11	−0.013	4/out to out	Mitochondrion; Vacuole	chlo: 6, mito: 2, E.R.: 2, nucl: 1, cyto: 1, plas: 1, extr: 1
CrMTP8.1	409	46.004	5.25	−0.010	4/in to in	Vacuole	plas: 7.5, cyto_plas: 4.5, vacu: 3, E.R.: 3
CrMTP9	412	46.950	6.84	−0.119	3/out to in	Vacuole	plas: 8, E.R.: 5, golg: 1
CrMTP9.1	396	45.485	7.32	−0.072	5/in to out	Cell membrane; Vacuole	plas: 10, vacu: 2, cyto: 1, E.R.: 1
CrMTP10	398	45.777	6.48	−0.137	5/in to out	Cell membrane; Vacuole	plas: 12, vacu: 2
CrMTP11	396	45.080	5.08	0.008	4/out to out	Cell membrane; Vacuole	plas: 12, vacu: 2
CrMTP12	863	97.802	7.16	−0.079	13/out to in	Vacuole	plas: 12, chlo: 1, pero: 1

* Here, the TMHs&Topologies analyses uses the prediction results generated from TMHMM Server V. 2.0 (http://www.cbs.dtu.dk/services/TMHMM/, (accessed on 1 May 2021)).

**Table 2:** Ka/Ks analysis and duplicated type calculation for *CrMTP* genes.

Duplicated Pair	Duplicate Type	Ka	Ks	Ka/Ks	Positive Selection
CrMTP8/CrMTP8.1	Segmental	0.0961	0.4000	0.2403	No
CrMTP9/CrMTP9.1	Segmental	0.0610	0.606	0.1006	No

## Data Availability

Data are contained within the article.

## Supplementary Materials

The following are available online at https://www.mdpi.com/article/10.3390/plants10071340/s1, Figure S1: The molecular structures of CrMTPs, Figure S2: The FPKM values histogram of RNA-seq data in this study, Figure S3: Quantitative RT-PCR detection of the expression levels of *CrMTPs* responding to different stresses (600 mM NaCl and 100 mM ABA) in *C. rosea* seedling plants, Figure S4: Simple map of CrMTPs-pYES2, Table S1: The sequences of *CrMTP* genomic DNA, CDS, and promoter region DNA, Table S2: Primer sequences used in this study.
