# Supplementary material for: Genome-Wide Identification, Structure Characterization, Expression Pattern Profiling, and Substrate Specificity of the Metal Tolerance Protein Family in Canavalia rosea (Sw.) DC
Source: Plants (Basel). 2021 Jun 30;10(7):1340. doi: 10.3390/plants10071340 (PMC8309081; doi:10.3390/plants10071340)
Supplement: Supplementary file 1 [file plants-10-01340-s001.zip › plants-1261613-supplementary.pdf]

Supplementary Material Information

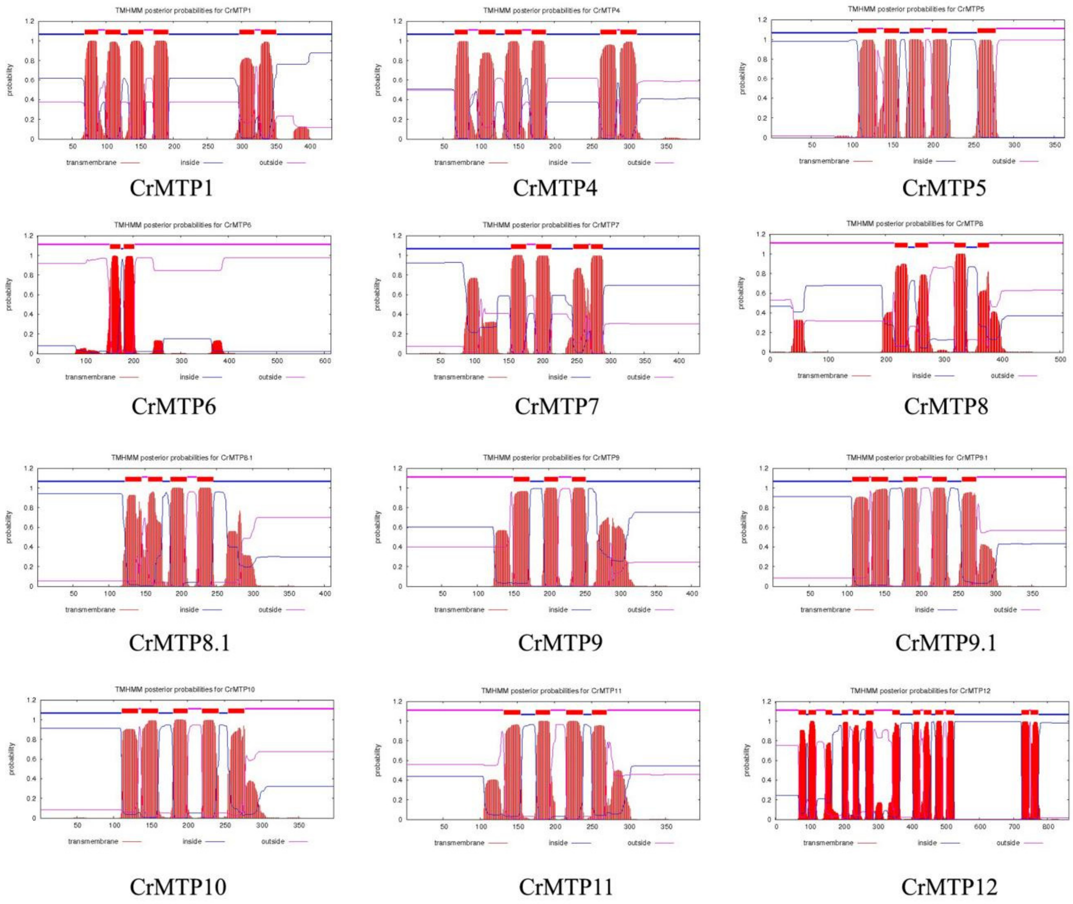

(A)

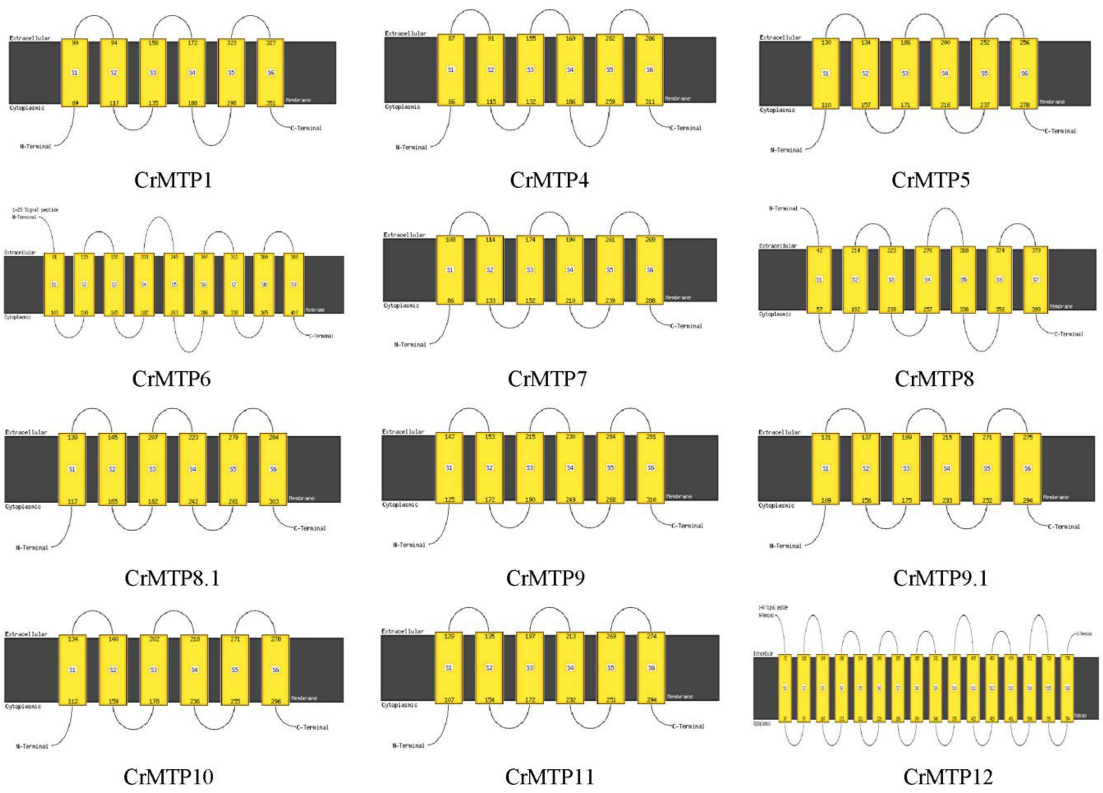
Extracellular
Cytoplasmic
Membrane
N-Terminal
C-Terminal
CrMTP1
CrMTP4
CrMTP5
CrMTP6
CrMTP7
CrMTP8
CrMTP8.1
CrMTP9
CrMTP9.1
CrMTP10
CrMTP11
CrMTP12

(B)

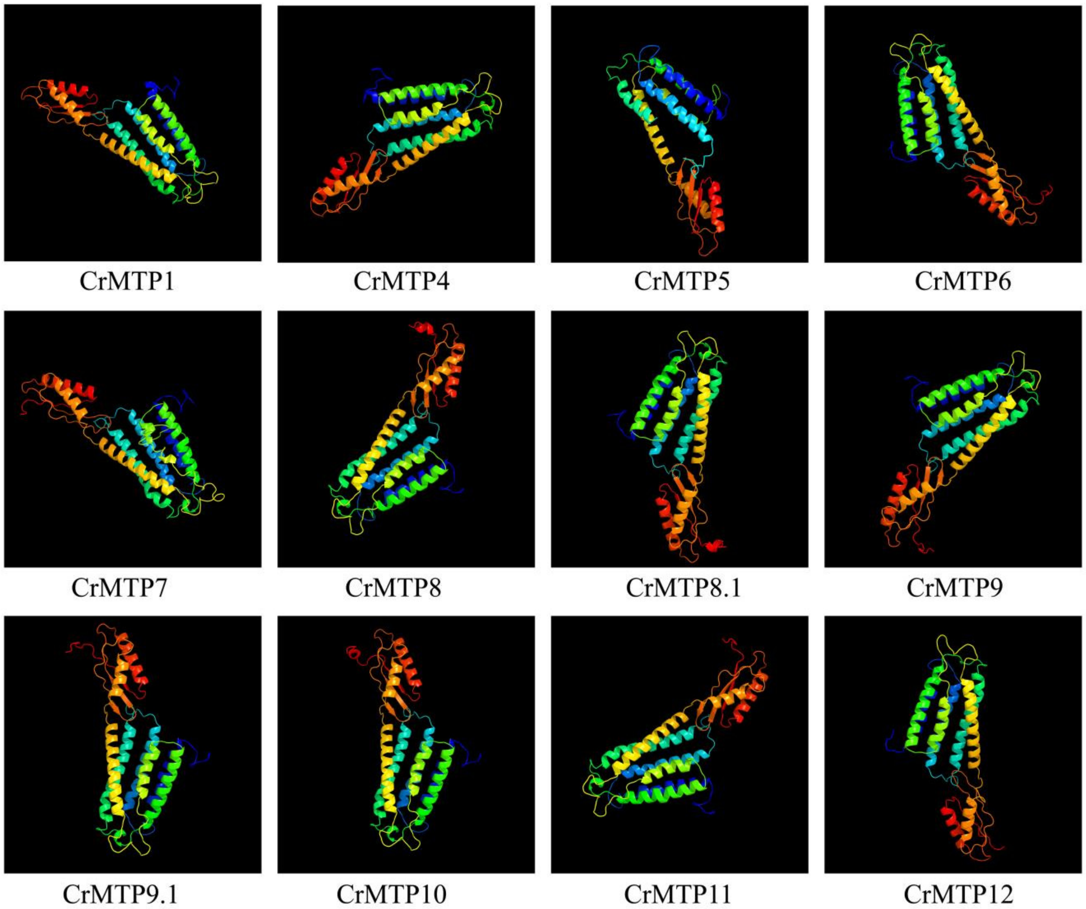

(C)

Figure S1. The molecular structures of CrMTPs. The putative transmembrane helices (TMHs) predicted using TMHMM Server V. 2.0 (http://www.cbs.dtu.dk/services/TMHMM/) (A), or TMHs structures (B), and 3D models (C) of CrMTPs constructed using Phyre2 (http://www.sbg.bio.ic.ac.uk/~phyre2/html/page.cgi?id=index).

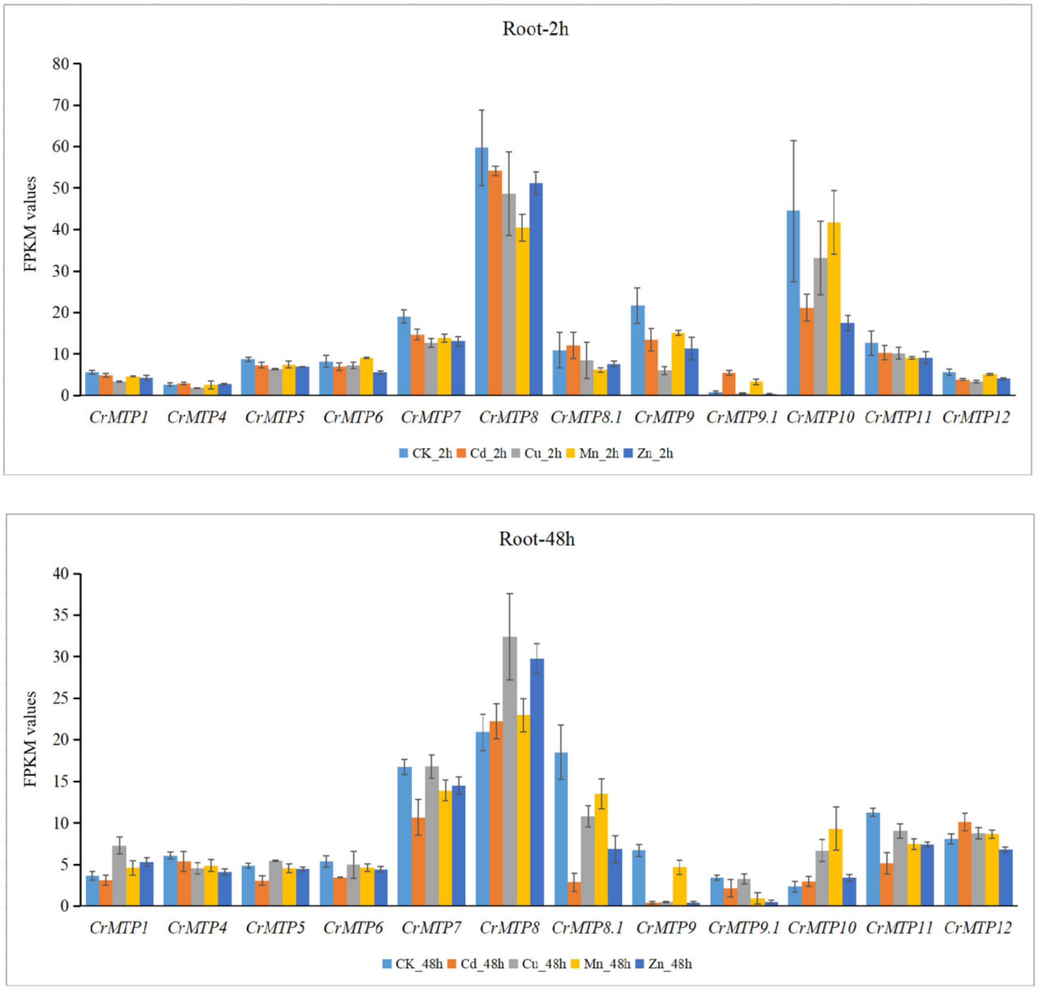

Root-2h
FPKM values
80
70
60
50
40
30
20
10
0
CrMTP1
CrMTP4
CrMTP5
CrMTP6
CrMTP7
CrMTP8
CrMTP8.1
CrMTP9
CrMTP9.1
CrMTP10
CrMTP11
CrMTP12
CK_2h
Cd_2h
Cu_2h
Mn_2h
Zn_2h
Root-48h
FPKM values
40
35
30
25
20
15
10
5
0
CrMTP1
CrMTP4
CrMTP5
CrMTP6
CrMTP7
CrMTP8
CrMTP8.1
CrMTP9
CrMTP9.1
CrMTP10
CrMTP11
CrMTP12
CK_48h
Cd_48h
Cu_48h
Mn_48h
Zn_48h

(A)

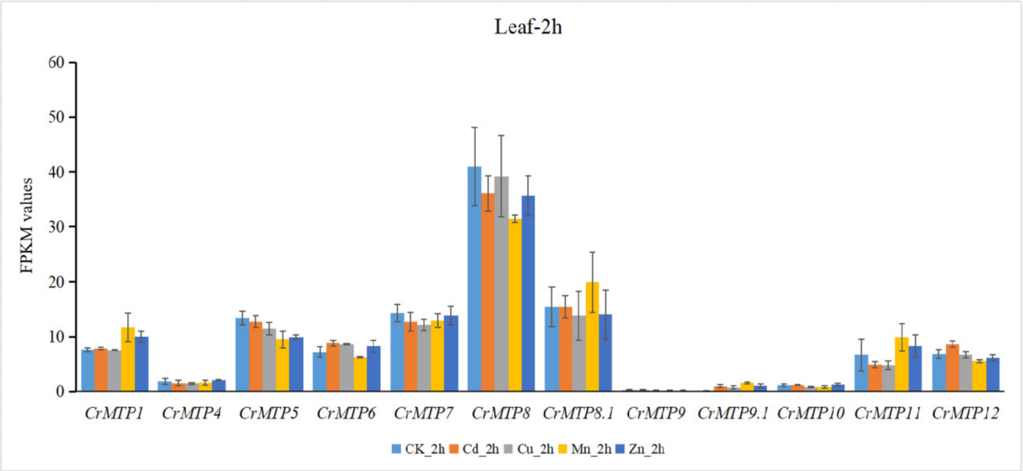

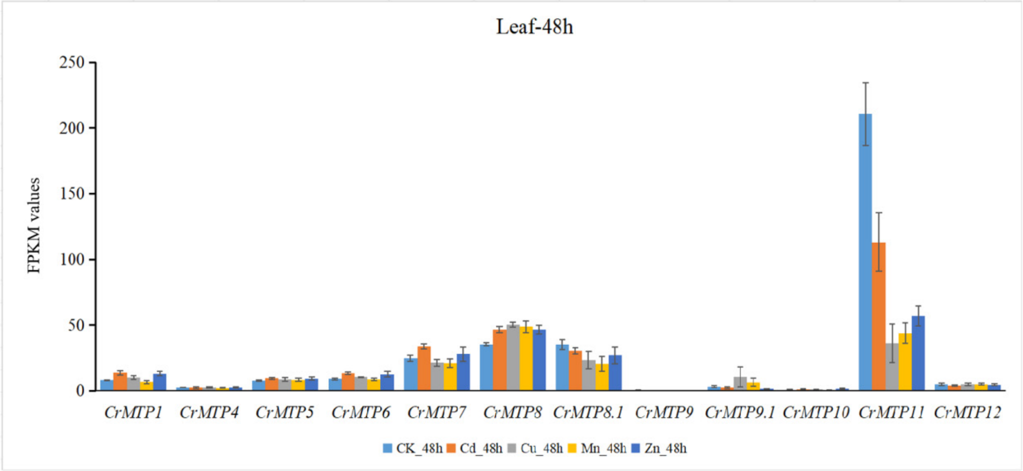

(B)

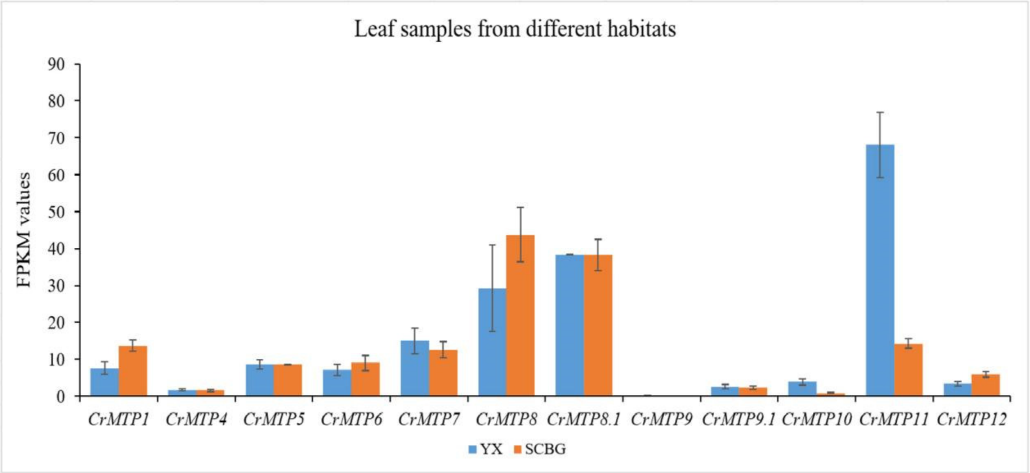

(C)

Figure S2. The FPKM values histogram of RNA-seq data in this study. (A) the expression patterns of *CrMTPs* under different HM challenges for 2 h or 48 h in root samples; (B) the expression patterns of *CrMTPs* under

different HM challenges for 2 h or 48 h in leaf samples; (C) the expression patterns of *CrMTPs* in leaf samples captured from YX Island (YX) and South China Botanical Garden (SCBG).

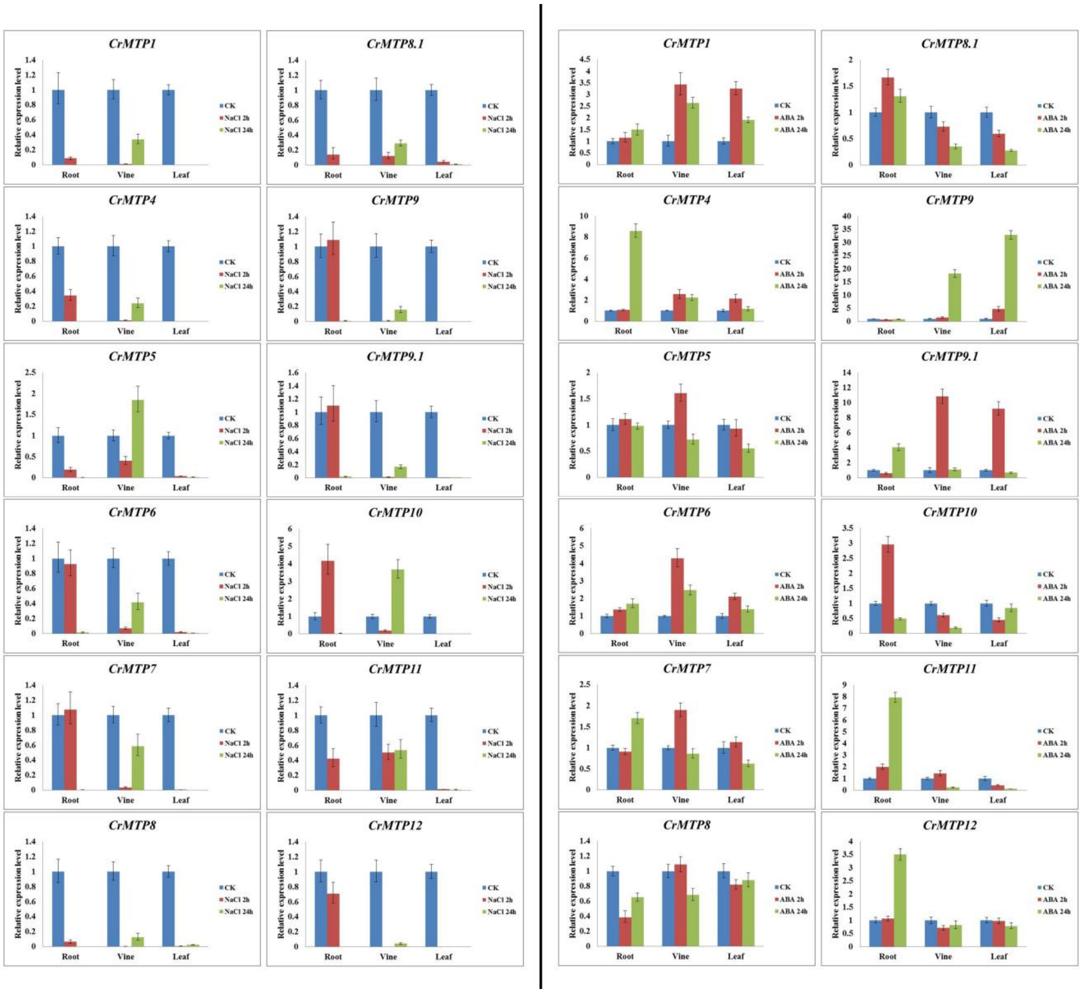

Figure S3. Quantitative RT-PCR detection of the expression levels of *CrMTPs* responding to different stresses (600 mM NaCl and 100 mM ABA) in *C. rosea* seedling plants. Relative expression values were calculated using the 2−ΔCt method with housekeeping gene *CrEF-α* as reference gene.

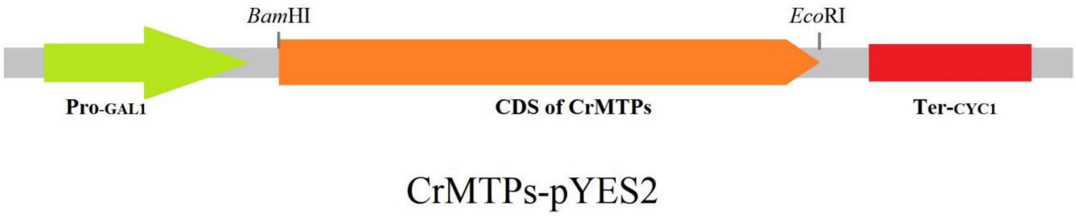

Figure S4. Simple map of CrMTPs-pYES2.

Table S1. The sequences of *CrMTP* genomic DNA, CDS, and promoter region DNA.

**The sequence information of the CrMTP family**	
**Protein (N-terminal to C-terminal)**	
CrMTP1	MVFLQLKMLHFHDLLQMEAQSSQDTQVIEISRDFPNVGRKVCGEAPCGFADAGSISKDSEERSTSMRKLLMAV<br>VLCVIFMTIEVIGGIKANSLAILTDAAHLLSDVAAFAISLFSLWAAGWEATPRQSYGFFRIEILGALVSIQLI

CrMTP4	WLLAGILVYEAIDRIIAGPKNVDGFLMFLVAAFGLVVNIIMALLLGHDHGHGHGHDGHGHGHDGHGHGHGHGH<br>DGHGHSHGILVSTHQHHDAKHTKDEHHHTHDDHIHHHDEKHSNYAHHPHKELTDPLLGESKDGRKKKKKQWNI<br>NVQGAYLHVLGDSIQSVGVMIGGGLIWYHPEWQIVDLICTLIFSVIVLGTTINMLRNILEVLMESTPREIDAT<br>QLERGLLDMEDVVAVHELHIWAITVGKILLACHVKIRREADADMVLDKVIDYIKRVYNISHVTIQIER<br>MKQMEHEKAPILEARSMQEIEIPIASERPDILPMKAESSGNCLCPFSRQENSDVASKESSKSAKKLSWLIVFY<br>AIVMVVEIIGGIKSHSLAVISDAAHLLSDIAGFAISLFAVWASGWEATPHQSFGYNRLEVLGALLSVQLIWLI<br>SGFLIYEAVGRIVLRNAKVNGKLMFAIAAFGFVLNAIMVAWLGHDHSHHHGCGNSNHHHSHHHGCGNSGHHHS<br>HHHTDHHHGNEELSKTTDEENLTLVSSSQKNTNVLNINLQGAYLHVMTDMIQSIGVMIAGAIIWAEPEWFIVD<br>LICTLIFSVLSLSTTIPMLRNIYGILMERTPNEISISMLENGLRSIKGVQDVHDLHVWAITVGKVVLSCHVVA<br>EPGISSIDLLGTIKHYCEKTYQIQHVTIQIE
CrMTP5	MEARETPSSINGDFGFGNSDRRFAFSRQASFQQQPHTPISIDSNDSASRRPFLSRTDSCIGIPSGGHHHHYWS<br>AQDEKPSGLLQSSSFSSFVFSVFRNIRSGHRYMKRLFLMISLNVAYSTAELLIGLFTGRVGLVSDAVHLTFGC<br>GLLTFSLFVMAASRKKADREYTYGQLFLLFMSFSLAVEALHAFIQDESEHKHYLIVSAVTNLFVNLIGVWFFR<br>NYARINLAYRNAEDMNHHSVFLHVLADSFRRVQNAEVLCLGLVSVTVFMLVLPLFRATGGVLLQMAPPSIPTT<br>ALNKCLRQISAREDVLEVSQARFWEFVPGHVVGSLSIQVKKGMDDRPILEFVHGIYHDLGVHELTVQTDDA
CrMTP6	MGFRLRILNPTYRTCITRLSSSKFVPPVLEPLNLSSLESCFNSSENPAFKIPKRWHLGHSHHDDYDHHHHLRQ<br>NQKEGENIFRLGLAADIGLATGKAITGYLSGSTAIIADAAHSISDVVLSGIALLSFKVAKAPRDKEHPYGRLI<br>CIHTLLHLSLIHLVSVSFMIIFVCHCISGHGKFETLGALGISCMLLATGGGIAWHAVDILMGLSSAGPEMVSQ<br>ALTHEHVHSDQHGGHHHGIDMDHPILALNMTIVSICAKEGLYWITKRAGEKQGSGLMKANAWHHRADAISSLV<br>ALIGVGKELCSLKGYLDGPTIKEVYFRRRRTSMDNLRKRGDELVMQNYNHNDKRQQQIGVKGCRNHGYGGNKV<br>VEGVIIVGKLGGLAGGSILGVRFLDPLAGLLVSGMILKAGAETGYQSILELVDAAIPAQHLDPIKQTIMQVDG<br>VKGCHRLRGRRAGSYLYLDVNIEVDPFSSVSAAHDIGEYVRHQIHKSHPTVTEIFIHIDPAMSHVSPGTTDQP<br>DSWSRDMDQNGVVPAGDSNIEGIVSDIISSNFPQMSVERITRHMFQSKIVLQIEVSMPPDMPIRHAMKMAEQA<br>EKEILKAVSNIMHVGIQLRLGQPFPQINDT
CrMTP7	MRTSYSYNLLRSRLRHSHHQHLYCVLLVKPVDHHLPSPATTSSTFYSPFLFPRSFNAHRHSFHRSFFTRAKPA<br>KKIEFNDRHSQRAVTTALWCNFLVFSLKFGVWLGSSSHVMLAEVVHSVADFANQALLAYGLTSSRRAPDALHP<br>YGYSKERFVWSLISAVGIFCLGSGATVVNGVQNLWTAQPPENMQYAAVVICGSFIIEGASLIVAIQAVKKGAA<br>AEGMKLRDYIWRGHDPTSVAVMTEDGAAVTGLVIAGASLVAVNVTGNAIYDPIGSIVVGNLLGMVAIFLIQRN<br>RHALIGRAMDDHDMERVLQFLKNDPVVDSLYDCKSEVIGPGFFRFKAEIDFNGVMVVQNYLKRTGREEWARQF<br>REATKEKDDTALVEIMSNYGEEVVIALGSEVDRLEKEIQNIVPGIRHVDIEAHNPTEATLSSESSQ
CrMTP8	MGKQTIPVKSVTYALSPFQQKVMTGLWKDLPTKIHHKISENWISATLLLSPLVGVYTYVQNYQEKEKLAHRSD<br>CRASMRHAASACFSASVIVKRPLLGNQNQNENHDGNSFNSLRTAFLSKLPDKLRSGLDSESPFEFDHHLSATS<br>SLSQGEKEYYGKQFATLKSFEEVDSIVTADCTDVEDIEKQAQHERAMKISNYANAALLAMKIYVTIRSGSIAV<br>AASTLDSLLDFMAGGILWFTHMAMKDVNMYKYPIGKLRVQPVGIIIFAAVMATLEQLKSIHIKENPVPKAPTL<br>WGFQVLITALEQVIENNPSEKMSYDQLVWLYSIMILATVVKLSLWLYCRSSGNKIVRAYADDHHFDVVTNVVG<br>LVAAVLGDQFYWLIDPVGAILLAIYTITNWSGTVMENAVSLVGQSAPPEVLQKLTYLVIMHSQIKRIDTVRAY<br>TFGVLYFVEVDIELPEDLPLKEAHAIGESLQIKLEKLPEVERAFVHLDFECDHKPEHSVLNNLPDTKP
CrMTP8.1	MDRNSGSDPTWPLLAKLDPNSADNGSRRGRLNRRNSVNSLRAAFLSKLPEKVRSGIDSEAPFDTDLSSTTALS<br>KGEKEYYERQFATLKSFEEVEYMVSPDYVVEEEDNEEQAQQERAMKISNYANIALLILKIYATVRSGSIAIAA<br>STLDSLLDLMAGGILWFTHVAMKNINIYQYPIGKLRVQPVGIIIFAAVMATLGFQVLITAAEQLIENSPAEKM<br>TAEQLIWLYSIMLFATVVKFVLWLYCRSSGNQIVRAYADDHRFDVITNVVGLVAAVLGDKYYWWIDPVGAILL<br>AIYTITNWSHTVMENAVSLVGQSAPPEVLQKLTYLVIRHPQVKRIDTVRAYTFGVLYFVEVDIELPEDLPLKE<br>AHAIGETLQIKLEKLPDVERAFVHLDFECDHKPEHSVLSKLPNN

CrMTP9	MVMSAGTSGHGGAIAETGRREQLLVENSTGDTSSFSWRLNLKEFQLPQQTHNNHNDRHHHHPLSFTFHGLLRK<br>PKKQRKVAEYYKKQERLLEGYNDMDTMTETGYFPGSLTEDETKQLAKSERLAINVSNAANLVLFAAKVYASIE<br>SKSLAVIASTLDSLLDLLSGFILWFTANAMRNPNHYHYPIGKKRMQPVGIIVFASVMATLGLQILIESGRQLI<br>SKSKPEMDLDELKWMIGIMVSVTVVKFILMVYCRRFKNEIIRAYAQDHFFDVITNSVGLAAAVLAVKCSWWID<br>PIGAIIIAVYTINTWAKTVIENVWSLIGRTAPPDFLGKLTYLIWNHHEEVRHIDTVRAYTFGAHYFVEVDIVL<br>PEDMPLNQAHNIGETLQEKLEQLPQVERAFVHIDFEFTHRPEHKMMV
CrMTP9.1	MAESGGGPRRREPLLVSPEEEAANPSWRLNVKEFRLPTQTNDQQHHHSFTFRGLLRKPRKQRKVAEYYKKQER<br>LLEGFSEMETMTETGDFPGSLTEDEMKQLAKSERMAVHVSNVCNLVLFAAKVYASIASRSLAVIASTMDSLLD<br>LLSGFILWFTAHAMKTPNHYNYPIGKKRMQPVGIIVFASVMATLGLQILIESARELISKSKPDMDPVKLHWMI<br>GIMVFVTVVKFVLMIYCRRFKNEIVRAYAQDHFFDVITNSVGLAAAVLAVKFYWWIDPTGAIIIALYTINTWA<br>KTVIENVWSLIGRTAPPDFLAKLTYLIWNHHEQIKHIDTVRAYTFGAHYFVEVDIVLPEDMLLHQAHNIGETL<br>QEKLEQLPEVERAFVHIDFEFTHRPEHKTMV
CrMTP10	MGTELRTDSSDYRTELLSPTIAAENVSMARQPSWRINMDEHRLPERQMESHFGFGFFLRTLKRQRKLAQYYKR<br>QEGLLKGYQEVDSYHDLGMLPGNLTEDEMKELERNEKVAIYASNIGNMVLFVAKVYASIQSRSLAVIASTLDS<br>LLDLLSGFILWFTAYAMSKPNHYQYPIGKNRMQPVGIVVFASVMATLGLQILFESAREIITKAQPERDPVKEK<br>WMIGIMVTATLVKVVLMTYCRRFKNEIVRAYAQDHFFDVITNSIGLATAVLAIKFYWWLDPVGAILIALYTIS<br>NWAKTVMENVWSLIGKTAPPEYLAKLTYLCWNHHKEIKHIDTVRAYTFGSNYFVEVDIVVSEEMSLNEAHDIG<br>ETLQDKLEKLPEIERAFVHIDLNTTHKLEHKSN
CrMTP11	MLEPVELRSEEQRSLLLDSNNGDRSWRLNFDGFQISSEHTEKQVKPPRGLHDCYGVLGQEDNIAEYYQQQVEV<br>LEGFNEMDVLAERGFVPGMSKEERDKLARNETFAIRLSNVANMVLFAAKVYASVRSGSLAIIASTLDSLLDLL<br>SGFILWFTAFSMQTPNPYQYPIGKKRMQPLGILVFASVMATLGLQIILESIRTLISSDNAFSLTREQEQWVVG<br>IMLSVTLVKFLLMIYCRSFTNEIIKAYAQDHFFDVITNVIGLIAALLANYFDDWMDPVGAIILALYTIRTWSM<br>TVLENVNSLVGRSAAPEYLQKLTYLCWNHHKAIRHIDTVRAYTFGSHYFVEVDIVLPADMPLQEAHDIGESLQ<br>EKLELLPEIERAFVHLDYEFSHKPEHAQAHS
CrMTP12	MADQPHHHHHRPHRLSVPPRTATLATTPRPYPTVRYSTSASTPTATPSKHRRLSSSLSFSNSKSKSKSSLSSL<br>FLLLFSLRSLYSLLPFLRSSPSFSLFPFTFLVSLLSFFLTLSFSLFSSSSSSKDPFQQHKPKQSLLTVTHSQQ<br>RILVAKSIVLALVFLLRFQALRYCGTASMILAELMGSIAAQPKSQNRTKVRGFVSLFLGLFMLSFGWDRIECF<br>PVWGYAKCVRVWPLLLPFMSGFLACYERVSIMNWGTIKQLGHKRVRLITLFFTTIVLFVPAVISFFVLESEED<br>SVSFGNLVWPLANTVVFGVLLSENYSDDYVMSFKDSKREFLVTFVCTMVLELFYYPELSLWGLLLCGILLYFA<br>VRDLDPFHSNEFGSGEELFSDMIMRPIRHVLGERKSRKIALFLLINTGYMVVEFAVGFMSNSLGLISDACHML<br>FDCAALAIGLYASYISRLPANNHYNYGRGRFEVLSGYVNAVFLVLVGALIVVESFERILDPQEISTSSLLVVS<br>IGGLVVNVIGLIFFHDEHHHAHGLSGSCSHSHAHTEFHSHHSHHHHDLQCHSKHHHDERHGSNQEFISVSSDC<br>QDNSCSGDPGHHNHSSHGRKAEFHADNHCSKSFTHDHAQLHDCHEHCSHERMVECHVDNHSIKSIEHHGHQDH<br>HHGHQDHHHHHGHQDHHHHHGHQDHHHHHGHHDHHDHAQHHEYHDLHKAPLVHQKHNHRHIDHNMEGIFLHVL<br>ADTMGSVGVVISTLLIKYKGWLVADPACSIFISILIVSSVIPLLRNSAEILLQRVPRVHEHELKDVLTDVLKI<br>RGVYGIQKFHLWNFTNTDVVSTLHLHVSTDTDKTSAKSQVLHLLHSAGIKDLTLQVESVG
**CDS (5′ to 3′)**	
CrMTP1	ATGGTTTTTCTCCAACTCAAAATGTTGCATTTTCATGATCTATTGCAGATGGAAGCACAAAGCTCTCAGGATA<br>CCCAAGTTATTGAAATCAGCAGAGATTTTCCTAATGTAGGAAGGAAGGTTTGTGGGGAAGCACCTTGTGGGTT<br>TGCAGATGCTGGATCCATCTCTAAGGATTCTGAAGAACGATCTACATCAATGCGAAAGCTTTTAATGGCAGTG<br>GTCCTATGTGTTATTTTCATGACCATTGAAGTAATTGGTGGCATCAAGGCTAACAGTCTTGCGATATTGACTG<br>ATGCAGCACACTTGCTTTCAGATGTTGCAGCCTTTGCCATCTCCTTATTTTCTTTATGGGCTGCTGGATGGGA<br>AGCTACTCCTCGCCAATCGTATGGATTTTTCAGAATAGAGATTCTTGGTGCTTTGGTTTCTATCCAATTAATA

	TGGTTGCTTGCTGGAATTCTGGTATATGAAGCCATTGATAGAATCATTGCCGGTCCTAAAAATGTGGATGGCT<br>TTTTAATGTTTTTAGTTGCTGCATTTGGTCTTGTGGTTAACATCATCATGGCTTTGTTATTGGGTCATGATCA<br>TGGCCATGGACATGGACATGATGGGCATGGGCATGGACACGATGGGCATGGGCATGGACATGGACATGGACAT<br>GATGGGCATGGCCACAGTCATGGAATTTTAGTGTCTACTCATCAACATCATGATGCAAAGCATACAAAAGATG<br>AGCACCATCACACCCATGATGATCACATACACCATCATGATGAAAAGCATTCAAACTATGCTCATCATCCTCA<br>CAAAGAGCTTACTGATCCGCTTCTTGGTGAATCAAAAGATGGACGCAAGAAGAAGAAGCAATGGAACATA<br>AATGTACAGGGAGCTTATCTACATGTGCTTGGGGACTCCATCCAGAGTGTTGGGGTAATGATTGGGGGAGGAC<br>TCATATGGTATCATCCTGAATGGCAAATAGTTGATTTAATTTGCACTCTAATATTTTCAGTAATTGTTCTGGG<br>GACAACTATCAATATGCTGCGAAACATTTTGGAAGTCCTGATGGAGAGCACCCCACGCGAGATAGATGCTACT<br>CAGCTTGAAAGGGGGCTATTGGATATGGAAGATGTAGTGGCTGTTCATGAGCTGCACATATGGGCCATTACGG<br>TGGGAAAGATTTTGCTAGCTTGTCATGTTAAAATCAGACGTGAAGCAGATGCAGACATGGTCCTGGACAAGGT<br>TATAGACTATATCAAAAGGGTTTATAACATAAGTCATGTAACTATACAGATAGAGCGCTAG
CrMTP4	ATGAAGCAAATGGAACATGAGAAAGCCCCTATCTTGGAAGCAAGGTCCATGCAGGAAATTGAGATACCCATTG<br>CCTCTGAAAGACCTGATATTCTTCCTATGAAAGCGGAGTCATCTGGAAATTGTCTTTGTCCTTTTTCCAGACA<br>GGAAAACAGTGATGTGGCATCAAAAGAGTCATCAAAATCAGCAAAGAAACTTTCTTGGCTCATTGTTTTCTAT<br>GCTATTGTTATGGTTGTGGAAATTATTGGTGGTATAAAATCCCACAGCCTAGCTGTTATTAGCGATGCAGCAC<br>ACTTGCTTTCTGATATTGCTGGATTTGCTATCTCTCTCTTTGCAGTGTGGGCTTCAGGTTGGGAGGCAACACC<br>GCATCAATCTTTTGGATACAACCGTCTTGAGGTTTTGGGTGCTCTCTTATCCGTGCAGTTAATTTGGTTGATA<br>TCTGGTTTTTTGATATATGAAGCAGTTGGTAGAATTGTTCTCCGAAATGCCAAGGTGAATGGAAAACTAATGT<br>TTGCAATTGCAGCATTTGGGTTTGTTCTAAACGCTATCATGGTTGCGTGGCTTGGTCATGATCATAGTCATCA<br>TCATGGCTGTGGAAACTCGAATCATCATCACAGTCATCATCATGGCTGTGGAAACTCGGGTCATCACCACAGT<br>CATCATCATACAGATCATCATCATGGAAATGAGGAACTATCTAAAACAACTGATGAGGAGAACCTTACCCTGG<br>TTTCAAGTAGTCAAAAAAATACTAATGTCTTGAACATAAATCTCCAAGGGGCCTATTTACATGTCATGACTGA<br>TATGATTCAATCTATTGGAGTGATGATTGCTGGAGCCATAATATGGGCTGAACCGGAGTGGTTTATAGTTGAC<br>CTTATATGCACTCTTATATTCTCTGTTTTATCTTTAAGTACTACTATTCCCATGCTTAGGAATATTTATGGCA<br>TTCTGATGGAAAGGACACCAAATGAAATCAGTATCAGTATGCTGGAAAATGGCCTCAGAAGTATCAAGGGAGT<br>GCAGGATGTTCATGACTTGCACGTGTGGGCTATAACCGTTGGAAAAGTTGTTCTATCTTGCCATGTAGTGGCT<br>GAACCTGGTATCAGCTCCATTGATTTACTTGGCACGATTAAGCATTACTGTGAAAAAACATACCAAATACAAC<br>ATGTAACAATACAAATTGAGTAA
CrMTP5	ATGGAAGCCAGGGAGACTCCGAGTTCCATCAACGGTGATTTTGGGTTCGGTAATAGCGATCGGAGATTCGCGT<br>TTTCGCGCCAAGCTTCATTCCAGCAACAACCTCATACGCCGATTTCCATCGATTCGAACGATTCGGCTTCCAG<br>AAGGCCATTTCTATCCCGAACAGATTCCTGCATAGGCATTCCCTCCGGTGGCCACCACCACCATTACTGGTCT<br>GCGCAAGATGAAAAGCCCTCTGGTTTGCTGCAGAGTTCGTCTTTTTCTTCCTTCGTTTTCTCCGTTTTTCGAA<br>ACATTAGGTCTGGCCATAGGTATATGAAGAGGTTGTTTTTGATGATCTCGCTCAACGTTGCTTACTCCACCGC<br>CGAGTTGCTCATTGGCCTCTTCACTGGCCGTGTGGGTTTGGTGTCTGATGCAGTTCACTTGACTTTTGGATGT<br>GGTCTTCTCACGTTTTCGTTGTTTGTTATGGCTGCATCTAGGAAGAAAGCTGATCGAGAGTATACTTATGGAC<br>AGCTGTTTCTTTTGTTTATGTCATTCTCCCTTGCAGTTGAGGCACTTCATGCATTCATACAAGATGAATCGGA<br>GCACAAGCATTACTTGATTGTTTCTGCAGTCACCAATTTATTTGTGAATCTCATTGGTGTATGGTTCTTTAGA<br>AACTATGCTCGGATTAATCTTGCTTACAGAAATGCAGAAGATATGAATCACCACTCTGTTTTCCTGCATGTTC<br>TTGCTGATTCCTTTCGCAGGGTTCAGAATGCAGAAGTCTTGTGTTTGGGGCTTGTTTCTGTTACTGTTTTTAT<br>GCTTGTCCTACCTCTATTTAGGGCAACTGGTGGCGTCTTGCTCCAAATGGCACCTCCTAGCATCCCAACTACT<br>GCTTTGAACAAATGCTTGAGACAGATTTCTGCTCGGGAAGACGTACTGGAAGTCTCCCAGGCTCGATTTTGGG<br>AATTTGTGCCTGGACATGTGGTGGGCTCACTATCAATACAGGTAAAGAAGGGGATGGATGATCGCCCAATACT

CrMTP6	GGAATTTGTGCATGGCATATACCATGATTTGGGTGTGCATGAACTCACTGTGCAGACCGACGATGCATGA<br>ATGGGATTTCGATTGAGAATTCTGAATCCCACATATAGAACATGCATTACAAGGCTTTCCTCTTCAAAATTTG<br>TTCCTCCGGTGTTGGAACCACTCAATTTGTCTTCATTAGAGTCTTGTTTCAATTCCTCTGAAAATCCCGCGTT<br>CAAAATCCCCAAGAGATGGCATTTGGGTCATTCCCATCACGACGACTATGATCATCATCACCACCTCCGTCAG<br>AATCAGAAGGAGGGAGAGAATATTTTCCGGCTCGGACTCGCCGCCGACATTGGTTTGGCCACTGGAAAAGCCA<br>TCACTGGCTATCTTTCCGGAAGCACTGCCATTATCGCTGATGCTGCCCATTCCATATCTGATGTGGTTCTTAG<br>TGGCATAGCTTTGTTGTCCTTTAAAGTTGCCAAGGCGCCAAGAGATAAAGAACACCCATATGGTCGCTTGATT<br>TGTATCCACACGCTTCTCCATTTATCTCTTATACATCTAGTCTCTGTGTCATTTATGATTATATTTGTCTGCC<br>ATTGTATCTCAGGACATGGTAAATTTGAGACTCTAGGGGCTCTTGGAATCTCTTGCATGCTATTGGCGACTGG<br>GGGTGGCATTGCGTGGCATGCTGTAGACATTTTGATGGGATTGTCCTCCGCTGGTCCAGAAATGGTTAGCCAG<br>GCATTGACACATGAGCATGTGCATAGTGACCAACATGGTGGACATCACCATGGAATTGACATGGATCATCCTA<br>TCCTTGCTCTGAATATGACTATAGTGTCAATATGTGCTAAAGAAGGACTTTACTGGATAACAAAACGAGCTGG<br>TGAGAAGCAAGGTAGTGGACTAATGAAAGCAAATGCATGGCATCACCGTGCAGATGCAATTTCATCACTTGTT<br>GCTCTCATTGGAGTTGGTAAGGAACTTTGTTCTTTGAAAGGTTACTTGGATGGGCCTACAATTAAGGAAGTCT<br>ACTTTAGAAGGAGAAGGACAAGTATGGACAATTTGAGAAAAAGAGGGGATGAGTTAGTTATGCAGAACTATAA<br>CCATAATGACAAGCGGCAGCAACAGATTGGGGTTAAAGGATGCAGGAATCATGGATATGGGGGGAACAAGGTA<br>GTGGAGGGGGTGATCATTGTGGGAAAACTTGGGGGGTTGGCTGGAGGGTCTATTCTTGGAGTGAGATTTCTAG<br>ATCCACTTGCTGGACTTCTTGTCTCAGGCATGATTTTGAAAGCTGGAGCAGAAACTGGTTACCAAAGTATCTT<br>GGAATTGGTGGATGCTGCAATCCCAGCACAGCATTTGGATCCTATTAAACAAACAATAATGCAAGTTGATGGT<br>GTCAAGGGCTGTCATCGCTTAAGAGGAAGGAGAGCTGGTTCATATTTGTATCTTGATGTAAATATTGAGGTTG<br>ATCCTTTTTCCAGTGTTAGTGCTGCACATGACATTGGTGAATATGTTCGTCATCAAATTCACAAGTCGCATCC<br>TACCGTGACTGAAATTTTTATACACATAGATCCTGCAATGTCACATGTTTCTCCCGGCACAACAGATCAGCCA<br>GATAGTTGGAGCCGAGACATGGACCAGAACGGTGTTGTTCCAGCTGGGGATAGTAACATTGAAGGAATTGTTT<br>CTGATATCATCTCATCAAACTTTCCACAGATGTCAGTTGAGCGCATAACACGGCACATGTTTCAAAGCAAGAT<br>TGTTCTTCAAATTGAGGTTTCCATGCCACCTGATATGCCAATTCGACATGCAATGAAAATGGCAGAGCAAGCA<br>GAAAAAGAGATTTTGAAGGCTGTCTCAAATATAATGCATGTTGGCATTCAGCTACGTTTGGGGCAACCATTCC<br>CACAGATAAATGATACTTAG
CrMTP7	ATGCGAACATCCTATTCCTACAATCTTCTTCGCTCTCGCCTCCGCCACTCTCACCACCAACACTTGTATTGCG<br>TTCTACTTGTCAAACCAGTAGACCACCACCTACCATCTCCCGCCACCACCTCTTCCACTTTCTATTCACCCTT<br>CCTCTTCCCACGTTCCTTCAATGCTCATCGCCACTCATTTCATCGCAGTTTCTTTACAAGGGCCAAGCCAGCT<br>AAGAAAATTGAATTCAACGATCGCCACAGTCAGCGAGCTGTCACAACTGCATTGTGGTGTAACTTTCTTGTTT<br>TTTCTCTCAAGTTTGGAGTATGGTTAGGTTCCTCTAGTCATGTCATGTTAGCTGAAGTGGTGCACTCGGTTGC<br>TGATTTTGCAAACCAGGCACTACTTGCATATGGTCTAACTAGCTCAAGGCGTGCACCAGATGCCCTTCATCCA<br>TATGGCTATTCCAAGGAAAGATTTGTCTGGTCTTTGATATCTGCTGTTGGGATATTTTGTCTTGGTTCTGGTG<br>CTACTGTTGTTAATGGAGTTCAAAACTTGTGGACTGCACAGCCCCCTGAAAATATGCAGTATGCAGCTGTGGT<br>GATATGTGGTTCATTCATCATTGAAGGTGCCTCTCTTATTGTTGCCATACAAGCTGTCAAGAAAGGTGCAGCC<br>GCGGAGGGAATGAAATTAAGAGATTATATTTGGCGTGGCCATGATCCTACATCTGTTGCAGTGATGACAGAGG<br>ACGGTGCTGCAGTAACTGGCCTTGTTATTGCTGGGGCATCATTGGTTGCTGTGAATGTTACTGGAAATGCCAT<br>TTATGATCCCATAGGTTCAATCGTAGTTGGCAACTTGCTTGGAATGGTAGCCATATTTCTTATCCAGAGGAAC<br>CGACATGCTCTGATTGGTAGAGCTATGGATGACCATGATATGGAGAGAGTACTTCAATTTTTGAAAAACGATC<br>CGGTTGTAGATTCCCTCTACGATTGCAAAAGTGAAGTTATTGGGCCTGGATTCTTCAGATTTAAGGCAGAAAT<br>AGATTTTAATGGAGTGATGGTGGTGCAAAATTATCTTAAAAGGACTGGACGTGAAGAGTGGGCCAGACAGTTT<br>CGTGAAGCTACAAAGGAGAAGGATGATACGGCCCTAGTGGAGATTATGTCAAATTATGGTGAGGAAGTAGTTA

	TAGCTTTAGGAAGTGAAGTGGATAGGTTAGAGAAGGAGATCCAAAATATCGTACCTGGCATTCGGCATGTTGA
	TATTGAGGCCCACAATCCAACAGAGGCGACCTTGTCAAGTGAAAGTTCTCAATAA
	ATGGGGAAACAGACAATTCCGGTAAAATCGGTGACTTACGCACTCTCCCCTTTCCAGCAGAAGGTAATGACCG
	GTCTCTGGAAGGATCTACCAACAAAGATTCACCATAAGATCTCCGAAAATTGGATCAGCGCCACTCTGTTGCT
	CAGTCCCCTCGTCGGAGTCTACACGTATGTTCAAAACTACCAGGAGAAGGAGAAGTTGGCTCACAGGTCAGAT
	TGCAGAGCCAGCATGCGGCATGCAGCATCAGCATGCTTTTCCGCGAGTGTCATTGTTAAAAGGCCCCTACTGG
	GGAACCAGAACCAGAATGAGAATCATGATGGAAACTCTTTTAACTCGCTTAGAACCGCTTTCTTGTCCAAGCT
	TCCCGATAAGCTCCGTTCTGGCCTCGACTCTGAGTCTCCCTTTGAGTTTGATCACCATCTCTCCGCTACCTCC
	AGTTTAAGCCAAGGAGAGAAAGAATACTATGGAAAACAATTTGCTACTCTAAAATCATTTGAGGAAGTTGACT
	CCATAGTAACGGCTGACTGCACTGATGTAGAAGACATTGAAAAACAAGCTCAACATGAACGAGCAATGAAGAT
	TTCTAATTATGCAAATGCAGCTTTATTGGCAATGAAGATTTATGTGACAATAAGGAGTGGGTCAATAGCGGTT
	GCTGCATCAACATTGGATTCTCTGCTTGATTTCATGGCTGGTGGCATACTTTGGTTCACTCACATGGCAATGA
CrMTP8	AGGATGTAAATATGTACAAATACCCAATTGGAAAGTTGAGGGTGCAGCCAGTGGGCATAATTATCTTTGCGGC
	CGTCATGGCAACACTTGAGCAACTCAAAAGTATTCATATAAAGGAAAACCCAGTGCCCAAGGCTCCCACCTTG
	TGGGGCTTTCAGGTATTAATCACGGCTCTAGAACAAGTAATAGAAAATAATCCATCTGAGAAGATGTCTTATG
	ATCAACTAGTATGGCTGTACTCTATTATGATATTAGCAACAGTGGTGAAGCTTAGTCTCTGGCTTTACTGTAG
	AAGCTCAGGAAACAAAATTGTCCGTGCCTATGCAGATGATCACCACTTTGATGTTGTAACAAATGTGGTTGGA
	TTAGTTGCGGCTGTTCTTGGTGATCAATTTTACTGGTTGATTGACCCTGTTGGTGCTATTTTGCTAGCAATTT
	ACACTATTACAAATTGGTCTGGCACTGTAATGGAGAATGCAGTTTCACTAGTGGGACAATCTGCACCTCCTGA
	AGTTCTGCAGAAGTTGACATATCTTGTCATAATGCATTCTCAAATTAAGCGCATTGACACTGTCCGCGCCTAC
	ACATTTGGCGTTCTATATTTTGTGGAGGTTGACATTGAACTGCCAGAGGATTTACCCTTAAAAGAAGCACATG
	CCATTGGAGAGAGTCTACAGATAAAATTAGAGAAACTTCCAGAAGTTGAGCGGGCATTTGTTCATCTAGACTT
	TGAATGTGATCACAAACCAGAGCACTCGGTTCTTAACAATCTGCCAGACACTAAGCCATAA
	ATGGACAGGAATTCGGGTTCGGATCCCACGTGGCCGCTTCTGGCAAAGCTTGACCCGAACTCCGCCGATAACG
	GCAGCCGGAGAGGCCGACTCAATCGCCGGAACTCAGTTAACTCGCTCAGAGCAGCCTTCCTGTCCAAGCTTCC
	CGAGAAGGTCCGTTCTGGCATTGACTCCGAGGCTCCCTTCGACACTGATCTCTCTTCCACCACCGCCTTAAGC
	AAAGGGGAGAAAGAATATTATGAAAGACAGTTCGCTACTCTAAAATCATTTGAGGAAGTGGAATACATGGTGT
	CACCAGACTATGTTGTTGAGGAGGAAGACAATGAGGAACAGGCTCAACAAGAGAGAGCAATGAAGATATCTAA
	TTATGCAAATATAGCTTTGTTGATATTAAAGATCTATGCTACGGTTAGGAGTGGGTCAATAGCTATTGCAGCA
	TCAACTTTGGATTCTCTGCTTGATCTCATGGCTGGGGGCATACTTTGGTTCACTCACGTTGCAATGAAGAACA
	TAAATATCTACCAATACCCAATTGGAAAGCTGAGGGTGCAGCCAGTGGGCATAATTATCTTTGCTGCCGTCAT
CrMTP8.1	GGCAACACTTGGCTTTCAAGTATTAATCACGGCTGCTGAACAACTAATAGAAAACAGTCCCGCTGAAAAGATG
	ACTGCTGAACAACTAATATGGTTGTACTCTATTATGTTATTTGCAACGGTGGTGAAGTTTGTGCTCTGGCTTT
	ACTGTAGAAGCTCAGGAAACCAGATTGTCCGTGCCTACGCAGATGATCACCGTTTTGATGTTATAACAAACGT
	AGTTGGATTAGTTGCTGCTGTTCTCGGTGATAAATATTACTGGTGGATTGATCCGGTTGGAGCTATTTTGCTT
	GCAATTTACACTATCACAAATTGGTCCCACACTGTCATGGAAAATGCAGTTTCATTAGTTGGACAATCTGCAC
	CTCCTGAAGTCTTGCAGAAGCTGACATATCTTGTTATAAGGCACCCTCAAGTTAAGCGCATTGACACTGTCCG
	TGCATACACATTTGGTGTTCTATATTTTGTGGAGGTTGACATTGAACTGCCAGAAGATTTACCCTTAAAAGAA
	GCACACGCCATAGGAGAGACCCTACAGATAAAGCTTGAGAAGCTCCCAGACGTTGAGCGGGCATTTGTTCATC
	TAGACTTCGAATGTGATCACAAACCAGAGCACTCAGTTCTTAGCAAACTGCCCAACAATTAG
	ATGGTAATGTCGGCGGGTACCAGCGGACACGGCGGCGCCATCGCCGAGACCGGCAGAAGGGAACAACTCCTGG
CrMTP9	TGGAAAACAGTACCGGTGACACGTCGTCGTTTTCGTGGAGACTCAATCTGAAGGAGTTCCAATTGCCACAGCA
	AACTCATAATAACCATAATGATCGTCACCATCATCATCCTCTTTCCTTCACTTTCCATGGCCTTCTTCGCAAA

	CCCAAGAAGCAACGCAAGGTTGCAGAATACTACAAAAAACAGGAGAGGCTCCTTGAAGGATATAATGACATGG<br>ATACTATGACTGAAACGGGTTATTTTCCAGGAAGTCTCACTGAGGATGAAACGAAGCAACTAGCAAAAAGTGA<br>GAGGTTGGCAATTAATGTGTCAAATGCGGCTAACTTGGTGCTATTTGCGGCAAAGGTTTATGCTTCAATTGAA<br>AGCAAATCATTGGCGGTCATTGCTTCAACCTTAGATTCTCTTTTGGATCTGTTGTCAGGGTTCATATTGTGGT<br>TCACAGCCAATGCCATGAGAAACCCAAACCATTATCACTATCCCATTGGAAAGAAACGGATGCAACCAGTGGG<br>TATCATTGTTTTTGCATCAGTGATGGCAACCTTGGGATTGCAGATTTTGATAGAGTCTGGGCGGCAACTGATT<br>TCAAAGTCTAAGCCTGAAATGGATCTTGATGAGTTGAAATGGATGATAGGGATTATGGTATCTGTCACTGTAG<br>TAAAGTTCATTCTCATGGTTTATTGTCGAAGGTTTAAGAATGAAATTATCAGAGCCTATGCACAAGATCATTT<br>TTTTGATGTCATTACTAATTCAGTTGGATTAGCTGCTGCTGTCCTTGCTGTCAAGTGCTCTTGGTGGATTGAT<br>CCTATAGGAGCTATTATTATAGCAGTGTATACAATAAATACATGGGCGAAGACAGTGATTGAGAATGTATGGT<br>CACTTATTGGAAGGACAGCGCCACCAGATTTTCTAGGAAAGTTGACATATTTGATATGGAATCACCATGAAGA<br>GGTTAGGCACATAGACACCGTAAGAGCATACACATTTGGTGCTCATTACTTTGTTGAAGTCGATATAGTGTTG<br>CCAGAGGACATGCCTCTCAATCAAGCACACAACATTGGTGAGACACTGCAAGAGAAGCTGGAGCAGCTTCCTC<br>AAGTTGAAAGAGCCTTTGTGCACATTGATTTTGAATTTACTCACAGGCCTGAGCATAAGATGATGGTATAA
CrMTP9.1	ATGGCAGAGAGTGGCGGCGGACCCCGCCGTAGGGAACCGCTTCTGGTGTCGCCGGAGGAAGAGGCTGCAAACC<br>CTTCGTGGAGACTCAATGTGAAAGAGTTTCGTTTGCCAACCCAAACTAATGATCAGCAACATCACCATTCCTT<br>CACTTTCCGTGGCCTTCTTCGCAAACCCAGGAAGCAACGCAAGGTGGCGGAATATTACAAGAAACAAGAGAGA<br>CTCCTTGAAGGATTTAGTGAGATGGAGACTATGACTGAAACGGGTGATTTCCCAGGAAGTCTCACCGAGGATG<br>AAATGAAGCAACTAGCTAAGAGTGAAAGGATGGCTGTTCATGTGTCAAATGTATGTAACTTGGTGCTGTTCGC<br>AGCAAAGGTTTATGCCTCAATTGCGAGCAGATCGTTAGCAGTCATTGCCTCAACTATGGATTCTCTCTTGGAT<br>CTCTTGTCAGGGTTCATATTGTGGTTTACTGCCCATGCCATGAAAACTCCAAACCATTATAACTATCCAATTG<br>GAAAGAAACGCATGCAGCCAGTGGGTATCATTGTTTTTGCATCTGTGATGGCAACATTGGGGTTGCAGATTTT<br>GATTGAGTCTGCCCGGGAACTTATATCCAAGTCTAAGCCTGATATGGATCCAGTAAAGCTACACTGGATGATT<br>GGGATTATGGTATTTGTGACAGTAGTGAAGTTTGTTCTTATGATTTACTGTCGAAGATTTAAAAACGAAATCG<br>TTAGAGCATATGCACAAGATCACTTCTTTGATGTCATTACTAATTCAGTTGGATTAGCTGCTGCTGTGCTTGC<br>TGTCAAGTTCTATTGGTGGATTGATCCAACAGGAGCTATTATTATAGCATTATACACAATCAATACATGGGCT<br>AAGACAGTGATTGAGAATGTTTGGTCACTGATTGGAAGGACAGCACCACCTGATTTTCTGGCCAAGTTAACTT<br>ACCTTATATGGAACCATCATGAGCAGATTAAGCACATAGATACTGTTAGAGCATACACCTTTGGTGCACATTA<br>CTTTGTGGAAGTTGACATTGTGTTACCAGAAGACATGCTTCTCCACCAAGCACACAACATTGGTGAGACACTC<br>CAAGAGAAGCTGGAGCAACTTCCAGAAGTTGAAAGGGCTTTTGTGCACATTGATTTTGAGTTCACTCACAGAC<br>CAGAGCATAAGACCATGGTATGA
CrMTP10	ATGGGCACTGAACTTAGAACTGATTCCTCTGATTACAGGACAGAGCTTCTTTCCCCAACAATAGCTGCAGAGA<br>ATGTGTCCATGGCAAGGCAACCTTCATGGAGGATCAACATGGATGAACATCGTTTGCCAGAAAGGCAAATGGA<br>GTCTCATTTTGGCTTTGGATTCTTCCTAAGGACACTAAAGAGACAAAGGAAACTTGCACAGTATTACAAGAGA<br>CAAGAAGGGCTTCTTAAAGGGTATCAAGAAGTGGACTCGTATCATGATCTGGGCATGTTACCAGGAAATTTAA<br>CAGAGGATGAGATGAAGGAGCTTGAAAGGAATGAGAAGGTGGCAATTTATGCATCTAACATAGGGAACATGGT<br>GCTGTTTGTAGCAAAAGTGTATGCTTCCATTCAGAGTAGATCACTGGCTGTAATTGCATCAACTTTGGACTCA<br>CTCTTGGATCTCTTGTCAGGATTTATACTTTGGTTCACTGCTTATGCTATGAGCAAACCAAATCATTATCAAT<br>ATCCAATTGGCAAGAACCGGATGCAACCTGTGGGGATAGTTGTATTCGCATCAGTCATGGCAACTCTCGGATT<br>ACAAATATTGTTTGAATCAGCCAGGGAAATCATCACAAAGGCTCAACCTGAGAGGGATCCAGTGAAAGAAAAG<br>TGGATGATAGGGATAATGGTGACTGCAACTTTGGTAAAAGTAGTGCTAATGACTTATTGTCGCAGGTTCAAGA<br>ATGAAATAGTGAGGGCCTATGCTCAGGACCATTTCTTTGATGTCATAACAAATTCAATTGGTTTAGCCACTGC<br>AGTTCTAGCCATCAAATTCTACTGGTGGCTTGATCCTGTTGGGGCCATCCTGATAGCTTTATATACAATCAGT

	AACTGGGCAAAAACAGTGATGGAGAATGTATGGTCATTGATTGGTAAAACAGCCCCACCAGAGTACCTTGCAA<br>AGCTAACATACCTTTGTTGGAACCATCACAAGGAGATCAAGCACATTGATACAGTGAGAGCCTACACATTTGG<br>GTCCAATTACTTTGTGGAGGTAGACATTGTGGTTTCAGAAGAAATGTCACTTAATGAAGCACATGACATTGGA<br>GAGACACTTCAAGATAAGCTTGAAAAACTCCCTGAAATTGAGAGAGCTTTTGTTCACATTGATTTGAATACCA<br>CTCATAAGCTGGAGCACAAGTCAAATTAA
CrMTP11	ATGTTGGAGCCGGTGGAGCTTCGTAGCGAGGAGCAGCGCTCGTTGCTTTTAGATTCCAACAATGGTGACCGGT<br>CTTGGCGGTTGAACTTCGACGGATTTCAGATATCTTCGGAGCACACAGAGAAGCAAGTGAAACCCCCTCGTGG<br>CCTCCATGACTGCTATGGGGTTCTAGGTCAAGAAGATAATATTGCCGAGTACTATCAGCAGCAGGTTGAAGTG<br>CTTGAGGGCTTTAATGAAATGGATGTCTTAGCTGAACGCGGTTTTGTTCCTGGAATGTCAAAGGAGGAGCGAG<br>ACAAATTGGCGAGAAATGAGACATTTGCCATCAGATTGTCAAACGTAGCAAACATGGTTCTGTTTGCTGCCAA<br>AGTTTATGCATCAGTCCGAAGTGGTTCCCTAGCCATCATTGCATCCACCTTAGACTCGCTTCTTGACCTCCTT<br>TCTGGATTCATCCTCTGGTTCACTGCATTTTCCATGCAAACACCAAACCCATATCAATACCCTATTGGAAAGA<br>AACGGATGCAACCATTGGGAATCCTTGTTTTTGCCTCTGTCATGGCAACACTGGGACTGCAAATAATTTTGGA<br>GTCTATCCGCACATTAATATCCTCTGATAATGCATTCAGCTTGACCAGGGAGCAAGAGCAATGGGTTGTGGGT<br>ATCATGCTTTCAGTGACTTTGGTGAAATTCTTGCTAATGATTTATTGCCGCTCTTTTACAAATGAGATTATTA<br>AGGCCTATGCCCAGGATCACTTTTTTGATGTGATAACTAATGTCATTGGCCTTATTGCTGCACTTTTGGCAAA<br>TTATTTTGATGATTGGATGGATCCTGTTGGTGCTATCATTCTGGCTTTGTACACTATTCGCACATGGTCCATG<br>ACAGTGTTGGAAAATGTGAATTCACTGGTTGGAAGATCAGCTGCTCCGGAGTATCTTCAGAAACTTACATACT<br>TATGCTGGAACCACCACAAGGCCATAAGGCACATTGATACTGTTCGGGCATACACATTTGGGTCTCACTACTT<br>TGTTGAGGTTGATATTGTCCTGCCAGCTGATATGCCTTTGCAAGAGGCTCATGATATTGGCGAATCATTGCAA<br>GAGAAGCTTGAGCTTTTGCCCGAGATTGAGCGTGCTTTTGTTCATCTTGATTATGAGTTCAGTCATAAACCTG<br>AGCATGCTCAAGCACACTCCTAG
CrMTP12	ATGGCCGATCAGCCTCACCACCACCACCATCGACCGCACCGCCTCTCCGTGCCACCGCGCACCGCCACTTTGG<br>CGACCACCCCGAGACCCTATCCCACAGTTCGATACTCAACATCAGCATCAACACCCACTGCAACCCCGTCCAA<br>ACACCGTCGTCTTTCTTCTTCTTTATCCTTCTCTAATTCCAAATCCAAATCCAAATCTTCACTCTCATCGCTT<br>TTCCTCCTTCTCTTCTCTCTTCGCTCTCTCTATTCTCTCCTTCCCTTCCTTCGCTCTTCCCCTTCTTTTTCTC<br>TATTCCCTTTCACTTTTCTCGTTTCCCTTCTCTCCTTCTTCCTTACCCTCTCTTTTTCACTATTCTCTTCATC<br>TTCTTCCTCCAAAGATCCTTTTCAACAGCACAAACCTAAGCAGTCTCTTCTAACGGTCACACACTCTCAGCAG<br>AGGATCCTGGTTGCTAAATCAATTGTTCTGGCGCTTGTTTTTCTCCTTCGGTTCCAAGCTCTCCGCTACTGTG<br>GCACCGCCTCAATGATCCTTGCCGAGTTGATGGGAAGCATAGCTGCTCAACCTAAAAGTCAGAACCGGACTAA<br>GGTTCGTGGGTTCGTGTCCTTGTTTCTAGGATTGTTTATGTTGTCTTTTGGTTGGGATCGAATTGAATGTTTT<br>CCCGTTTGGGGATATGCGAAATGTGTGAGGGTTTGGCCCTTGTTGCTTCCTTTTATGTCTGGATTTCTGGCTT<br>GTTATGAACGTGTTTCCATCATGAATTGGGGAACGATTAAGCAGCTTGGTCATAAACGGGTTCGCCTAATTAC<br>CCTGTTTTTCACTACTATTGTGCTATTTGTTCCTGCTGTGATTAGTTTTTTCGTGCTTGAATCTGAAGAAGAT<br>AGTGTTTCATTTGGAAATTTGGTGTGGCCTCTGGCCAATACTGTTGTTTTCGGTGTGCTTTTGAGTGAGAATT<br>ACAGCGATGATTATGTGATGAGTTTCAAAGACTCTAAGAGGGAGTTTTTGGTCACTTTTGTCTGCACTATGGT<br>ATTGGAACTTTTCTATTACCCTGAACTTTCCCTTTGGGGTTTGTTGCTTTGTGGTATATTGCTCTATTTTGCC<br>GTTAGAGACTTGGATCCTTTCCATTCCAATGAGTTTGGCTCGGGAGAGGAATTATTTAGTGACATGATTATGA<br>GGCCTATTAGACATGTTTTGGGTGAGAGGAAGTCCCGGAAAATTGCACTGTTTCTTTTGATCAACACTGGCTA<br>TATGGTTGTGGAATTCGCTGTTGGATTTATGAGCAATAGTCTTGGGCTGATATCTGATGCATGCCACATGTTG<br>TTTGATTGTGCTGCTTTGGCCATTGGGCTGTATGCTTCATATATATCCCGTTTGCCTGCAAATAATCACTATA<br>ACTATGGAAGAGGAAGATTTGAGGTTCTATCAGGATATGTTAATGCTGTTTTTCTGGTTCTTGTGGGTGCACT<br>GATAGTGGTGGAGTCCTTTGAGAGGATATTGGATCCTCAAGAAATATCTACTAGTAGTTTGTTAGTCGTCTCT

	ATAGGAGGGCTTGTTGTCAATGTCATTGGCTTGATATTCTTTCACGATGAGCATCATCATGCCCATGGATTGT<br>CTGGATCATGCTCACATTCTCACGCACACACAGAGTTCCATAGTCACCACTCCCACCATCATCATGATCTGCA<br>ATGCCACAGTAAACATCATCATGATGAAAGACATGGAAGCAATCAAGAATTTATTTCTGTTTCCAGTGATTGC<br>CAAGATAATTCATGCTCTGGTGATCCTGGTCATCACAACCACTCTAGTCATGGGAGGAAAGCTGAGTTTCATG<br>CAGACAATCACTGCAGTAAGAGTTTTACGCATGATCATGCCCAGCTGCATGATTGTCATGAACACTGCAGTCA<br>TGAGAGGATGGTTGAGTGTCATGTAGACAATCACAGCATTAAGAGCATTGAACATCATGGCCATCAGGACCAT<br>CATCATGGCCATCAGGACCATCATCACCATCATGGCCATCAAGACCATCATCACCATCATGGCCATCAGGACC<br>ATCATCACCATCATGGCCATCATGACCACCATGATCATGCCCAGCATCATGAATATCATGATCTTCACAAAGC<br>TCCACTTGTTCACCAAAAGCACAACCATCGCCATATCGATCACAATATGGAAGGATATTCTTGCATGTTCTT<br>GCTGACACGATGGGGAGTGTTGGTGTTGTTATATCTACACTATTAATTAAGTACAAGGATGGCTTGTTGCTG<br>ATCCTGCCTGCTCAATTTTTATTTCAATTTTAATTGTATCCTCCGTGATACCTTTACTCAGAAATTCAGCCGA<br>AATTTTGCTCCAAAGAGTTCCAAGGGTGCATGAGCATGAGCTGAAAGATGTCTTAACTGATGTTTTGAAGATA<br>AGAGGCGTTTATGGTATTCAAAAGTTTCATTTATGGAACTTCACAAACACAGATGTGGTTAGTACACTGCATC<br>TTCATGTGTCAACAGATACTGACAAGACATCCGCGAAGTCCCAGGTTTTGCATCTGTTACATAGTGCTGGAAT<br>CAAGGATTTAACCTTGCAAGTGGAATCTGTTGGATAA
**Genomic DNA (5′ to 3′)**	
CrMTP1	ATGGTTTTTCTCCAACTCAAAATGTTGCATTTTCATGATCTATTGCAGATGGAAGCACAAAGCTCTCAGGATA<br>CCCAAGTTATTGAAATCAGCAGAGATTTTCCTAATGTAGGAAGGAAGGTTTGTGGGGAAGCACCTTGTGGGTT<br>TGCAGATGCTGGATCCATCTCTAAGGATTCTGAAGAACGATCTACATCAATGCGAAAGCTTTTAATGGCAGTG<br>GTCCTATGTGTTATTTTCATGACCATTGAAGTAATTGGTGGCATCAAGGCTAACAGTCTTGCGATATTGACTG<br>ATGCAGCACACTTGCTTTCAGATGTTGCAGCCTTTGCCATCTCCTTATTTTCTTTATGGGCTGCTGGATGGGA<br>AGCTACTCCTCGCCAATCGTATGGATTTTTCAGAATAGAGATTCTTGGTGCTTTGGTTTCTATCCAATTAATA<br>TGGTTGCTTGCTGGAATTCTGGTATATGAAGCCATTGATAGAATCATTGCCGGTCCTAAAAATGTGGATGGCT<br>TTTTAATGTTTTTAGTTGCTGCATTTGGTCTTGTGGTTAACATCATCATGGCTTTGTTATTGGGTCATGATCA<br>TGGCCATGGACATGGACATGATGGGCATGGGCATGGACACGATGGGCATGGGCATGGACATGGACATGGACAT<br>GATGGGCATGGCCACAGTCATGGAATTTTAGTGTCTACTCATCAACATCATGATGCAAAGCATACAAAAGATG<br>AGCACCATCACACCCATGATGATCACATACACCATCATGATGAAAAGCATTCAAACTATGCTCATCATCCTCA<br>CAAAGAGCTTACTGATCCGCTTCTTGGTGAATCAAAAGATGGACGCAAGAAGAAGAAGAAGCAATGGAACATA<br>AATGTACAGGGAGCTTATCTACATGTGCTTGGGGACTCCATCCAGAGTGTTGGGGTAATGATTGGGGGAGGAC<br>TCATATGGTATCATCCTGAATGGCAAATAGTTGATTTAATTTGCACTCTAATATTTTCAGTAATTGTTCTGGG<br>GACAACTATCAATATGCTGCGAAACATTTTGGAAGTCCTGATGGAGAGCACCCCACGCGAGATAGATGCTACT<br>CAGCTTGAAAGGGGGCTATTGGATATGGAAGATGTAGTGGCTGTTCATGAGCTGCACATATGGGCCATTACGG<br>TGGGAAAGATTTTGCTAGCTTGTCATGTTAAAATCAGACGTGAAGCAGATGCAGACATGGTCCTGGACAAGGT<br>TATAGACTATATCAAAAGGGTTTATAACATAAGTCATGTAACTATACAGATAGAGCGCTAG
CrMTP4	ATGAAGCAAATGGAACATGAGAAAGCCCCTATCTTGGAAGCAAGGTCCATGCAGGAAATTGAGATACCCATTG<br>CCTCTGAAAGACCTGATATTCTTCCTATGAAAGCGGAGTCATCTGGAAATTGTCTTTGTCCTTTTTCCAGACA<br>GGAAAACAGTGATGTGGCATCAAAAGAGTCATCAAAATCAGCAAAGAAACTTTCTTGGCTCATTGTTTTCTAT<br>GCTATTGTTATGGTTGTGGAAATTATTGGTGGTATAAAATCCCACAGCCTAGCTGTTATTAGCGATGCAGCAC<br>ACTTGCTTTCTGATATTGCTGGATTTGCTATCTCTCTCTTTGCAGTGTGGGCTTCAGGTTGGGAGGCAACACC<br>GCATCAATCTTTTGGATACAACCGTCTTGAGGTTTTGGGTGCTCTCTTATCCGTGCAGTTAATTTGGTTGATA<br>TCTGGTTTTTTGATATATGAAGCAGTTGGTAGAATTGTTCTCCGAAATGCCAAGGTGAATGGAAAACTAATGT<br>TTGCAATTGCAGCATTTGGGTTTGTTCTAAACGCTATCATGGTTGCGTGGCTTGGTCATGATCATAGTCATCA<br>TCATGGCTGTGGAAACTCGAATCATCATCACAGTCATCATCATGGCTGTGGAAACTCGGGTCATCACCACAGT

CrMTP5	CATCATCATACAGATCATCATCATGGAAATGAGGAACTATCTAAAACAACTGATGAGGAGAACCTTACCCTGG<br>TTTCAAGTAGTCAAAAAAATACTAATGTCTTGAACATAAATCTCCAAGGGGCCTATTTACATGTCATGACTGA<br>TATGATTCAATCTATTGGAGTGATGATTGCTGGAGCCATAATATGGGCTGAACCGGAGTGGTTTATAGTTGAC<br>CTTATATGCACTCTTATATTCTCTGTTTTATCTTTAAGTACTACTATTCCCATGCTTAGGAATATTTATGGCA<br>TTCTGATGGAAAGGACACCAAATGAAATCAGTATCAGTATGCTGGAAAATGGCCTCAGAAGTATCAAGGGAGT<br>GCAGGATGTTCATGACTTGCACGTGTGGGCTATAACCGTTGGAAAAGTTGTTCTATCTTGCCATGTAGTGGCT<br>GAACCTGGTATCAGCTCCATTGATTTACTTGGCACGATTAAGCATTACTGTGAAAAAACATACCAAATACAAC<br>ATGTAACAATACAAATTGAGTAA<br>ATGGAAGCCAGGGAGACTCCGAGTTCCATCAACGGTGATTTTGGGTTCGGTAATAGCGATCGGAGATTCGCGT<br>TTTCGCGCCAAGCTTCATTCCAGCAACAACCTCATACGCCGATTTCCATCGATTCGAACGATTCGGCTTCCAG<br>AAGGCCATTTCTATCCCGAACAGATTCCTGCATAGGCATTCCCTCCGGTGGCCACCACCACCATTACTGGTCT<br>GCGCAAGATGAAAAGCCCTCTGGTTTGCTGCAGAGTTCGTCTTTTTCTTCCTTCGTTTTCTCCGTTTTTCGAA<br>ACATTAGGTCTGGCCATAGGTATATGAAGAGGTTGTTTTTGATGATCTCGCTCAACGTTGCTTACTCCACCGC<br>CGAGTTGCTCATTGGCCTCTTCACTGGCCGTGTGGGTAAGATTCATCGTTGAATTTTTATGATAACGTTGAAA<br>ACCTCTATTATGTGCACATTAAAACTCTGTTTCATTTGTTTTTAGCCTTTAATTTTACAGGTTTATGTACATG<br>TGGTGGAAACTAATTTTACTTTTGAAAATTGTTTACTTAGGATGATTCATCATTTTAGAAAAATCGATTGGGT<br>GAAACCACTCTGTCAACTCAATTTCGATTTGAATCCCTGCTGGTTTCTCATTATTACTTTGCCCTTTTCAGGT<br>TTGGTGTCTGATGCAGTTCACTTGACTTTTGGATGTGGTCTTCTCACGTTTTCGTTGTTTGTTATGGCTGCAT<br>CTAGGAAGAAAGCTGATCGAGAGTATACTTATGGGTGAGATTCTTTTTTGGCTACTTGTTAACAATGCTGCAT<br>TTGTCGTACCTACTTAATTGCATGACACATGTTTATATTGTAACTGAATTTGACGTGTTAAGTATATTGGAGA<br>CTTGGTTGCTTGGATACTTTATCTGGAAATGACCGTATAATTGTCATCATTATTATTATAATTTATTACAATT<br>ATATCATATCAAATTGTAGATTTGATAAGGTATGTCTGAATATGCATGGCAAAGTTCTACATTCTTTAGAAAA<br>CACTATTATTTTTCCCACTTGCTAAAGGTCTCGTGTTAGTTCTTTACTACGTGTCATTGCTAAATTTTGTTAG<br>CAGAAGAACCTTGCTTTGGTTTGTGTCTCTTTTTTGACTTTTTTGCATGAAATTATTCTCCAATGTTTTATCT<br>CGAGAGCTAGTTGTCAATTCTTACATAAGATTAAGGGTGGAATTCGGAACTTCTGTTAAACAAGAAAAAGATG<br>AATTTATTGCATGAGCAAGTGGCAAAACTAACATCTGTAGGAAGTTTTTCTGCAACCTAGTTGCTTCACTTTG<br>TTATACTCGTATTTTAGCCTGAACCCAACACACATTATCCACAGGTCCTTGTGTGCCTGCTTGCATCTCATTT<br>ACTATTGGAGAATTTTTTTTCCAATGTGTGTTTATCTCGTTGCTTGCGTTTTGTATTCAAGAATTTGAATCTG<br>TTCAACAATTACTTAATGCTAGATGCAAGGATGGCTAAACTTGTACATGTTGTACATGTGTGTGATGCTAAGG<br>GTATGCTTAGGAGTGGAGTGAAAGGGAAGAGGGGGACTAACTTGTCATTTCTAATTAATATTCTCTAATTTGA<br>TGTTTTTTTAAAAAATGGAAGATATAAAATGATAGATTCGCATAAACATAATGTCCTAATCTTTTGTTTATTT<br>TTATAAATATTATATAACTCTTAATTTAAAAATAAAAATTTTCCCCTCCCTTTCCTTTGTCTCTAAAATATAT<br>CTTGAAGGTATTTTTTTGAACAACTTTCTACTTTTTGACAATCATTGCAACCCTGTTTATGGTTTTAACTAAT<br>TTGCTTTAATTTCAGGTATAAAAGGCTAGAAGTCTTGTCTGCATTTACAAATGCCGTAAGAATTTCACATTTC<br>CTTCACTACTGCTATTGTTGTTACCTTGTATCATAGCAAATTGGTTGCCTGGTGTTTTTACTAGAACGATGCT<br>TTTATAATTGAACTAATAATCATGTAAAAGTCAATATCCTTTTACCTTCTAAAATGGCATGTGCATCTCTTAA<br>AATCTCAGGTATCCTTGTTCAAGGATTTATCTCTAAAATGCAGAATAGTATTGCATATTAAATATGGCGTAAG<br>ATTTTCAGAGTTGACATGTATAACTGAATTAAGTTGCTGAAAGCTATCTTTTTTACTTTGGTAATGCAGTTTA<br>TTAAAGAAGCAATGATAAATGACATTCTGGTTTTAATTTTATAGTGTTTGTTGTGTCTTTAAAAGGTCACTTG<br>TATGGGATTAAAGAATTTCACATGACATGTTTCAATTTATTTTTTTTGGAAAAATGAGTGTTTCAAGTGAACT<br>GTCCGGCAAACTTATTTCAATCTTTTTCTCCTGTTCATTACATTACATTACTTCATATGCTCTAACACGTTGA<br>ATTTTCTTTTTCTGCTGCTTGTGTAGACAGCTGTTTCTTTTGTTTATGTCATTCTCCCTTGCAGTTGAGGCAC<br>TTCATGCATTCATACAAGATGAATCGGAGCACAAGTAAGTTTGTACTCACAATGATTATCAGTATTTTATTAC

TTCTTAAATATTTTGCATATTCAGCAACAAATAAACGGCCTAGTGTTTTTTATAATAAGAATTGAAATTCTT
GCAAGTCTTAATGTCTTATTTTTGTGGTTGAGTTGAATCAATTTCATGCATGCCAGGGTAGCTACTATAGGTG
TGTTTAGTGTTATGTTGCTGTCTAAATCAAGCATGAATCACTATAGCACTATTTGAACCCTTTGATATTAGAT
CAAGATCAACCTTAAATTATAATTGTCATTTTGTTTTTGGAAAATCATGTGTGGAACAAGTCAGCTATGTTTT
GCCTCTTTGCTTTCTTTACCCATATGTTTTGGATATAATTGTTGATGTCATCCCTTGTTCCATTCTTTTCTCT
GCATTCTTATTTTTCGGTGGAGGGTGGTTTGTGAGGATTTATCATGGTTGACATTGTAATTTATCTACTTTAC
TATCATCAGATTGTTTGTTAATCCTTGTATGATTGCTGACCTCTTCAATTTGTATGTTTATGGTTCAAACTAC
AAGTTATTATTGATAAATAAAGGATAGAAGAATTCCATCTATGTTAGGTCAGTGATGGTATATTTTTGCTGTG
TAATAGTCTATAACTATGTTGATGCAGGATAGCACAAAGCAGAGGATATCACAATTAGGGTTTTCTTGAGGGA
ACCAAGGTTATAATTTAGGGGAATTGATAACTTGGGATTTGTGGAGACATGACTTGTTTTCGTGCATTATGAG
GGTTGTTGATAAACTTAAGAAAGCTAATGATAGAAGTATGAGGATTAAATATGAGGAGCAACTTAAAGAGGCA
TACTTAAGGTTCCTTGGCACTGTATCCAAGAGAAAGAGATGCATTACTGGCTTGCGGCTAGGGTCTTGAAAAT
ATTTTCTTCAATCAATGTCTTCTTTAGGTGGTTTACAATGTCACTTATGTAGGACTAATCAAACTTTACCAAA
TAGTTCACCTTCGCCACTTAAGACCCAAGTGAAATAACCTTTCCTCTAATAAACCTAAATAATGCTTAAATAA
TAATAATAATAATAAATTCTAGAGAAGCCCACAAAACTAATGCTTACAAAACAAAAACCAAATCTTTTCTTTG
GGACATTACTGTAAATGGCTAAAATTTACGATCAGTGACATGTAGATCCTGTGGATTTTCAAAATTACGATTT
CTAGGTTTTAATGCTGCCTTCATCTCATATATGTAAATTTTGTTTCTTTCAGTGTGGGGAGGGGGTTATTTGT
CTCAATTGTGAGTGTTTTTCATTCTAATAATTTGATTCTTGATTTGTTTTTCTTGCTAGTTTTTATTTCTGCT
GAGTGCTGACTGTGTTCTTCGTGTAGGCATTACTTGATTGTTTCTGCAGTCACCAATTTATTTGTGAATCTCA
TTGGTGTATGGTTCTTTAGAAACTATGCTCGGATTAATCTTGGTTAGTATTTTTCTTGGAGGTTTTTAGATTC
ACTTGATTTAACCTCTTCAACATTTTGAATGGGACACTCATGCATTGATAGTTCCTTTTGAGCCTTGATCTGT
GATAATTGTTCATTGTTCCTTTTGGAATTTATCTTGCAGCTTACAGAAATGCAGAAGATATGAATCACCACTC
TGTTTTCCTGCATGTTCTTGCTGATTCCTTTCGCAGGTAGATATGCTTGGATTTACTTATGTTGTGGTGTGTG
TGTTATACATTTCCTTTCCTCTTTACATGCTAATATCAACTTAAATATTTCAATGGTTTCTCAGTGCAGGTTT
GATATTGGCATCCTGGTTATTGTCAATCGGGTAAATACTGTTTCATGTTCTGATAGAGATAGATCTTGAATGC
TTATGTGATAAGGAATCAGAAACAGGCTTAGACTTACTTGGCCTAAATTATTACTGTTTGTGTATTAATTGTG
CATTTAGGCAGTTCTTTAGGTAATTACGGGTTTGAATTGTTATTTTTGTCTTTGAGCAGTTATTTAGGCAGTT
ATAGAAGAGTGTGGGAGTTAGTGCCAAAGTCTTTATATATATGGGGTTTAGGGGTGGTTATTCATTGGGTAGT
TATTCTATTTTCTGAATTCAAAAGGAGACTCATTGTTAGGGGGAAACCCTGGGATTGTCTATGTTCTAGACTA
AAATTCTGACCTAAAAACCAGATTGTTTACTGTAAAAATATGCTGTGTTTAGTCTTTAACTTTTTTGTCTGCA
TAATCATGTCAGAGATGGAAAGTAAAAGCTTAAGGGAAAAAATTGACCATTCCTTAATCCAATTTCTTTAGAC
ATTGTAATTTGGCTTATTTCTTTTCCTTCTTTACAGGGTTCAGAATGCAGAAGTCTTGTGTTTGGGGCTTGTT
TCTGTTACTGTTTTTATGCTTGTCCTACCTCTATTTAGGGCAACTGGTGGCGTCTTGCTCCAAATGGCACCTC
CTAGCATCCCAACTACTGCTTTGAACAAATGCTTGAGACAGGTTATTACTTTTTCTTGGGTACTTGACCCAAG
TAGCTTGGGGATACTAATGCCTAGTTTGAAATTTGTCTGGAGCTCTGACACTTTGTGCTGAAGGATTAGCAGT
ATATTATGCAATGGATTATAAATGACGTAAAAATACTTTTTTCTTTCAATTTTTCTTTGTCTCAAATCAGCTA
AAGGTCCCACTGAATTTATCAAATTTCTTGATAATGCGGATGTTCTACCAATGTTGTATTGAAGCTGTTCTCA
GATTCTAATTTATGTTTTCTTATGTTGTAAAATTGTTGACAAAACTATATAATGCACTTACCTCTGAACACTT
GCATATATACTTGTTATGAAATTCCTGGTTTATTCAACTAAAGGATGTTTTAACGTCAAGCTTTTTTTGGCAA
ATGATATTTTTATCACAGATCTATTATTGGGGGGTGGGGGTGATAGGTGCATGTTGTTCGCTTTAATATATAT
GATTTAGATTTAGCATTTTTAATTTGACATATCTGTTAATGCGTCAATGCTTCCCTTTTTTAACCATATTATA
ATTTATTCAGATTTCTGCTCGGGAAGACGTACTGGAAGTCTCCCAGGCTCGATTTTGGGAATTTGTGCCTGGA
CATGTGGTGGGCTCACTATCAATACAGGTACTTCATTATTTGTTCTGCTTTCCTTTGTGGCTACAGGGATTAC

CrMTP6	CCGTGGGAGCAAAATCTTAGGATACAATATATGTCTGTGATCTGTACACATGACATCCTTAACAAAAAAATGT<br>CTAGATACATGCCTCTCACTTGTGGGCAAGCGAGCACCCGTATGAAATGTTGTTTATCTGTTGTGTAGAGAGA<br>CCTGAATTTCAACTTCAAAAATTGATTTAGCATGACTTGTTTCCTTTTCTTTTTTATTGAGTAGCTAGTTAGT<br>TGCAGTGTAGATTGATCCTGACTTTTAGTGAATGATACTGACAGGTAAAGAAGGGGATGGATGATCGCCCAAT<br>ACTGGAATTTGTGCATGGCATATACCATGATTTGGGTGTGCATGAACTCACTGTGCAGACCGACGATGCATGA<br>ATGGGATTTCGATTGAGAATTCTGAATCCCACATATAGAACATGCATTACAAGGCTTTCCTCTTCAAAATTTG<br>TTCCTCCGGTGTTGGAACCACTCAATTTGTCTTCATTAGAGTCTTGTTTCAATTCCTCTGAAAATCCCGCGTT<br>CAAAATCCCCAAGAGATGGCATTTGGGTCATTCCCATCACGACGACTATGATCATCATCACCACCTCCGTCAG<br>AATCAGAAGGAGGGAGAGAATATTTTCCGGCTCGGACTCGCCGCCGACATTGGTTTGGCCACTGGAAAAGCCA<br>TCACTGGCTATCTTTCCGGAAGCACTGCCATTATCGCTGATGCTGCCCATTCCATATCTGATGTGGTAACTTT<br>CACACTCGCTGCCTCTTCTTTGATTGCTCTCAAAGTGCAACTTGTTGTCACTCGTGGATGCAGCTTTCATAGT<br>TATTGTTTGTGTTGCAGGTTCTTAGTGGCATAGCTTTGTTGTCCTTTAAAGTTGCCAAGGCGCCAAGAGATAA<br>AGAACACCCATATGGTCGCTTGATTTGTATCCACACGCTTCTCCATTTATCTCTTATACATCTAGTTCCTGTT<br>TATTAGATATTCTGGATGCTAACATTTCAGTCATCACTGAGTGTAGGCCAGGAGTTATTTTAAATTAGTTTTG<br>GATAGGTTATTTGTTATTTTACATATAATAATAATAATAATAATAATAATAATAATAATAATAATAATCACAT<br>AATGCCATTCTCCCCTGTTATTTTGTTATGATTACTCGTTAGTCTAATGCTTGCCTTTAGTGTGTTATTTACT<br>AAAAGGTCTCTGTGTCATTTATGATTATATTTGTCTGCCATTGTATCTCAGGACATGGTAAATTTGAGACTCT<br>AGGGGCTCTTGGAATCTCTTGCATGCTATTGGCGACTGGGGGTGGCATTGCGTGGCATGCTGTAGACATTTTG<br>ATGGTATTGAAATTTAATCTTCAATCTATTTGCTGCTTCACTTATTCCCACGAACAGCATAATTTAAAGAAAT<br>TTTGATTGAGCATTTTGTCAGACTATGCATGAAAAGTGATACCTTGTCATCTTTTTAATATTTTGAAGTCATT<br>GTTATACACATGCAGGGATTGTCCTCCGCTGGTCCAGAAATGGTTAGCCAGGCATTGACACATGAGCATGTGC<br>ATAGTGACCAACATGGTGGACATCACCATGGAATTGACATGGATCATCCTATCCTTGCTCTGAATATGACTAT<br>AGTGTCAATATGTGCTAAAGAAGGGTACTGCCATTACTTAATATTTACATGAACTCTGGCGAGGGGTTCCTAC<br>TATTCCTGACGTCTATTTCACCTATGCCGTGTCACTATTTTAGATTTGTTGAGTTTGATTAATTGGTCATTGG<br>GTGGTATCTGATGTTCCCCCCCACTCCACTCACCCATGAGTTTACAATACAAATCATTATAGTTTGATTCAAT<br>CTCAAGTCAGTAAGACAAGTATAAAAGGACAATCACTAAATTAGGAGACCTATAGAGAACAAAATTTAATTGA<br>AAAAATTAAATTAAGTTACTTTTGTAGTTATGTTAGCCCAAGCACTTTTGTTTCCTAGGGAATGCATTGCCAT<br>GGCCTTAACTACTTGATGTTTCCTCATTTGACAAGGTCTAAATCGAATCTCTTAACTTTTGGTTGGGCTTGCA<br>GCGCCTGAACCTCACAATTTGAGCTCCTTTCCTTTGTTACAGTCTCTTGTTTATGGTCTTTGTTTTGGTTAAT<br>ATTTTGCACCATTTGGTTGTGTTTTATTTTATTTTATGGGCAGGTAGTGTATTGTTTCTTCCTTGAGTTCAGT<br>ATCTATTTTGTGTTTTATTTCTTTTTTGGGGAGGTAGTGTATTATTTCTTCTTTCAGTTCAGTATCTATATTG<br>CTTTGTAAATCACTTTGTAGACTTTACTGGATAACAAAACGAGCTGGTGAGAAGCAAGGTAGTGGACTAATGA<br>AAGCAAATGCATGGCATCACCGTGCAGATGCAATTTCATCACTTGTTGCTCTCATTGGAGTTGGTAAGGAACT<br>TTGTTCTTTGAAAGGTTACTTGGTCTGTTATTCTGTATAATGTCTTATAATGCTGTTCTCTATTACAAGTTAT<br>GTCATCAATCAGGTTTACTTGGTTACAAGGTATTAACAGGTGTGCTCTTGGGCTATGATTATTGACTAATGAT<br>CAATTTTCACATTGTATTAATCTAAATTGTTCTAAGTCTTTTCATAACATTATTTTTATATTTTATTAATAAA<br>GTGGTTATTTGTACATCAATCGCCAATGGTATAATTGATACGAAAAAAAAGAGTGGCCCCGTGTATGAGGCTC<br>CCACATAGTAGTGTCTAGGGAGGATAGGTATATGCAACCTTATCCCAATAACTGGACATGTTTTTGGATTTGA<br>ACCCATGACCTCCAAGTCACAAGGCAGTAACCTTACCGTTGTGCCTATGGCATATTTGATGTTTGACAATTTT<br>ATGTGTTTTGATGTATGGATCCATGAGCTGCTTCTGTTTATTTGTTTATTGGTACACTATTAATGGATTTTGT<br>AATATTGTTGTTGTTAGAATAACTTAAATGTATATAACCCCTTAAATTTTGTTGCCTTTTGCAAATCGGCACC<br>TTGAACTTTCATTTCATCCTGATTAGTGCTTAAATTCCTTTTATTGTTACTATAGATCCTTCTGTTCACTGTT<br>CGTAGGAGTTTAAGGATTAGTTGAGATACAGCATGAAAGCTCAAGATGTCTATTTGCAGAAGACACAAAGTTT

AAGGTGGTTATATACTCTTTTCCTAATAAAAACAAAATCCCATCCCTGCTTATTGTTAGTAACTTAACCCTAA
GTTAACAGAAGAATCTTTTTCAGCATTAATAAGAGTTTAACGATTAATTGGGATGAAGTGAAAGTTCAAGATG
TCTATTTGGAAAAGGCAAAGTTTTTTTTTTTAGTAGTATGCTCTTTCCCTTGTTATTATTGTTATCATAACCT
AAATTGACATAACTAAAATCATGGTCACTAACCTTATAGTGAATGAAGTCTGGATCTGAGCTCAAGTTTTCTT
ATGTAGACTTGATGGGTCTCAACAGGATGGGCCTACAATTAAGGAAGTCTACTTTAGAAGGAGAAGGACAAGT
ATGGACAATTTGAGAAAAAGAGGGGATGAGTTAGTTATGCAGAACTATAACCATAATGACAAGCGGCAGCAAC
AGATTGGGGTTAAAGGATGCAGGAATCATGGATATGGGGGGAACAAGGTAGTGGAGGGGGTGATCATTGTGGG
AAAACTTGGGGGGTTGGCTGGTGCCATAGGGCAGAGAATTCTCCTTCTCTATTGTTTTCTTTCTTTTGTTACT
AGTTGTTTCCCAATCCTGCTACACTTTTGCTGTAGCCAGAACTTGCTCTCTAGAATCTTTTCAATTAATTCTA
TATTGCTCCTACCAACACTCATTTGATTTAGTTGAATTGACCAGGTACTGCTATTTTGTTTAACATTAGAACA
TACTTAAAAGACAAACATATAACAAATACTGTGTTTACAGTCTACCATCCATTGCTGATATTTCAGTTATTTA
ATTACTGAATTTGAGTCTTTTTCTAATTTCTACTGTACATTGTCACAGTACTGTCATGTTAACAACTATCTGG
TATTGTATAATCCTTTACAATCCAAGACCTTGGGGGACATTTGAGTTTGAGGGATTACATTGATAAAAATAAT
TCGTAAGGGAAACTGGATATATCTTTCAAGGATTATAAATTGCTTAACTCTTTTTTGGATCTTACATAATGAC
TGCAGGCTGAAAGACATTAAACCTCTAAATTAATGTACTCTCTGTGTTCCACAGGCTTTGTTTCTGGACTCTT
GAACCATTTATATCTTGTGTCATGCATACATTGACTACATTCACTAACCATAAATCATGTTAGGAGGGTCTAT
TCTTGGAGTGAGATTTCTAGATCCACTTGCTGGACTTCTTGTCTCAGGCATGATTTTGAAAGCTGGAGCAGAA
ACTGGTTACCAAAGGTACCTTATTCATTGCTATCTGTTTATCCACTATTTATCATTGCTTGCAACTGGCAAGT
TTAACTGATTGTTTTATATGTTACTAAAACTGTTTCCTTTACCCCACCCCAATGTATTCATGTCTAGCACATT
TTGGAATCTTAAGTTAAACATATAGGAACCTCATATGATCCTCTGATGCAGATAGAACAAAATATCGAACACA
CTTATGTTGGATAGATTTGTGCTGGATACCTATAAACTAGATTGTATATATCTCATAATTGAAAGGTTACATA
TATCAATATGGAATCTAAATGTAATGAGTCTACTTGTTTTGTTTTTCCTTCAACTTAGAGGGACTTAATATAT
GTAAAACATTCTTGTTTGACAAACAGCATTGTCCGTTTGGATGGATCTTTTAGTGATGCACGCATGACATATG
TTAATATGTAAATTGATGGGTTGTTTATTTTCTGCATATTAAATATAATGGCTAATGTTATCATTTACTAATT
TTAGTCAATGAGTTTACTACCACTTTTTCCATGCTTAATGAAAATGCCACCTGGCATGTTATTAAGACAACGG
AGAATACAAATGGTTGCAGAAATGACTCCAAATGTGTAACCCAGGGGGTAAACTGATTATTTCTTGCCTAAAT
CAAGGGGGTTGGAAAAACTTTGTCCAAACTTCTACCTCTAATTTTTTTGTTGCTTTTGATCAATTATCAGTAT
CTTGGAATTGGTGGATGCTGCAATCCCAGCACAGCATTTGGATCCTATTAAACAAACAATAATGCAAGTTGAT
GGTGTCAAGGTGTTCTTTTTTCCAATTTTCTTTTTAAAATATCATTTTTGCAAAAACTTGGACCTTGCATATT
TTCCAATGTCATCCTTTTACTTGGGACAATGTATCAAGTTCTGCTTCAATTTTTTGCATGCACTTTTAACTAG
GTGTTCGTTTTTGCTGTTTTGCATATGAATATATGATCTTAGATTATATTCTTTGCAATTCATTGCTTTATTT
TATTTTTTTCCAATTCCTATACAGGGCTGTCATCGCTTAAGAGGAAGGAGAGCTGGTTCATATTTGTATCTTG
ATGTAAATATTGAGGTTAGTTCTGAATTTTATATTCCTTATGTCATAACTCACATGCATTATATGTCAGTTTT
CAGAATTTACTTGTTAGTTTTATCTTGCAGGTTGATCCTTTTTCCAGTGTTAGTGCTGCACATGACATTGGTG
AATATGTTCGTCATCAAATTCACAAGTCGCATCCTACCGTGACTGAAATTTTTATACACATAGGTTTGTTGTG
TAGTTTTATAAAGCTCTTATCTGGTTAGTGAAATTTTTATACACATAGGTTTGTTGTGTAGTTTTATAAAGCT
CTTATCTGGTTAGTACATAATCTTAGTTTTTTGTTTTTCAACTTTTTTTTTAAAAAAAGACTGAAATTGTTAT
CAACTGAAGTTACTCTGTAGCAAGTTTATCCAATTGTGATTCAAATAGTCAATCACTAAAGTTAATTTTTAAA
TCATGAATTGTAAATTGTATCAAATAATAAGTTTTAGAGACACAGTGAAAAATAGCTTGAATTGTATCATACA
TGTTAGAAAAATAGAATATAGAAATGGTGGAATATGTAAGCTAGAAACAGTTCCTTAATTGTAGGGATTCAGT
CATGGCTCTTAATTTGTAATTATTGTAAAGGTACTATAAATACTACATGTGTGGTTTGCTTTAAACACACAAT
ACATTCAGCAATCACTTCTCTTATAATAAAAGTAATGTTTAGTGTTCGATATTATTATTTTAAAAGTAGGAAT
GACAAAAGTACTGAACCATAAGTCTCAAATAACATCATAAGAGTCATGTGTCATATGTCAGATAAAAAACAAC

CrMTP7	GTAATGATATAGCCAAATAAAGTGTGCATAAACTGTGCATGTAACTATGTTAAGCCTCTGTTTTGAAAAAAAT<br>GAAATGAACAATGGAAAAACCAAATAAAGAGCATAGAGTGAGGGGGCGACTGTGTCAAGGCATGTTTTGGAAG<br>AATGAGAGCTGAGCAATGGAATAATCAAATAGGATGTGTTCCATAGCATTCCTTCAGTTCCTTTTCATTCAAC<br>AATTTAAGCATATTTGACTTTTCAATTTGTTTTATGAGAATTGGTGAAAATTAGAAGAAAAAAAAAATCATTG<br>GATTGTGCAATTCATATTAGCGATACAGGATGTCACCTATTCATGCATGAACTGCATGATTGAAAACCATTTG<br>TGATTCATCACATAATAAGAATCACCGACTTTCTAAATTCTGTCATATCATGAATCATGCTATTCTAACAATT<br>ATGCTTTTTAGTTTTTCGTTCAGCACATTTTCCTTTTCTTACAATGTGCTTGCCTTATGCCCACAATCACATC<br>TAAGTAATGTGCTTACTTAATATTGTGTCTACGTTGACTAATATACAGATAGATGGCTTTTGTGCCTGCATGA<br>GTAAAATACTATTCCACAACAATAAACTTTGCAAGAGTTGTATTTGTTTTGGCGTTAAATTCTACAACTGCTA<br>TTTGCTTTTCTGGCGGCCTAGTTTTTTCCCTATGGAGCATCAAATATAAGTTTTGCATGATACACGTGAAATC<br>TGATATGGGATGGACATACCTGACTATTTATGCATTCTAGTATATAACCGAGTCTTCTGAAATTTATTCTGTC<br>CATACCAGTAACCTTTTTCTTACTACTTTTGGACCATTACTAATGTAGAGAAAATGATTAAACTTGTTTTGAG<br>TGGCAAAAGGAAGATTTGACTCTGTCATCACATTGGTCCATCTCTCTTTAAGTCGTATTTCACAGATTAGAGG<br>TCTGTGAGGGCAAAGATAATGCCCAATTGTATATTTAGGCAGCCTTTACTATCAAATTGCATCCTTATGACAA<br>TTAAATTCACTTATTCCCAATGATATACTGTAATGAGGTTTTCTAAAATCTAAATCATTCTTCATAGTGGATA<br>TCATGTAAGCTGCTATTTGTTTCTTTTAACTAACATTTTTTTCTTCTTTTCTTAATTCTCTTGTAGATCCTGC<br>AATGTCACATGTTTCTCCCGGCACAACAGATCAGCCAGATAGTTGGAGCCGAGACATGGACCAGAACGGTGTT<br>GTTCCAGCTGGGGATAGTAACATTGAAGGAATTGTTTCTGATATCATCTCATCAAACTTTCCACAGGTAACAT<br>CTTTATTGCAAACCAAAAGACAATCAAAATAAGCTTCAATTTGGAATTTGATAGGGTCATTCATCTGTTGGGG<br>ATTCTTTGATTTTATTGGTTATAAGTGATCCATGGTAATGGTAGTACAATTGAATTATAAAGTTAGACTAGAA<br>CTACTTGTGTTTTCAACCTTTAATTGTCTCTAGCTGGTTTTCAGATGTCAGTTGAGCGCATAACACGGCACAT<br>GTTTCAAAGCAAGATTGTTCTTCAAATTGAGGTTTCCATGCCACCTGATATGCCAATTCGGTAAGTTTTATTG<br>TCTAACCTAGTGAGTTTTTGTGTATTGGGTCAGTCCCTCGTGGCAATTTATGTTTATTTATCCGCAGACATGC<br>AATGAAAATGGCAGAGCAAGCAGAAAAAGAGATTTTGAAGGCTGTCTCAAATATAATGCATGTTGGCATTCAG<br>CTACGTTTGGGGCAACCATTCCCACAGATAAATGATACTTAG<br>ATGCGAACATCCTATTCCTACAATCTTCTTCGCTCTCGCCTCCGCCACTCTCACCACCAACACTTGTATTGCG<br>TTCTACTTGTCAAACCAGTAGACCACCACCTACCATCTCCCGCCACCACCTCTTCCACTTTCTATTCACCCTT<br>CCTCTTCCCACGTTCCTTCAATGCTCATCGCCACTCATTTCATCGCAGTATGTCTCTTCTCTCTTTTACTATT<br>TTCTCATTCATTCATTCACCTCTTTTCCTTCTCTCTTCCGTTCTAGGTTTCTTTACAAGGGCCAAGCCAGCTA<br>AGAAAATTGAATTCAACGATCGCCACAGGTCCTTTTATTTTCCGATTTGCTTTTGCTTAGCATTATCTTTTTC<br>TCATATTGCTATGGAAATGCTTCTCTTCCACCATTTCTCTTCGGTCTTTCTTTTGTTTATTTTTATTTAATGT<br>GCGAGGATAATGCTGTCGTAACTTGTTCCGTTACCTGATTTCTTCTTGTTTCCTCCCCTTTTGTCCGTTATTC<br>TAGTCAGCGAGCTGTCACAACTGCATTGTGGTGTAACTTTCTTGTTTTTTCTCTCAAGTTTGGAGTATGGTTA<br>GGTTCCTCTAGTCATGTCATGTTAGCTGAAGTGGTGCACTCGGTTGCTGATTTTGCAAACCAGGTATATGAGT<br>TGGTTAGCATTGTACAATACGATTCTTTATGTAAACGAGAAACTAATTTGTGCTGCATAACTTATTTTGTAGG<br>CACTACTTGCATATGGTCTAACTAGCTCAAGGCGTGCACCAGATGCCCTTCATCCGTAAGTTAAACAATGTTG<br>TTGTGTTTGTTCACCCCCAGCCTCCAATTTTTCGTTATGTACTGATTGTTGAGCTGATTTCTGACATTGGATA<br>TGAATTCACGTGTTGTATGATTCAAGAACCATTATACATGTAATGGATGAATGCATGGTTAGCTATTGTGTCT<br>GATTTCCATGAATTGTGCTTCAATCGCTATTTGTGCAAAATCTAACTAGACTCTATATGATAATAAGTTATGG<br>CATCCATTTCTATTTTATGGGATGAACGTAGATGTTGCTTTTGTGTTTACTTGAATTTTGGAACTTAATCACT<br>GTGTTAAAATGATGTCTTCTATTTCTACAGATATGGCTATTCCAAGGAAAGATTTGTCTGGTCTTTGATATCT<br>GCTGTTGGGATATTTTGTCTTGGTTCTGGTGCTACTGTTGTTAATGGAGTTCAAAACTTGTGGACTGCACAGG<br>TATTTAACCAAATTGGATCTTTGAAATTAAAACAGTTAACTTATTCTATAATAAATTATGGTCAATTTTCTTA

	TTTCAATTATTCATCTGTGAATTCTCTTATTCAAGTTATTGACTTTTTAGAGTTTTTCTTTTCAGCCCCCTGA
	AAATATGCAGTATGCAGCTGTGGTGATATGTGGTTCATTCATCATTGAAGGTACTGCTGTCTGCATTTCAGTC
	TGGTGCAATTTATTCTCCTTTGTCTATTTCAGTCCTTTCCTTTTTCAATGATCATTATTTGTTAGGTGCCTCT
	CTTATTGTTGCCATACAAGCTGTCAAGAAAGGTGCAGCCGCGGAGGGAATGAAATTAAGAGATTATATTTGGC
	GTGGCCATGATCCTACATCTGTTGCAGTGATGACAGAGGTAGTAACTTGTGCTTACTCATCTTTAGTTCCTGG
	ACCATGTTACAGTTTATTGGGTTTAAGCAACAATTGTTGATGGATCAAAGGAGAAAAAAACTCATTTAGATAT
	AAGTTTTGGATAAAATGAATTATTTATCACGTGTTACATATTTGGCTATATTTTTCCTTGGAAACAGTTAATT
	AAAGCTAGTTATTTTTTGGATATTTAACTAATGGAACTTCAGCTTCTGTGCTCCCCTGGGCCATGGTAAAGAA
	AATGTGTTTTTGTTGTCATTTTGCATCAGAAAAAGGAACAAGGATGAGTGACCTAAAACTTTTATTGCCGGGA
	GTTTTCTATTTGTCTCTCTAATGGACTATGCAACATAAACTTATTTGTCTATTTAGCTTTTAAACCCTTTTGC
	AACTGATGAATTAAGATTTTGTCTGGTTGTTTGTTCTCATATATGGTAGCTAAATATGCAGGACGGTGCTGCA
	GTAACTGGCCTTGTTATTGCTGGGGCATCATTGGTTGCTGTGAATGTTACTGGAAATGCCATTTATGATCCCA
	TAGGTTCAATCGTAGTTGGCAACTTGCTTGGAATGGTAAAGAGGGTTCAGAAGACAGCAAAATAGCATAACAT
	TGACACCTATTTTAATTTAATTTCATGCTGAACTAACCATGCAGGTAGCCATATTTCTTATCCAGAGGAACCG
	ACATGCTCTGATTGGTAGAGCTATGGATGACCATGATATGGAGAGAGTACTTCAATTTTTGAAAAACGATCCG
	GTACAAGCTTACTAGGTTCTACTGAGATATGATAACTTCTTATCATTCATTTGACTAAGGTGACTTTCATGCT
	TTAGGTTGTAGATTCCCTCTACGATTGCAAAAGTGAAGTTATTGGGCCTGGATTCTTCAGATTTAAGGCAGAA
	ATAGGTTGGTTGTCTTTCTCGTTCATTGTTGTTATTTTCTATGTAATATAGGTTGTGGATATAATTCTGGGAT
	TTGCCTTTATTTAGAACATAGGCCATTTTGTTTGTGTTTTTGTTTTTTTTAAAAAGTGGAACTTTTTAGTTCA
	AATATATGCTTAAAAAACTCGCTATCTTTTTTTTTTTTTGGGAAAAATGATCATGCATGGTCTTGGCAATTATT
	GGCATCAAAGGTCTTTATTTTATCACAATAATCTTCCTGATCCTGTTATATGTAAAATCCTACAATCATTTAG
	TTTGCTATAATTTTCATCACCATTTTTTGGTTATTTGAAACACGCTATCCTTCTCAAGTTTCAATTTACATAG
	CTGTAGCCTGTAGCTGCTGTACTACTTCTGTATTCCTAATTTCCTTTTTGATATCTATTTTTATATCTATTAG
	AATTGGTACTCAGAACCGTAGAACAAGCATATGTTTCGATAAACTTCAATTGGGAAAAAAACTTATGATATAG
	CTTTTTTTTAGAAGCTGGTCTTTCTAATTTATGAGAAAAGCATTAAAACTGTCTTTGAGGATTTTTTTCTTTT
	CCCCTGAAACTTGTCTAATTATCATTTTCTTTAATGAAAAATTGTTTCATTACTTTTTAAATAAAAAGCACAA
	GCAAACTGACTCAAAATTGGAGCTAGAATCTACATAATAAATTCAAATTTTACCATCTACATCCATAAGCATG
	TCAAATTCTGGCAATGGTAACAATTAAACAGGATGCTGTTAATATGATCATTCTTGATGAAACAGATTTTAAT
	GGAGTGATGGTGGTGCAAAATTATCTTAAAAGGACTGGACGTGAAGAGTGGGCCAGACAGGTGAATACTCTTC
	TTTTATTATTGTTCGTGTAAGACCTCTGATAATGTTTTGTTCTGCTGTTATTATTGGTATTGTTCCACTGCTG
	CTGCTTTTCATGACTTGCTAGCGAAACAGTAATGCCTTGACACCAGCTTAAAATATGTGCATTTGTAATTAGA
	CTACATGAAAACTATCCTAGGACATTAGTTCACATTTAAAATAATAGTATAATTTGGCTCTTAAGGCATGGCT
	GGTGTAGGTTACTTCCCCCTTTCATGATCATTCATAATAGTTCCGTTGCCCCTGCTTCATGCCATTGTGTGGT
	TTAGAATATTTAGATATGTAACTTTCATAATGACCAAGTTGATTTTGTACCACTTCTGGACTTTTGTTATTTA
	CAGTTTCGTGAAGCTACAAAGGAGAAGGATGATACGGCCCTAGTGGAGATTATGTCAAATTATGGTATGCAGT
	AGTAAACTTGATCCTTATAACCTTGCTGGATGAGAATGGAATACTTACAGTTGTAAGATGTGGCAGGTGAGGA
	AGTAGTTATAGCTTTAGGAAGTGAAGTGGATAGGTTAGAGAAGGAGATCCAAAATATCGTACCTGGCATTCGG
	CATGTTGATATTGAGGCCCACAATCCAACAGAGGCGACCTTGTCAAGTGAAAGTTCTCAATAA
	ATGGGGAAACAGACAATTCCGGTAAAATCGGTGACTTACGCACTCTCCCCTTTCCAGCAGAAGGTAATGACCG
	GTCTCTGGAAGGATCTACCAACAAAGATTCACCATAAGATCTCCGAAAATTGGATCAGCGCCACTCTGTTGCT
CrMTP8	CAGTCCCCTCGTCGGAGTCTACACGTAACTTTCTTTTTTGCCTCAATTTTATTACATGATTATTATGTGGTTG
	TGGTTGTAAATTCCGTCAGCTTTCGGATATGAATGATTAGAATTGATCTCCTTAGGGTTTTTACCAACATCGA
	AGACCTATTTAATTTTGCTAAGCATCTTATTGTTAATTTCTGGAATATGTCCACTCGTGTGTGCCTTTTTTTT

GTGTATTCTTTTTGTATTCTGATATGAAGTCTGAATCGCTAGGGTAAAGGAAACAGTTTCATTTCAATATACA
CTAAATTAACTATATGATATGCCCTGATGAACTTTGATGAACTTGATGCTTAACTACTTGCCAAGATGCTGTT
AAGTTTGATGCACCTTTCAATGTATTCGTGGCCTCTTTAGGTGTTAGCAGTGTTTTTCTCCATCTAAGAATTT
TGTTTTACTGTGTGATAGGGAACCTTAGTTCCTTCGCTTTATTCTTCATGGCAAAGTTTCTTATTTGAATTGA
GAATGAGAACTTGCCACTCCTATCTTGAAGATATGAAGTATGCAGTATACTAGGCTTGTGTCTGAGGACATCA
TATCTGTCCTCGTGTTTGTGTCAATGAGAGCATGATGTGCTGGTCTGCTGAATGGCTCTGTAGGATGTATCTG
CATATTTATGGATGATTTGGTTATCTTTGTATGTTTTTCTCCCTGTTTTTGGTGGTGTCATTTGAGATTACTG
GCTTTGGTTGTTGCTGAAACCATGGTTGAAGCCAATACCAATTTTACCTGCACCATGCCTCCAAATGTGTGCA
ATATAAGGTTGTCAAGATTGAGATTCTATCATTTCTATGTAGGACTGGAAGGGGAGGGTAAACACATGAAATG
TGAGATTATATCTCAGATTATAATTTTCTATCTAGATTGTAAAATTAAATCTAGATGTGCACACATACAAATA
ATGTAATTAAGCAGAATAACCTTAAATTGTAACTCACATTTAATTAAACTCAAATTATAATCTAATTAAGGTT
ATAATTCATAAAATAAAAGATATATAAAGTTATTTACATGTAAAACTAGAAGACAGCCAAAGCATCCACCAAA
CATAGTCAAGAGTTTGTGGGTACTTTCCTAAAGGTCAAAGATTGATTTTTTTTTTTGTATTTTTGGGTCTTCG
CCCTTTATGTTATTTGAAAAAAAAAAAAGTTCTGGGATGCTTCAACAGAGTCAAAAAGACAGAAAACCAAGCT
CACTTCAATGAAACATCCAGCCACCCTCAAGTCAATGACTCAGATGCAGGCAGCACCTTGATGAAGTCCCATC
CCCATGAGCAACACTGCCTGATTGTTTCTGCCTTCCAAACTCCATTTGGCCAAAGAAATTATTTTACTTTGTG
AAATCATGCATGGTACCATGGTTTTATGCATAGGTAATCATGTAAAGTCAGGTTTGGGGAGTAAAACAGATAA
ACTCAATCTTGAACAAAGGGGAACACCAACAGAGTTGTCGCACCGCTGAGAGTCACAGCACCACTTATGCAGT
GGAACGGTGTCTTAGAGCCTCTAGTGATCAGAGATGTGTGTGTCAGGGGAGGGAGGAGCCAAAAGAGGGGTGG
GGTTGGAAAAAATGGGTTGCTCAGCCCTGCCACTTACATGGTGGCTTGTCCAGATTTATGAAAATTGCCCTAT
TTCAGTGATGTCACAAGCTTGCCTTACAATTTTATAATGTTCTCACCAATTTGAGTCAGTACAAATATTTGGA
TTGAAGCCTCTGCACGGAAAGTAAAGTAAGATTGTGCAATACTTCAAGGAAGATTGCAATTCTTTTTTTTAGA
GTATAGTGGAAGTAATTGGATTTATACGTAGGACCTCATGCAAACTATCTGAACTTCCCACCACTAGGCTGAC
CCTAGTGGGTTGGAAGATTGCAATTTGAATTTTGATCAACATTGGTGCTATATGTTACTATTAACTATGCCAT
TGTGCAAAATTTTGTGGTACATCACCTGCAACCTATGAAGAAAAAATTCGAGAAAGAAAAATAGAGGTGTTGG
GTGGGAATGAATAGTTTCTTTCATTTCATCCTATTTTAAACAATTCAAACAATTATATTAGAGTAACGTCATT
CTATTCCACTGATTTTCCTCTCCTTCTCACTATCCAATATAACCTCAGCAACCATTGTTGTAACTTCTATCTA
CTAAAAGATCAAGGAAAGATGAAAAAATTAAATTTAACTAGATATTTGATCTGGTATTTTTTTTCCTTGAAAC
AAGTATGCTAACTTAGCACTGGTCATTAAGTAAAATAGGGGCCGGAATTGAAATGAAATTAGCTACACTACAG
TAATTCTCTGGAGAGATGGATTTGGAGCAGGACAAATGCTGAATTAATTTATTTATAATATATCCTGCCATGA
ATCTCAAAGTGCATCTCGTGATGAAAAGCTCAACCTCTTGAGTGGTTCATCTCTCCTTAGAATTGCTCATTAG
CTAAGTAGCTTTGACTGAAAAGTGACTCTCTCTTTACGGGTAGTTAAATTGTATGTCTTTCAATGGATGCAGG
TATGTTCAAAACTACCAGGAGAAGGAGAAGTTGGCTCACAGGTACTGAGCTCATGCAAGAGCTATTGGATAAC
CTTCAAGCTTGTTGACTTGCTTTTGGTCAGTCAGCCAATAATGTGATTATTGAAACTCTCTTACCCTTTGGTT
TTGAGATGTCCGAGTTTGAGACTTTTCAACTTCGACAATGATTATTGATAGAATTTGTGTTGCCAATAATGCC
AAAATTGGTTTGCCATGAATACCAAAATGAGTTGGCACGTTAAACATCAGGATGATTGAATCACTTTTATCAT
TTTTTTTTATAATTCGATTCAAACATGCTTTCACAAATAATCACTGAATTTTGACTAATTTTATCAGCTGTTT
ATCTCTGAAAAAAATAGCATAACTCTATCAATATGAGCCTTTTCAACGTAATATTTTCCCAAATTTTGCTTAT
AAAATTAATAATTGAATTCTCATTTATTGGTTCTTACATCACAACCAAGAATTATAATTTTGACGACTGATAT
TGTCAGTAGCCCTTATAAAGTTTCGATCCATATAATTAGGTCCGTAATGAACTTCGTTTGATTGATAGGTTAA
GCCACTTTATGGATAGGGAGGTGTCAATAAGTTGCGATTCATATATAAAAAACGAGTATTGCATTGTCGTTTG
GATGTTAACCTTTCACATCTCTCGAGCAAGGTGATCTGTTAACCTTGCATACTGTTTCTTTTGGCACAAGAGC
CTGACATACTATAAGTATACATTAGATTTACTATGTTTTATAGGCTGATGTACTCCAGGAATCTACTATCTCA

ATATTCATGCACCGTGTCATATTTCATTGTTCGTTGGCAGCATGAATTAGTATTTCATTATTAATTCGGACCA
TGTTTTACAACTTAATCTGTGGTTGATTGCTCATTGTAAATGGAAATGGTATATATATATATATATATATATA
TATATATATATATATATATAGTTTAGGTACTTCAGACATACTTCAGGTCCTTCTTAGGCCGGTCTTATTAATA
TTTCTTAAGATTTACGGATAGATAGTGTCTGAAAAATAGATCCAATCAAAATTTAGGGCTGGATGATGATAAG
ATTACCCGTCTCAATCCGTCCCTCACATTCCCATCATAAGTATACCATTCAATCTTGAAAATAGAATAGCTGG
TATAACAAGTAAGCTTTTTGAACTAGCTCATCATGAATCAAGACTTGGACGGGCAAAATATACGTAGTAGGCA
CATTGGCCACTCAAAAAATTATATGTAATTGATATTTTATTTACATTTAAAGAGCTATTTATCCTTTTTAAAT
TATTACCAAGTGTCTTCTTTTTTGGTCGATAAATGTCTTAATTTTTAATCTTTTTTATTTTCCAATTTCTTTT
TGGATTAGACAAGTTATTTTAATCTTTTTTAAAGACACTATTTTTTGATTGGTAAATAAATAAAATTTTATTG
ATCAAATATAAATAAAGTCTAAAATCAATATATAAATTATCAAATACATAATAGATAATTTTTTGATTAAATT
TTATCACAAATCATAAATATAATCTTAATTATAAATCAATAGTTAAAATAATTTTAAAAAAAATACAAATACG
TTTAATAAATATATTTTAAATTAAAGTTTATATGAAGATAGAATAAAAAAGTTAGTATAATATTAATATATTT
GCACTTCAATTTGGTGTGATTCAATTTTCACCATAGAATTTGAAATCCAATCAGGCTGTATAAAAGAAATCTA
ATCTTTTAATATTTCTGTTAATGCTTTATTCTATTTTTAACCTTTTTAGATCAGTTTAGCTGGTTGAAACAAT
TTAAAGCCTGTTGTTGAACACTCCGGCTTCAATGCATAAAAAATGAGATTTGACTTCTTTAAAATAAATAAAT
ATGCTTGAAAAGATAAAATATTAACGGTTAATATTGTAAAAGCGCACACACACACACATATATATATATATAT
ATATATATATATATATATATATATGCATAACAAATTAAATTAATATCAATCTAATCCCTATAAGACGAGTGAT
ATGTTTGTCTTTGTAAAGATGAAAAGCGTCAGATTAGTTGTTTATAGAAATTACATCAATTTAGTGTTTTTAA
ATGAGAAAATATATTGTTATCAATTTAGTCATAGAATAACAATGTTAATGTCCATATTTAATGTTAAAGGATT
AAATTGAGTCATTTTTCTACTGTATTAAAATTATTTAGACCTAATCTTTTGGAACTGAAATCTTGCTTATTTT
ATAGGTACGGAATCGATTATTATTATACTTTCCAATTAAAAAACATATGTTCTAATGAAATTATATTGTTCAG
ATTAATGTAGCAGATATTGTTACATGATCCCTATAGAATCAACCGCAATGAACACAGTATTCTCCTAATTTAT
GCAAAAATGTGAGTAGGTTTAAATTAACAGGTTCAGAAAATCTATACGAGGGAATGAAAACCATTTTATATAA
TGAATATTTTCCTTAACTTTAATTTTTAATAAAATCATGTGTGATGGAGCTTTATCTCTAAAACAAAGTAACT
GGGAGTTAGTATCTATTGTGGATAACATGCATTTCAATAATATTCAACGATTGAAAATGAATTTTATTCAATT
ATAAACCACATGGCTCCTCCTAAAACTACTAAAACAAGTGTTTATTTAAACGAGAATCTCAGCACTCTTTCCT
TCGTCATCCGTTGATAGTTTCAACTGAAGCTAAAAATAGTTGCCATGATGACAAATGACAAGCCCCACCTTCA
AATTTTAATAGTTTACTAAAACCACTTTTGCAGGTCAGATTGCAGAGCCAGCATGCGGCATGCAGCATCAGCA
TGCTTTTCCGCGAGTGTCATTGTTAAAAGGTCAACGACAACTTCCCTATACATTATATTAAATTTGTCTGCAT
AGTACCAAGAAGCAAGATATGGGTTGGTTCTATGATGACTATGTGATAATGTTAAAGTTACTCCTCTAAACAA
AATTGAAAGACTGTAAATCAAATGTTCAATTCAACAGCAAAATCCTTGTTTAATGTAATTATTATGTTATATA
TATATATATATATCATTGGTGCATGGTTGACCACTATCAGTAGGTGTAATTGAATGGATGATAATTCAAGTTC
AAGGCCCCTACTGGGGAACCAGAACCAGAATGAGAATCATGATGGAAACTCTTTTAACTCGCTTAGAACCGCT
TTCTTGTCCAAGCTTCCCGATAAGCTCCGTTCTGGCCTCGACTCTGAGTCTCCCTTTGAGTTTGATCACCATC
TCTCCGCTACCTCCAGTTTAAGCCAAGGTCTCTCTCTCTCTCTCACTAAAAGTTAAAGTTGGAGTAATTAA
TATATTAAATTAGTTTCTGAAATTATGATTATCAATTAATTTAGTTTTTAAAATTAGAAAAGTATCATTTAGT
TACTAAAATTACTAAACAGACATTTTTAGTAACTCATTACTAACCGACTAAAATGGCCTATTTTTTATAATTT
CATGGGTTGAAATGATATTTTCACTATCTAAGGGACTATTTTATTTATTTATTTGTACTTATGATTTCAGGGG
CTATTATTGTTATTATTAAAGTTGTATCTTAGTCTTTTGACCATACACTTAAAGACAACCTTTTAATCGACCG
GAGGTTGTATCACATTCAGTACTTGATTAGATTATGGAATGTACTTTTCTATGATGTTCTAAAATCAATCGCA
TCAAGATAAGATTGTTTATATTTTCAACTCGCAATTTTTCTTGTAGTCTTACTAAATCCCTGCATGAAATGAA
ATTATATAAATCTAATTAGCTTGAAATTTTTTGAAAATTTTGGTTTTCTTCCACGAGAAGCTGCTTCTAGCGG
TAGTCTTGTTAATAGTCACTTCAAGGTGTTGAAATATGATGACTTTTGAGAACAAACTCTCAATCCAACAATC

TTAGCTACATATAACATGTTGAACTGATGTGTACAAACACAAGAACAGATAATGTCCTTGAGACAAGAGTCAT
TGAAGGTGCTTTTCCAAACAAATCAGCTAACAAAGGAAGAAATACAGGAAGTACCAGAAAGATTGTTCAAATT
AGAAGAATATTCTCCTTGAAAGCATTGCAACAAGCCATAAACCTTGAAAAACACAGTTGGTGGAGGCCAGATG
CAAAATGTGACAATTGTCCAATCTTTTCAATATTTTACAGTCTAGATTATTTATTTTTTATTTTTCACTCGAA
AAACAAATTTGAACCTTTACCAAAACCTTATTTTGATCCAATAGTTATTAAGTCACTTACTGCTTTGCGTTTT
GAGTTCTGCAGGAGAAAACCATAAAATATTTAGTTTGGATATTTTGATAGAAGAATTTTGAAAATGTCCAATT
CACAGTGAAGGAATTTCTCTATCACTTTTAGGCAGCTGATTGCTTTTATTCTGTCATAGAATCGTGATCTGTG
CTCAACGAACTTTTCTTTTATTTGAAATAACTTGTTTTCAATATTTATAGGAGAGAAAGAATACTATGGAAAA
CAATTTGCTACTCTAAAATCATTTGAGGAAGTTGACTCCATAGTAACGGCTGACTGCACTGATGTAGAAGACA
TTGAAAAACAAGCTCAACATGAACGAGCAATGAAGATTTCTAATTATGCAAATGCAGCTTTATTGGCAATGAA
GGTGATATTCATAAGATGTATATATTCATTTATCACTAAAACTTCAAGTAGTTCTTCTGAATAGAAGGCATGT
TATTGAAATTTCATAAGAAACAGTTTCTGACACAGTTATTTGAACTTGTCCTAGCATAAGCTCCCTTGATTTC
GGTTAATTGCATGTATCATACTTGTTGACACTGGCTTAGTTAAATAACAATATTTATATGCATATCTAGATTT
ATGTGACAATAAGGAGTGGGTCAATAGCGGTTGCTGCATCAACATTGGATTCTCTGCTTGATTTCATGGCTGG
TGGCATACTTTGGTTCACTCACATGGCAATGAAGGATGTAAATATGTACAAATACCCAATTGGAAAGTTGAGG
GTGCAGCCAGTGGGCATAATTATCTTTGCGGCCGTCATGGCAACACTTGGTGTGTTGAATTTACTTAGCCTAT
TTCTTACTTTCAAACTGTTTATTTCTTGTTACTTAATGTCATTATCTATAATAATTTCCTTCTCTTGGGAGGT
GCATGCAACAGAGTTGTTTGTGTTTCATTTAAATAATCTTTTCATCAATATTTCATTTTCTCTTTTTCCTCTG
ATGAGTATCTCCATGCAGATTACAATGGTGAACTTATGCAAAATTTTCTCATTTTGTAACTTCAAACTTCAAT
TGTGGTATACATATGTACCTCATGACTCATGCAAATATTTGCTAAGACCATTTTATGCCTCAAATTTCTATGA
TATTGCCGTTGTTTGTTGAATCTGACTAGTTGAAAGTTCTAATGTTAGTAAACTTAAAGATCATACCTTTTCA
TGAACAACCGTTGAACAGAACTTTAAATGATTAGGATTTCTAATTTATGGAATTCAAGAATGGCATTCAGCTT
AAGGTTATAGCTAACGATAATTTATAACTTAACACCGGTTTTATATGGTAAGAAACAATCATCTGTTTCCTCA
ATAATTAGAACATAAATCAGAAGATCCAGTGCCCAAACTTTGCCATACAAAATTGATAGTATTCAGCTTTTTA
TCATACTAGAATCTGAGAATTTGAACCTATTTTTGTAACATATTTAAGCATACATATAAATGTTTCCTGAATC
TAGTATACTTGGGTATAGATTATATTGTTTTTACAATTGCTTATAGAGCAACTCAAAAGTATTCATATAAAGG
AAAACCCAGTGCCCAAGGCTCCCACCTTGTGGGGTCTGTTAAGATGTCTTATGATCAATTAGTATGAGGCAAG
GCACAGATTCATTTGGAAACATGAAAAAGAATAGATTACATATAATCCATTTTGATACAGGCTTTCAGGTATT
AATCACGGCTCTAGAACAAGTAATAGAAAATAATCCATCTGAGAAGATGTCTTATGATCAACTAGTATGGCTG
TACTCTATTATGATATTAGCAACAGTGGTGAAGCTTAGTCTCTGGCTTTACTGTAGAAGCTCAGGAAACAAAA
TTGTCCGTGCCTATGCAGATGTAAGTGTCCTTCTCTTTTGAGAATAAAATGCTTTATGAATTGAGTTAAGAGA
GTGTTGCAACATCTTTCAAATTCCAACTTGCAGGATCACCACTTTGATGTTGTAACAAATGTGGTTGGATTAG
TTGCGGCTGTTCTTGGTGATCAATTTTACTGGTTGATTGACCCTGTTGGTGCTATTTTGCTAGCAATTTACAC
TATTACAAATTGGTCTGGCACTGTAATGGAGAATGCAGGTGATTCTGTTACTATATCCATATCAGAATTCCTA
TACTTAAAGAAAAGTCAAGCATTTGCTTTGTAAATTAATTACTTTTTAAAAATAACTTTTGACTTCTTAAAAA
ACTGACCAACTATAAAAAAATATGCTGAAAGAAAAAAAGGAACCATACAATATGATAATTATTAGCAAGAAGT
CTCAATATCCACCTACCTAGTGTTTTGTTGGGATGATAAATCAGTATTTCCAGGGAGCACTAGGCATGCCAAT
CGTAAAAAGTACTAATGTGTTAGTCAAAGAGAAAGTACTTTCTGAATTTCATACTATGAAAAGATTTTTCAAT
ATAGAAAAATAATGAATTATTTTTGTTTTGGGTAAGCTATGATCAGCTGCACCTTCCTTGCATTACCTTATAT
TAGAATGGATTGTGGAGGTTCTTATTATGATAACTTGCTATGCAGTTTCACTAGTGGGACAATCTGCACCTCC
TGAAGTTCTGCAGAAGTTGACATATCTTGTCATAATGCATTCTCAAATTAAGCGCATTGACACTGTCCGCGCC
TACACATTTGGCGTTCTATATTTTGTGGAGGTATGCGTACAAAATCAAATTCTAGACCGTAACCTTATTATAT
TTGTTATTTAATTTGACTTTCCAATGATAGATACATACAAGAATCTAACCCTCCCCTTTTTTGTAATATTTAA

CrMTP8.1	GGCAAAGTATTTTTGTTCCAACTTTGCATAAAACATACATTCACCCTATTCTAAACATGTGTTGTTGTGTTTA<br>CTATGACTTTTCTTTTTTGATATGGATTGTGAATTTTCATTATTATTAATTTCACTTTCCTTATAATTTTGAA<br>CATAACATTTGATGTTCATCTCGTAATATTTTTGTTGCTTGAATTTTTTTAATAAAGTCAAAAATCAAAATAA<br>TTAAATCAAACAAAACTGTCAAAAATTAATTTAATTTTTATAATATATCTCATTCAAACCAAATCAAACCGTA<br>AATATTTATTTACAGCTGATTTGGATAGTCTTTTCCTGTAAAATCATAACAAATTGTACCATAGACACTCCAC<br>GGTAGTATATTAGAAATAATTTTACATCCAATTTCACCTTGAAAAATGTTAACAAATATTTGGTGGAATAAAA<br>TTAACAAGAATTTTTTTTTTTAAGAAAGAAGGGAAAAGATTCTTGATGGATACTGTCCATATTTCTCTTATTT<br>TTCTAGTTTCTTCTTTGTACTAACACCACGATGTATAAAATTAATGTTTAAACATGTAGGGACTAAAATGCCT<br>GTTTGTAATCGCTTATATCTCAACACTAATTTTTCAAAAGCACCAGCAGAAAACTGAAAAAATAAGCATTTAT<br>TTCTTCAAAATAAGCTGAATCAAACATACACACTTGGCACAGATTTTAGTATCAAGAAACTGGTTTGGCTTCA<br>TAAATTTTGAAAGGCATGGCAAAGAAAAGATGTAATAATTTTATGGTGTTATTGGTAATAGGTTGACATTGAA<br>CTGCCAGAGGATTTACCCTTAAAAGAAGCACATGCCATTGGAGAGAGTCTACAGATAAAATTAGAGAAACTTC<br>CAGAAGTTGAGCGGGCATTTGTTCATCTAGACTTTGAATGTGATCACAAACCAGAGCACTCGGTTCTTAACAA<br>TCTGCCAGACACTAAGCCATAA<br>ATGGACAGGAATTCGGGTTCGGATCCCACGTGGCCGCTTCTGGCAAAGCTTGACCCGAACTCCGCCGATAACG<br>GCAGCCGGAGAGGCCGACTCAATCGCCGGAACTCAGTTAACTCGCTCAGAGCAGCCTTCCTGTCCAAGCTTCC<br>CGAGAAGGTCCGTTCTGGCATTGACTCCGAGGCTCCCTTCGACACTGATCTCTCTTCCACCACCGCCTTAAGC<br>AAAGGTCTCTCTATATATCTATCTATCTATATGTATATATATGTATGTATGCTTGGTGTTTAATCTTGTACAG<br>AACAATTGCTCAGAAAATTCATGATTGATTTAGATGAAAAGTTTGTATTATACATTATCACGCACATGAAATC<br>TTACATCGATTATGCTCATAAATTTGTGTTAGGTATATCTTTCTATGATGTGGAATGTTCAATCCGTATTAGT<br>GATAAAGGAATTGTTTAAGAGAAATTATATTTGTAATTTTTATGTATAATAAAGATACATAGGGAAAGATCAA<br>TAAAAGTGTGTGAAAAGAAAAAATAACGTAGTGAAGCGAAAAGTGCTTTATAAATAATAAAAGTCATTATTTA<br>ATATTGTAAAAGCTTTGTAATCTGTGATTTTGAATCACAGATTTGATTTGATTAAATTTTTTTAATTCCTTTC<br>AACTTACAACGTGTTTGAATGTCTGCAGGGGAGAAAGAATATTATGAAAGACAGTTCGCTACTCTAAAATCAT<br>TTGAGGAAGTGGAATACATGGTGTCACCAGACTATGTTGTTGAGGAGGAAGACAATGAGGAACAGGCTCAACA<br>AGAGAGAGCAATGAAGATATCTAATTATGCAAATATAGCTTTGTTGATATTAAAGGTGATATTTCATATTTCT<br>ACAGCTGTTGACTAGACAACTGGTTTTCAATTTGAAGTAGCCACCAGGTTCCTTAATTATGGAGGACAAAACC<br>TAGACATTGTATGATAGATACCCTAGGTATTTTCTCCAATTAAAATCAGCATTTTATCTCGGTAACTGCTGCT<br>TTGATGTCAGTAACTAAAATTAACAGCTATTGAGATGAGATGAGATGCCAATTTTAATTAGAAAGCAACTCTA<br>ACAGTATAAAAGGTATTGTATTCAAATTTTATTTTACAATATAACCTATGATCGTAATAGTTTTCGCTTGATT<br>CTGGTGAGATTTCAGATGAAGCAGTTTCTGACAATTAATTATTTGAACTTGTTTAACTATTGATTAGCTTCTT<br>AAGTGTTTGTAGTTTTACTGTACTTGATGATAATCCTTCTGTGAAATAACAATATGCACATCCGTATCTAGAT<br>CTATGCTACGGTTAGGAGTGGGTCAATAGCTATTGCAGCATCAACTTTGGATTCTCTGCTTGATCTCATGGCT<br>GGGGGCATACTTTGGTTCACTCACGTTGCAATGAAGAACATAAATATCTACCAATACCCAATTGGAAAGCTGA<br>GGGTGCAGCCAGTGGGCATAATTATCTTTGCTGCCGTCATGGCAACACTTGGTGAGTCAAACCGACTTAAATA<br>ATTCCTAGTTTCCACACTGTCTATTCTGATGCCACTTATATGCATATATCAAAAACATTTCCTTCTCCTAAGA<br>GCTACGGAATCTGTTTCATTTAAACTAAATTTATTTCATTTTTTATAGTTAAAAGATGCTTGATTCAGTTTTC<br>AATTTGGATGTGCATCTGTGCCTTATGCTTAAGCTTAAATGTATACCAATTCAAAGGAATCAAATTTATTTAC<br>TATTGCTCTTGTCGATAGAATCTGACCGGTTGAAAATTCTAATGTCTTCGGCAAATTTACGAGGCCATATCAT<br>TCTGTAAACTAATATTTAACAGAATTCTTAAAATTTGTTGGTCATAGCTGATGACAGGTTATTTGCAGAGTTT<br>ATAAAATGATGATAGGGGCACCACTGGGTAAGATAGATGTACGTGTTTTCATTGGACCATAGTATGAGAAAAA<br>ATGCTGCTTCATTGGTATCTTTCCCAAGTTTTTATATATTAAATAGTTTGTCTTTTGCCTCAAAGAATTGACA<br>ACATATGCAGCTTTTGTCGTGCTAGAATCAGACAACTTACTTTTGTTAATGAAACAATGGATAAAATGTGAAC

	TTCAACATACTACATGTTCAAATGTTTATGGTTAGAAACCTACTGCTTCTGAAATTTCATATTTTGGTACTAC
	AGTTTTCACAATGTTGTTCGCCTAGAATCACTGAAAGAAATGCGTACATTGGCATGCAAGAGATTACATGATA
	CGTTTGAGTTAATCCATTTTGCTACAGGCTTTCAAGTATTAATCACGGCTGCTGAACAACTAATAGAAAACAG
	TCCCGCTGAAAAGATGACTGCTGAACAACTAATATGGTTGTACTCTATTATGTTATTTGCAACGGTGGTGAAG
	TTTGTGCTCTGGCTTTACTGTAGAAGCTCAGGAAACCAGATTGTCCGTGCCTACGCAGATGTACGGGTTCTTT
	TCTTCTGATGTTTGCTTTATAAAACAAGTTAAAATACAATTGAACCATCTCAGAAATGCCAACCTGCAGGATC
	ACCGTTTTGATGTTATAACAAACGTAGTTGGATTAGTTGCTGCTGTTCTCGGTGATAAATATTACTGGTGGAT
	TGATCCGGTTGGAGCTATTTTGCTTGCAATTTACACTATCACAAATTGGTCCCACACTGTCATGGAAAATGCA
	GGTATATCCTTACCAGAATTCCAAATAGAAAAAGGTTAACAGAAGAATTTATGCCAGTGAGAGAAAACCCTGG
	TAGGAAAATAGCCATATATAGTGTTTCCAGGAAGCATAAGTCACATCAATTCTTATGCAGATTTTTTTCACCT
	CTTGCTCTCACTTCTTCCACCGGTTCTCTTAAATGTTGGTATCAGTCAAAAGCGAAGTAGTTTCTAAAATTTT
	CCCTACCATGAAAGAACTTTATTTTTTAGTCAACCATGAAAGATTTTATACGCTGTTCTTTGTCAGTTTAATT
	TTACTATACCAGCTGAATTATACACTTTTTTCATTAACTTTTAATTAAATTGAAGCAAAATTATTTACCAAAC
	TGGTATGAAGCATCAATTTCTCTATATTATATGCGATGTGTTTGGAATATTGAGGTAGGAGCAAAAGTGGAAT
	CAGCGAGTGATGCTCCAGCTTTTATCTAATTCGAATGCAGGCGAAAATTAAGGATATTTGGCTCCATAGGAAG
	TTTAGCTCTATTATGTGTCATGACAACAACCCCCGATCCCCAAAATAAACCAGAAAAAAGGACAAATCAGTTT
	GTAAATAAAATTGTATTAATTCTGACTGTGTAACTTTCTATGCAGTTTCATTAGTTGGACAATCTGCACCTCC
	TGAAGTCTTGCAGAAGCTGACATATCTTGTTATAAGGCACCCTCAAGTTAAGCGCATTGACACTGTCCGTGCA
	TACACATTTGGTGTTCTATATTTTGTGGAGGTATGCTGTATTATTCAAAATCAATTTAGAGGAGCACTAACCC
	TCATCGCCAAAAAAGAAACTAATGTTGATTTTTAATTTGGCATTCTCTATTAACGAAGCAATACTTAACAAGA
	CTCTAGCCCTGTTGTTTTCCCTTTTCTAGCACCTAATTTAAAAAAAGTTATTGTCAACTTATTTTCACATTAA
	CATTCACTCCGAGGAATGCATGGCAATTATGGTTACTTATAGATGCGATGTCTCTAAGCGAGCTCTATTATGT
	CATATAAATTATATTGAAGACGACAAATGTTATTTGTTATGCCTCATGGCTCATGATTTGGGTTTGATTTGGA
	ACAGGTTGACATTGAACTGCCAGAAGATTTACCCTTAAAAGAAGCACACGCCATAGGAGAGACCCTACAGATA
	AAGCTTGAGAAGCTCCCAGACGTTGAGCGGGCATTTGTTCATCTAGACTTCGAATGTGATCACAAACCAGAGC
	ACTCAGTTCTTAGCAAACTGCCCAACAATTAG
	ATGGTAATGTCGGCGGGTACCAGCGGACACGGCGGCGCCATCGCCGAGACCGGCAGAAGGGAACAACTCCTGG
	TGGAAAACAGTACCGGTGACACGTCGTCGTTTTCGTGGAGACTCAATCTGAAGGAGTTCCAATTGCCACAGCA
	AACTCATAATAACCATAATGATCGTCACCATCATCATCCTCTTTCCTTCACTTTCCATGGCCTTCTTCGCAAA
	CCCAGTGAGTCATTCTTTCCCTCTTCTTCAATTTACACTCTCTTTGGTTTTATCTTTTTATTTTTTTATTTTT
	TTTGCAATAAACTTGAGTTCTTTGCATTTCATAAAAACTCTCATGCTTAGAAAAATGGGGATCAAACAAACTT
	AAAAAGTTTAAATTATAAATTTTGATAGACTTAGAATCAGTAACATTAGGGTTAAAATATAAATTTTCTTTTT
	ACAAATTTTAATTTGATGTTTATATTTAATTTTTTTATTTACCTCTTCAACGAATAAAATATTTTAGATAATC
	TAGATATTAGGTTTGTCATAACATTATTATAATGTTTCTGGTTAACGTGGTTAACAATTATTGATGTGATTGT
CrMTP9	TTTACTCTTTATATATATATATATATATAAAGAGTAAAATAATTATTGATGTGATTGTTTTACTCTTTATATA
	TATATATATATATTATAATTTTAAATTTTTTTATATGTAATTTTTAAAGTTAATTTTTATATTAAAATATTTT
	AATTTAGCTTTTAAGTGAGCAAAATTATTGAAGATTTGTTAGATTGATTTTTATAATAAATTTTTTATTAAGA
	ATATGTTACTATTTTATTTGACACTTTCCACTTATAAAATATAGATCCTCATTCACTTAAATTTATAATTTGA
	TATAAATAAATATGATTTACAATTAAATATTTGAGATCTCGAAATAATAAAAGTTAGTTTATATATTATCAAA
	GCAAAAGTACGTGGATAAATAACCAAAATAGTAATAAAAAAATAAATTGTCGTTTTCATTCCTGGAAACATTT
	TGAAATTGGTAACATCAATCTTTAAAAAATTAAAAGCTACTTTTTTCGATCCTAAAATATAAGTCGTTCTTTT
	TATATATTTTTTTTTGCAGGAATAAAAATATTACACCAAAAAGAATTAAAATGAATCATTTTTATTTTTTTATG
	GAAAAATTTATTGAATTTTACAAAATTATGCTATTGTTAAATTTAGAGGACAGACTTATTGAATTTTACAAAT

TATTGTATAAAGCCCATATTAATAGTAAAAAAATGCTGACAAGTGTACTTCTCAATACTTTGTGATTGATCCA
TTTTATAAAATATAAAGCAATTTTTAAAAAGTGATAAACTTAAGAAGTAAGTATTTTAACCTTTTAAAAAATA
AATCAACTATAAGAAGTTTATTTAAAAGAATGTGTTATTAACATTTCACATCTCTCACTACCTCCTCAACTTA
AGTAGATATGTTTATTTTGACTCTCTTCTTAATTTTTGGATTTATTGTTTTTTTTTTTTGACAAAATTTTCAA
ATCTTGGTTACAAGTCCCTTTCAGAACTACTTCCATGACAGATGAAAATTTTAATCTTAACTAAAGTGTAAGA
GTAAGTCTATCCAATTTTAATTTGCAATATTTTTTTATTTAGTATAACATAATTTAAATTATTGTTGTTCTGC
ATAAAATTTTAAAATTTAAAGTTTGAAAAGTTATAAAAGAGAAAGCTAATGGTCCCCATTATAGCAAGAACCT
TTTCAAAATATCAGGGTCTATTCAAAAAAAATTTGTGTTATGAGGGACTAAATTTTTTTTAAAAAAAGTACTA
CATATAAATATCAAACTAAAATTAGGAAGCATCAAATTAAAATTAAGAAACATCTATAGGAATCAATCTATAT
TTCAACGAAAAGGGTAAAATATATATTATATCAACATCTGTTAGTCATATCAACTGCACAAAAATGGTATTTT
GACGGATCTAAATATTAAGGATTATTTTAAAAGATTTTTCATGTACAGGAACAAATTAATTTATTTTTACCTA
AAAAATTTAAATTATTCAACACATACAGAAATTAAATTTCATATTTTAATATATTAATTCTAAAATATAAAAT
GTAATTTATTTTAAAATTTTTATAGAGGTTAAAATGAATGATTAAAAAGGATTAAATGAGTTTTTTTTTATTT
CTTAGGATGAGAATGATAAATTTTTATTTTTAATAATTAATTTCAATGATAAAAAGTCTTTTAAAAATTGACG
TCTTAGTATATGACCTCTTAAGTATTTGATTTTTGCAAAACACAGGAAAGAGGAATTTTTTTTTTCCTTTGTT
TGTGTCAGTAGTCTAGACGAAAGAAATGAGAAGGAGGGGCTCATAATTTAATTTAATTTTTATATTTTTGAAT
ATCTTCCAATATTAAAATAAAGAATAATTTACAATAGTTAGATCTTATTTACTAATTAAGTTATCTTGAAATT
TTATGTATTTTTGTTAATGATATCTGTAAGATATAATAATATTTTTTATTTTCTTTGTATTTTTAATAAATTA
TATAATCCTGTATTGAGATAATTTATTTCAAGAGAAATAATTTGTTTTTAAAAATAATTATAAATTAATTAAT
AAAAATAAATAATTATTTTTACAAGAAAATTAAATCAAATAAAAAAATTATTTTCCTTAATTTTTTTTATTTG
TTAAAAAAAATTTCCTTCTTCTAAACCTAAACTTAACAAATAAACCAAACAAGATCCTTTTTAATAAAGGAAA
AGAAACCTCTTTAATATATATATATATATATATATATATATATATATATATATATATATATATCAAAATTTTA
CTTTATTTTTATTTTGTTTTTTTTCTCTTTTAAGTGTAACTATTTAAAATAAAGAAATATTAAAAGTCCTATT
TTCAACCTTTTAAGGCAATAAGTTTACAATTTAAAAAAAATAATAAGAAAGAATATTTTTCAAACAAAACATG
CATGGCATAAATTTTCTGTTATGGATGGGTTGAGTTATGTTGAATTACATGTTGTTTTGTTGTTCGTAGCCAT
GGAGGAATCTTGGCGCAAATGAAACTTTTTCTTTTGTTCTTTTCTTGATTGCGTCGGTGGGGTTAGTTTTTTT
ATCTTAAAAAAAAAAAAGGTAGGTTGAAAAGTGAATTCTTGTGAATTAAAGTTTATACCACCTTGCAACTGAA
ACTGAGTAGGAATTTAATTTATTTTCTTCGAAGAAAAATGAGAAAGCTTGATTGATAATTTGTATGTGTAATT
GATACGGACGAAAGGGTCATGCCTTTTTAAATTTTTAAAAATAAAAAATAATTATATTAATAAAAATATATTA
AAAAAATAAAATTTTTTAAAATAATTATTTTTCTAAATTTGTTTAAAATAAAATTTATATTATTAATATATAT
TTTTTTATTCATTTATCTAAATAAAAGAAAATAAATTTTTTTATTTTTTTTATTTATTTAAATAATTCTTTTAT
TTCTTCTTTCATCCAAATAAATTATAATTATTTATAATTATCAAGATAGATAGATGATTTTAAAATTTTTGAT
GTTCCTGATTAATAATTTAAATTTATTTCATATAGTTATAAATTTTTAATTTTTAAATTAATTTTAAAAAAAT
AATTTATTGAGTACAAATAATTTTAAGTTTATTTTTAATATATTTTTAAAATAATATTTAATTTTTAAAATTA
AAATAAATTTTTTTAATTATTATTTTTAATTTTAAAAAATCATACAACTATATAATTATCATCACTATTATCA
TTATTATAATTACCACTATTATATTTTTATAATTATAATAATAATATTTTAATTTTTATTATTTTAATAAAAT
TTATTTATTTTTAATTTTTTAAATTAATAATTTTTATTTATATATTTTTATAATATTTTAATAAAATAAATAA
AATTATTTAATATATATAAATTATATCTAATTTAATTTATTATAATATTATTTATAATATAATATAATCATATAT
ATTACTACATGTAAAATAAATATTTAGTAATATAAAAAATGTTATAATAATAATATGTAAATTTTTTTATTAT
TATATTAATTATTAAATAACATATAAAATAAAAAAATATATTTTTATATTATAACAATCATTATCATTATTTT
TACAATTATAACTATCATTATCATTATTACCAAATAATAATAATAATAATAATAATAATATTATTATTATTAT
TATTATTATTTAAATATTTAATTTTTATTATTTTAAAAAAATTTAATTTAATTAATTTTAATTGATAATTTTT
ATTTATCTACTTTTATAAATTATTCAATAAAATAAATAATGCTATTTGATATATAAATTATTTTTTATTTTTT

ATATAATTTTATATTACATAATTATTTAAATAAAAATATTATAATATATTATTTTATATAAAATAAATATTGA
ATAATATAAAAAAAATAAATGATCAAATTCTTTTTTCACCTTTTTTATAATCTTGCGAATTAATTTTTTTACT
TCTTAAAATTTTTTCGTTACTCTCTCATTTATAAATTTATCTTATAAATTAATCTTTTAATTAATTTTTGTTT
AACTAAAACTTCTTCAAAATTAATTGGTCTCGCTAAATTTAGTAGACTTTATAAAATTTTTGTTATATTTAAA
TAATAAAATGAATATTTAAATTTTATGTTTTTATGTACGTTATAAAATGATACCATTTTTAACCTCATCCACT
TTTCCATCTCAGCAATGTGTTGTCCAGAGACCATTTTTTTTTTTACCACTCCTCCACTCAAGAGGAAAGCAAG
CAAACTTTCCCTCCATTCTACCTTCATCTTATATTACATCATCGTCACCTCTTCCTTCTCCACCATTAATCAT
CCAAAATCTTCTATTATGTATTGAGCTACCACTCAATCCACCATCTTTTTCTCCTCCAACTATCAATGCCTCC
TCCAAGTCTACCACCTACACCTTCTCTTGCACCAAAATTTCACATGCACTAAAATTTCACAGTAATTTATTCC
GCTGTAATTTTTGGCATATTTTTTCTTTTTTAGTCACTTTTCTGTCCATTACAAGAAATATCACACCTTCGTT
TTTAAATTTGAGAACTTTATAAGTGTTGAATGTTAAAGCAATGATCATACTCCATAAGTTAACCAAAAACTAA
AAAGATATATTATAACGTTATATTAAAAATTTTCCATTTTTTACCAAAATAAAATTTTCTTAGAAGGTTCTAA
TTATTGAATTTAAAAATTATTTTTTAGAAGAAACTAAATACTAAATAATAATAATAAAATAATAAAAATAAAA
ATAATAAAATAATATTAATTAATATCTTGATTTAAAAATTTAGTATAATATTTTAAATTTATTTATTTTTAAA
GTAAAATTTTAATTTGATATATTATGTCACTTTAAAAAAATTTAAATCATGAAAGATAATTCTAAAAAAATAA
ATACTTCTACAACTTTATAAAAAATTTTACATTAAATCCTAAAATTTTTTTATTAAATGAATCTTATAAAAAG
AATCTAAGAGATTAATGAGTAATTCTAAATTCTTTAGCTTTTATTTTTGTAAAATTAAACATTTTGATAGTAT
AGAAAATAAATAAATATTTTATTAATATAAATTTTATGATAAAATAATATTATAAAACTTAATAATTAGCATA
CTAAAATTTAAAGTATATTTTATATTTAAATAAATAATAATAATTTTTATAAAAAATTAAAAAAATATAATTA
ATTCGTTAAAATTGATTCTTTAAATATCTTATATGAATGAGTATAATTTATTATTTTATTTACTTGATAAAAT
GGTAAAAAATTATTTTTACTAGTTATCTATATATGTAGAAAGGAGGAACATGAAACAAACATTAATTTTTTTCT
TCACAATTTTATAGTGAAAATTTTATAGCATGAGAGAAGGTATAAGTGATGAAGTTGGAAGAAACATAGGTAG
GAAGGAATACTTAACTATAAAATATATAATTATATAATATTCTTTACATTATAACCATCATAACTATCATTAC
TACATCACCACCATCTTATCAATACTACATTTATTATCATTATCATAATAATAATAATAATAATAATAATAAT
AATAATAATAATAATAATAATTATTATTATTATTATTATTATTATTATTATTTACTGTAATTTAATTTAT
TTATTTGAAGAGAATTTAATTTTTGTTATTTTTTAATTAATTTTTTGTTTATCTCTTTTTTATAAATTTATTT
ACTTTTGCAATAATTTTTAAAATTTTAAAATTAAAATTAAAATTAAAACTGGATTTAATTTTAAAACTAAAAA
TTAAAAACATTTCGACAAGAAATAAACTAATAAAAAAGAAATTATGAAATTTTAAAGAATTTAGAACTAAATT
GTCATCTTTAAAGTTCTAGTTGATCAACTTCTTAACCATATTTATAAAAATTGATGTCTACAAGCTAATTAAA
ATTTATTAAAAAAGTTGATTATTTTTTTTTCTTTTTTACTTACAAGTATTGAAGAAAAATCTTATCCAAACTT
GGCAATAATATTCATGATGCTTAAATTATGCATATATGAGATAGCATTTTCATCTAGCTTCTAAATAGATCGA
TTTATGGAACGTGTGCTCTTCTACTGCCTAGGATTTCTATTAAAACATGTTTTATTTACTAAATTGGTAACTC
TAATCCAATTTTGTTTCTGAACTGGTTTTCTTCTAAGCAACATTGTGGATATGTTTTTGATTTCAGAGAAGCA
ACGCAAGGTTGCAGAATACTACAAAAAACAGGAGAGGCTCCTTGAAGGATATAATGACATGGATACTATGACT
GAAACGGGTTATTTTCCAGGAAGTCTCACTGAGGTTGATTATTCTATCACCCTCATTCACTTATCATAGTTTA
TAGATTGACTTTAATCTCAGTGAAATTATCATATTTTCCGTTTAAATAACAAAGAGAATAGCATGTTGTAACT
ATCCAGAGTATAAACAACAACAACAAATGAAAACAATGCTGAAGATTAACTATATTCTCAGCAAAACTATCAA
AATTTCCATTTAAACTTGATAACCTCAAACAAATGCTGATGAGTAATTTTTTGAAAAAAAAGAAAAAAAAGGA
GAGATAAAAAATATATTTTGAATTAACTAGTAAATTAGTTCTCCATGTAGGCATTAGAAGTTTATCTTTAACT
TTAAAAATATAATGTAAATTAGTTTTTTTATTTTTAAAAATATTGTAAATTAGCTCCTTCATTTATTGAAAAT
AATGCAAATTAATTTCTCTGAAGATAATTTGTAAGATTTTTAGAATAAAGAACTAATTTATATTATTTTAAAA
ATTAAACATAAATTTATAATATGTGCATAAAATGAAAAATTAATTTAATATATTTTTATTGACCCAAATGTGA
AGTGAAAAAACAATCGATAAAAGAGAATATATAAATGATGATCAAAGATGAAATATATATACATATATGTATT

GAAAATACTCTTTCAACTGATTAAGATGTGAATCAAATTAATTTCAAACTGAAAAAAAAAATATATTCATTGA
TCAAATCATTCACTCCAATATAGTCTGATGAATTTTTTTTTAAAGAACTCGTTAATCAGGCAAAAATAAAGAT
AGATCCCATTCTACATGACAATACAGACAACAATAAAATTTAGGATGCTATACAACAATTTTATGGCATAACG
GATGATCTATCGTCCAAGTTTTGTGATTTTATTAGACAAATAATCTGGTTTTAAAAATAGGTGAATCACAGTC
CAAATTTTTATATTTTTTAAACTATTGCTGATTTTTTCACTCTTAGTTCCTTATTAGTCCTGATTTTATTTGC
TGTAATTAAAAGATAAAAGTTGTTTGCTTAAATTTTTTACGAGTATTTTCTAAACAACAGAACATTAGATGTT
TCTCAGGATGAAACGAAGCAACTAGCAAAAAGTGAGAGGTTGGCAATTAATGTGTCAAATGCGGCTAACTTGG
TGCTATTTGCGGCAAAGGTTTATGCTTCAATTGAAAGCAAATCATTGGCGGTCATTGCTTCAACCTTAGATTC
TCTTTTGGATCTGTTGTCAGGGTTCATATTGTGGTTCACAGCCAATGCCATGAGAAACCCAAACCATTATCAC
TATCCCATTGGAAAGAAACGGATGCAACCAGTGGTATGTTAGACTCATTCTTTGTTTGAAAACTCATTAATTT
TTAAGAAATAATTCCTTAGTTAAAAAAATTAAATATATTTTTAATTCTTCTAAAATTTTAAAATTTTAATTTT
AATCTCTCATTTTTTAAAATATTTGATTTTGATTTCTTATTTTTTAAAATAAACCATTTTAATTCTTAAAGTA
ACTAATATTTAAGGCATCAATGTTAAAGGATTAAAAGTAATTATTTTAAAATTGAGGGATCAAATTTAATGAT
TTAAAAAATAGAGTCTAAAATTAAAATATCAAATTTTAGAAAAATCAAAAATATATTTAACCTAGAAAAATAT
TCCTAAAAATTGATGAAACTAAACAACTGCTCTCTGTATTTCTTTTTTATCTATCGAGAGAGAGAGTAAAATA
TTTGTTTCTATTTTTATTTTAAAAAAATATTAATTAATATTAATTATTATAAATTATTATATGTACCATTTTC
ATTACTTAGAGAAAAATTAAAATTGTAATTAATGAAAAAAGCTAAAAATGATGTTTATAAAGGACAAATCAGA
AAAGTAAATAGAATTTTTTTAAAAAATAATTATATTTTTTAATATTTTTTACTTTTTATATAGATAAAAAAAT
AAAAGAAAGAAATATTATATTAAAATAAGTTAAATTAAACATGCACCTAATGTTAATGTTGATATGTTAGTTT
AAGTTGAATATTGAGTGAAAATTTTGGTAAATGTAGTCTAGCAATAGTTTTCTATTGCATGAGTGAAATGACA
ATAATTGGATATGTTTAATCAAATTAGTCATTGCATTTTTGAGACTAAGAGACATACATATTAAATATTAACC
AGGACTTTCTAGGCAGGGTATCATTGTTTTTGCATCAGTGATGGCAACCTTGGGATTGCAGATTTTGATAGAG
TCTGGGCGGCAACTGATTTCAAAGGTAATTGAGTTCAGAACTTGTAAGCTTTTGCTCATTGTTATGAATTGTG
AAGTGTAGCACAGTGAATTGTTCTATTTTTATAGATTTGTTTTGAACCTTTCTTGTACAACTATTTTTAATAT
CTTGTTTCATTTGGTATATTTTACAGTCTAAGCCTGAAATGGATCTTGATGAGTTGAAATGGATGATAGGGAT
TATGGTATCTGTCACTGTAGTAAAGTTCATTCTCATGGTTTATTGTCGAAGGTTTAAGAATGAAATTATCAGA
GCCTATGCACAAGATCATTTTTTTGATGTCATTACTAATTCAGTTGGATTAGCTGCTGCTGTCCTTGCTGTCA
AGTGCTCTTGGTGGATTGATCCTATAGGAGCTATTATTGTGAGTTCTTTACTACCATTACATGGCTTTCTTAC
TGGATATTCCTTTTTTGAAGCAATGGATCTTATGTTAAAATTATGTGATCATGTGAGATGAAAATCTTGAGTT
TATCTCATTTTTCATAGACATTAATTGAGGGTCATTTGAAAGAGGTTATAGGAAACGTATAAAAATATTTATG
AACCTAACAAGTTATTTTAGTTTATTTTAATAAGTTTTTTTTTTGGTTATAACTTATGTTCTTTAATTTATTT
TAGGAAAAATTATATTTATATTTATGAAATTCATTAAAAAATTATCTAATATCTTTTTATTTTTTTTATTCTAC
ACATTTTTTAATTTACAAAAATATAATATTATATATCTCTTTTAGCTATACAAATCTATACAAATTTTTCTAC
TTTTTTATAATATTTTATGTATAAATTTTTTTAAAAAATATAAAATATTTTTTCGAAAAATCTCAAGGAATAT
CAATATAAATTAGTCTTTATTCTAATATAAATTAATTTTATGATCATATAATAAAAGGTAATAAAAAATATTT
AATTAATTTTTTTTATCTAAACTCATTTTATGTAAAACTTCATTAAAATTATTTGTAAATATTTATAACGAAA
GGAAATAAAAAAATTTCATACCACAAGTTAAAATTTCACAAGCTTATAAAAAGAGAAAACGACACATAAAAAA
GTTCACAAAATTTAAACAGATTCCATTTTCAATTTTCTTATGAAGTGTTTATCATTTGAATATTCTATTAGTA
TATTATTTTCTTTGACTTGGCGTAATGCTACAAGGGTCATAAACACAAATCATTTATAACCAAATTCTCAATG
ATTATTCTTATGTTATTTGTGTGTTAATCTGTTTGTTACAGTAAACCAACTATAGCAAAATTGATTGTGATAT
ATACCCTGAATATCTAATGTAGATAGCAGTGTATACAATAAATACATGGGCGAAGACAGTGATTGAGAATGTA
TGGTCACTTATTGGAAGGACAGCGCCACCAGATTTTCTAGGAAAGTTGACATATTTGATATGGAATCACCATG
AAGAGGTTAGGCACATAGACACCGTAAGAGCATACACATTTGGTGCTCATTACTTTGTTGAAGTCGATATAGT

CrMTP9.1	GTTGCCAGAGGACATGCCTCTCAATCAAGCACACAACATTGGTGAGACACTGCAAGAGAAGCTGGAGCAGCTT<br>CCTCAAGTTGAAAGAGCCTTTGTGCACATTGATTTTGAATTTACTCACAGGCCTGAGCATAAGATGATGGTAT<br>AA<br>ATGGCAGAGAGTGGCGGCGGACCCCGCCGTAGGGAACCGCTTCTGGTGTCGCCGGAGGAAGAGGCTGCAAACC<br>CTTCGTGGAGACTCAATGTGAAAGAGTTTCGTTTGCCAACCCAAACTAATGATCAGCAACATCACCATTCCTT<br>CACTTTCCGTGGCCTTCTTCGCAAACCCAGTGAGCCATTCCTCTCTCTCTCTAAAGTTTCAGGTTTTCTGTGA<br>TGGCTCTGTTCCTTTTTTCTTTTTATAATGATGTGTTGAATTGTGCTAATCCAATAAGCCATTCTTCACTCTT<br>TTCTGTGTTTTCTTCCCTTGAACGTCTAAAGTTTCTGTTTTTGTGTAATGGGTATGGTTTGTACTTAGCGATG<br>ATGTGTTGGATTTTGCAAACCCAGGAAACCATCCGTCACTTTTTTCTGTTTTTTCTTCTTTCGAACGTCTAAA<br>TTTTCTGTTTTGCGTAATGGGTATGGTTTGTATTTGGTGGTGATGTGTTGAATTGTGTAGAACATCAATAAGC<br>CATTTTTTAATGTTTTTCTGATGGGTCTGTTTAGCAGTGGTGCGTTAGTTTGTGCAAACTCAGCGTGCCTTTA<br>TTGACCTTTTTTTCAGTTTTCACCACTTCTTATTCTCTTTGATGTCTAAAGTCTTTATTGTGTTGAATTCTCC<br>AAAATCAGACTTCTCTTGAATGTTTGAACTTTAAAGTCTCCGACTTTGTGGGATTGGCCTATTTTTGTTCTCT<br>GTGGTGATGCTTTGAACAGTGCGAACCGAAGAAGTCGGAGCAGTGGTGTTATTATTAACCTTTGTTTGTTATT<br>TATTGTAGAACACGGTGGGAATCTTTATTAAAGGAAAGACCTTTAATTGTAAATGTGTATCATTTTGGTGTTC<br>AGTGATAGTATCTAATTGAGTTTGGTATAATTCGATTCATTTGTCCAAAAGTAAATGAGTAGAGAAATTAAAA<br>AATACGGTAAAATCTAGATGTACTACCATAAATATTTTTTGTACTGTTCTCTAATCAAATCTTAAAATCAGAG<br>TTTCACCGATAGCCTGTGTGGGTCTCCGGGCTTGCCGAGCATGATGGATGTTAGGATCCAATTCTAACGTATA<br>GGACTTGAGTCCCACATTGGAAGTATGGATTCTAATGTGGGACTTTGGGCTCTCCATCTACACAATTAGCTTT<br>TATAGTATGGTTCTTCCAAGATTCTTATCATAAATTTTTACTTTTATTGTGTCAGTTATGAATTAAACTCCAA<br>TTTATTATTATTTTTTTTGTCAAATGAAACTCCAACTTTTAATGGAAGAACAAAACGAAAAGGATTTTATGAT<br>ACTCCATCTAAGTTTTATGCTTGGAAATAATGTCATGCCTTTTCGTATTTCTCCACAGTTACTAGTTAGTACT<br>TAGTACTATTTCTGGAGACACTCCATTGCTCATTTCATTATTTTTCTCTTGTTAGAATCTCTTGTACTTGATT<br>CTATATATAATCAAGAAAAATGCTAACCACACACTTTTCAATGAAATGCATTATTTGTTATTGGTTTTTTATT<br>TAAAAAGTCATAGTACTTTTTTAAAAATTGATTTTTTTTTAAAAAAATAGACTAATGCCTAAGCAGGTATGAAA<br>CTTCCTTCCCTTAATGCTTTCTCATATGATATACTAAAATGGATCCGTTTTGCTTCTGAGATCATTCTTTTAT<br>ACCCTTTATCATCTTTCCTTAAAGGATAACATAGTACTTAACAAAAGTTGACTATGTTCAATTACTTGTTCCC<br>ATAACATTTAAATTTTAAAATATTTAATTTTAAAAGGTAAGTGATTTGAAAATTTTGACATTGCTGGTTATTA<br>GTTAGCTTGAGTAATTCATAATTGTGGACATGTGATGGTGCTTGGGTCTAGCTTGTGAACTAGTTTTCATTTC<br>CATTGTTTGCTACCTAACGCTGCCATTCAACGCCATTTTTATGTTTTTGCCTACTTACTCTCTATAATCTCAT<br>TGTTAACTTATGTTTTAGGGAAGCAACGCAAGGTGGCGGAATATTACAAGAAACAAGAGAGACTCCTTGAAGG<br>ATTTAGTGAGATGGAGACTATGACTGAAACGGGTGATTTCCCAGGAAGTCTCACCGAGGTTGATTGTTCTATA<br>ACCATGATTTACTTAATGTGAAAATTTATAATTTTCCAATTAAATTTGATCATATCAAACGCACACAAATGAA<br>TGATTCTTTTGAATCCATTGAGAGAATTCTACTAAATAAACATGCTCAATGTTCCTCAGGATGAAATGAAGCA<br>ACTAGCTAAGAGTGAAAGGATGGCTGTTCATGTGTCAAATGTATGTAACTTGGTGCTGTTCGCAGCAAAGGTT<br>TATGCCTCAATTGCGAGCAGATCGTTAGCAGTCATTGCCTCAACTATGGATTCTCTCTTGGATCTCTTGTCAG<br>GGTTCATATTGTGGTTTACTGCCCATGCCATGAAAACTCCAAACCATTATAACTATCCAATTGGAAAGAAACG<br>CATGCAGCCAGTGGTAAGACTTAATTTAAACTGCACAAGGTGAATGAAACCTATAAAGTTGTAGTAAATGAAA<br>TCTACCGATAGAGTTTCACAATATTTTGAGAGTGAAAATGATAGTAAATGGTTTTCTTATTTTCTATATGATA<br>TTGATTCTTAATTTAATCATTGCTTGGTTAGTTAATAAAGATACATGCTGATATGACTTCTTATGCAGGGTAT<br>CATTGTTTTTGCATCTGTGATGGCAACATTGGGGTTGCAGATTTTGATTGAGTCTGCCCGGGAACTTATATCC<br>AAGGTAAATTGAGATCAACTTTTGTTCTTGTTTGTTCACTAGATTGAGGTGTATAATATAAGTGTCAATTATC<br>CCTTTTTTGGATTTCCTGTAATTTTTACCATGACACTTTTTATAAAAAATTTCAACTATCATTCTTTTTCTAC

	AGTCTAAGCCTGATATGGATCCAGTAAAGCTACACTGGATGATTGGGATTATGGTATTTGTGACAGTAGTGAA
	GTTTGTTCTTATGATTTACTGTCGAAGATTTAAAAACGAAATCGTTAGAGCATATGCACAAGATCACTTCTTT
	GATGTCATTACTAATTCAGTTGGATTAGCTGCTGCTGTGCTTGCTGTCAAGTTCTATTGGTGGATTGATCCAA
	CAGGAGCTATTATTGTGAGTTCATATCCACATCTTATTTTCTCCTCACTCTGCTTTTTCTATAATTTTCTGAT
	TACGTTCATATTTTCAACAAGTGTTTTTTTAATGAGTTTACTTAGTATTTTAATCTTTGATTTAATTAGTTTT
	TGCAAATTTATTTGTAGCTTTAAAATAATACAAATTAGTTCTTATTTTTTAAAATTTTAAAAATTAATCCTTT
	CATTTACTTAAAATAATGTAAATTAATCTTTCTGAAAATAATATCAAGGAAGAATTTGATAAACGGTGATAAA
	TGTTGAGAAATAATGGAAAAGTGTTTTAAGTTAGGAAAGTATTTATAAAAGTGTTTTGTCGTGATATATTTAA
	TTTTAGTTCCTTCCAAACATTTTTTTTGGTTTTACTGCTCGATTTTTCCAGAGTCAACACTTTTGGTTATTAG
	TAACTGTTAAATGGTGACGATATTAATGAAGAAGTTGCAACATGTCAATTATGTGTGCGGCCACGTCAATGAG
	ATGGACCAAAATACCTATTAGAAGTATCAAAACCAACAAAACAATCTAGGGGGACTAATAAAAAAGTTGTGAA
	AAGGACTATAAACATATTTAAGCCCATTTTCATTAATCTTTTCCATTATTCTAGAATGGCCATAAACTCATTA
	TTTATGCCACTTAAAAATCTTGAAAGGAACTTTATGTGATAAATGATTGTTTGCTTGTGATAATGACAATATC
	TAATGCAGATAGCATTATACACAATCAATACATGGGCTAAGACAGTGATTGAGAATGTTTGGTCACTGATTGG
	AAGGACAGCACCACCTGATTTTCTGGCCAAGTTAACTTACCTTATATGGAACCATCATGAGCAGATTAAGCAC
	ATAGATACTGTTAGAGCATACACCTTTGGTGCACATTACTTTGTGGAAGTTGACATTGTGTTACCAGAAGACA
	TGCTTCTCCACCAAGCACACAACATTGGTGAGACACTCCAAGAGAAGCTGGAGCAACTTCCAGAAGTTGAAAG
	GGCTTTTGTGCACATTGATTTTGAGTTCACTCACAGACCAGAGCATAAGACCATGGTATGA
	ATGGGCACTGAACTTAGAACTGATTCCTCTGATTACAGGACAGAGCTTCTTTCCCCAACAATAGCTGCAGAGA
	ATGTGTCCATGGCAAGGCAACCTTCATGGAGGATCAACATGGATGAACATCGTTTGCCAGAAAGGCAAATGGA
	GTCTCATTTTGGCTTTGGATTCTTCCTAAGGACACTAAGTATATATCAATTGAGCACTTTTTTTTCCCCATTT
	ATTGTTTCCAACTTTCTCTCTATAATTTTTTTTTTGCTAATATAAAGTTTGCATCTTTAATTGTTTCTCAGAG
	AGACAAAGGAAACTTGCACAGTATTACAAGAGACAAGAAGGGCTTCTTAAAGGGTATCAAGAAGTGGACTCGT
	ATCATGATCTGGGCATGTTACCAGGAAATTTAACAGAGGTTTTGAAAAGATTAATTCACTCATTTATTTCTTA
	ATATTATTATTATTATTATTGTTTTAAGTGGCATCTTTGATGCAAACATGAATGGGAAAATTTTGATTTTCAG
	GATGAGATGAAGGAGCTTGAAAGGAATGAGAAGGTGGCAATTTATGCATCTAACATAGGGAACATGGTGCTGT
	TTGTAGCAAAAGTGTATGCTTCCATTCAGAGTAGATCACTGGCTGTAATTGCATCAACTTTGGACTCACTCTT
	GGATCTCTTGTCAGGATTTATACTTTGGTTCACTGCTTATGCTATGAGCAAACCAAATCATTATCAATATCCA
	ATTGGCAAGAACCGGATGCAACCTGTGGTAATGACTTATTTGAATCTAAGATTCCTCAGTAAATTTAGGAATT
	GAGACATAATTGAATTTCCAATTATTTTATATTTATGTTGAAGATTTCATTCATTTTTTTTTTTTCCTTTTATT
CrMTP10	TTGTGTGTGCCAATTGTTCTCACAGGGGATAGTTGTATTCGCATCAGTCATGGCAACTCTCGGATTACAAATA
	TTGTTTGAATCAGCCAGGGAAATCATCACAAAGGTATAGATGTAAAGCATATCTTACACCCTCAATAGAAAAA
	AAGAAGAAAACTTAAATACATTATTAATCTTTAAAAATATCATGATTTTTAATCTTAGTATATGAAATTTTTT
	TATTTAATTACTATAAAGGACTATAATAACCACATTTTTTTTATAATAACTAAAACTAAAATCTGTGATATTT
	TCATAGGCCAACAACTTATTTAAATAAACAAAATTAGCATAGCATTTTGTGATAGATTGTCTATATATAAATT
	ACATGTACTAATTAATGTATGCTTCATTTATTGACAGGCTCAACCTGAGAGGGATCCAGTGAAAGAAAAGTGG
	ATGATAGGGATAATGGTGACTGCAACTTTGGTAAAAGTAGTGCTAATGACTTATTGTCGCAGGTTCAAGAATG
	AAATAGTGAGGGCCTATGCTCAGGACCATTTCTTTGATGTCATAACAAATTCAATTGGTTTAGCCACTGCAGT
	TCTAGCCATCAAATTCTACTGGTGGCTTGATCCTGTTGGGGCCATCCTGGTATGTCCTTTTTTAACAAATTTC
	ATTAATTAATATACTCTCAAAGCTTTAACTATATTGTATTTTATACCTTTCCCATTTTTAGATGACATTGACC
	ATCATAAGAAATTCAGAGAACTATATTAAAACAAGAATGTTATGTGGTGTTAGTTGTATATATTGAAAGAGAT
	TTGTTTAGAATAAAAAGGGTGGATAAGTATGTTTTTAGATTTAAATATGATAAAATTTGGTTTGAGTTTTTCA
	TTTTTTATTAATTTTGATTTTTCATGTTTTAAATTTTTTAAAAATAACCTCTATCATCACTTTGTGTTAACAT

	GGTATGTATATCTTGATATGCTACATCAATAACCATTTTAAATGATAAAGGGTATGATGTTTGGGTGCATAAC
	ATATTTTTAGTATCAAATGATATAGATTGGTTTTAAAAATTTAAAATTTGTGAGAATTAAAACTAATAAAACA
	ATATTTAGAAGACTAAAACCAAATTTTATTATATCTATAAGAATTAAAAATATATTTTATCATATATCATTTA
	ATTTTAGAAGGATTAGTATGTGTAATTTGTCTTGATATTTCTATAGTACATAACTTTTTTTTTTTAAGATCTA
	TTTTGCAAATAATAATGCAAAGTAGTATATGTAATAACTGGAATAATTATATATGCAGATAGCTTTATATACA
	ATCAGTAACTGGGCAAAAACAGTGATGGAGAATGTATGGTCATTGATTGGTAAAACAGCCCCACCAGAGTACC
	TTGCAAAGCTAACATACCTTTGTTGGAACCATCACAAGGAGATCAAGCACATTGATACAGTGAGAGCCTACAC
	ATTTGGGTCCAATTACTTTGTGGAGGTAGACATTGTGGTTTCAGAAGAAATGTCACTTAATGAAGCACATGAC
	ATTGGAGAGACACTTCAAGATAAGCTTGAAAAACTCCCTGAAATTGAGAGAGCTTTTGTTCACATTGATTTGA
	ATACCACTCATAAGCTGGAGCACAAGTCAAATTAA
	ATGTTGGAGCCGGTGGAGCTTCGTAGCGAGGAGCAGCGCTCGTTGCTTTTAGATTCCAACAATGGTGACCGGT
	CTTGGCGGTTGAACTTCGACGGATTTCAGATATCTTCGGAGCACACAGAGAAGCAAGTGAAACCCCCTCGTGG
	CCTCCATGACTGCTATGGGGTTCTAGGTAACACTACAATAACGTATTCATTCCATTTATTTAATTATTTTTAG
	TGTTATTATTTATTTTTGGAATCTGCTTTTTTATTTTGGAGTTGACAAAGAATTTCTTGATTTTTCTTGGGAA
	TGAGAATCTGCAATTTGCTTTACATCAGTGGTTGAGGTTGATCGTGAGTTATGTGCTGCTAGATTATTTTTTT
	TTTCTGTTCTTGTTGATTTGTTGTTCCCTTTCTTTCTTTATATGTATATGTATTCCCTTTGCAATAATGTTTT
	TATTTTCTTAAAGATGCTATTGCTGGACTTCGTTCAATGTGAGTGCGCCATGTTTTGTTCCTTTTTCTGCCAG
	GTTTAGTAACACGTGTTGGATTTTGGAAGCAGGGAAGAGATAGAATGTTTGTCTTTTTGAAGATTTGGGATTT
	TTTGGGTTGATGATATGGATGGCTTTGTTATTATATGTTGAAGTTGAAGTGGGCTTTTCATGTATTGGACATT
	TCTTGCTTTCTGGGGCAGTTGGAAGATACGAAGTTTTGGGTTATGCTTTGTTTGTTTTCTGGTTTATGGAGCT
	TTTTCGTAATTTATCATTTTGTCCTAGAACTGTGAAGAGCTGATTCAAAAAAATATCATTTTATTAACTGGTA
	TTCAAAAACCATGGAAGTGTTTACGGCTTAACTGTGATATTAAAATATCATTTTATTCATTTGGGAATCTCTG
	TCCTGTTTTCTTTTTCAAGGGTGGTTTTCTATGTTTCTGAAAGGTATTCTTTGAGGGATGGCATCTTTGCTAC
	TAAATTGGAAGGGGATTTTGTGGATCATGTCTAAATTTTCTGTTTCCTTGTGTTACGTTTTTTATTTTTCAGG
	TCAAGAAGATAATATTGCCGAGTACTATCAGCAGCAGGTTGAAGTGCTTGAGGGCTTTAATGAAATGGATGTC
	TTAGCTGAACGCGGTTTTGTTCCTGGAATGTCAAAGGTTTCTATTTCATTTTCTCCTTTATGCTCTCTAGATT
CrMTP11	TAAGATCGATATATGGTCAAATGCATTGGCTGAATATCCACTGTTGTGGGCAGGAGGAGCGAGACAAATTGGC
	GAGAAATGAGACATTTGCCATCAGATTGTCAAACGTAGCAAACATGGTTCTGTTTGCTGCCAAAGTTTATGCA
	TCAGTCCGAAGTGGTTCCCTAGCCATCATTGCATCCACCTTAGACTCGCTTCTTGACCTCCTTTCTGGATTCA
	TCCTCTGGTTCACTGCATTTTCCATGCAAACACCAAACCCATATCAATACCCTATTGGAAAGAAACGGATGCA
	ACCATTGGTCAGTCCTCTTAATTAATGTTGTATTTGAAATTTTCTGAATCATCATCTTCTGTTCTTGCCAGGA
	TTATGGCCTCAGTGACTCTTAGAAGTTGTGTTGTTAATCAAATTAAATATAAAGAAAGAAAACTACCAGCCTA
	CAATTTGGCCGAATACACATATGTTCCATGGATAAAGTATAAAATGAAAAATCGTTTTTAAGTAGTCAATGGC
	CTTGTGGATAATTATGACTACATCTTGGTGCTGAAATTTATAGTCAATTTTTGGTGCAGGGAATCCTTGTTTT
	TGCCTCTGTCATGGCAACACTGGGACTGCAAATAATTTTGGAGTCTATCCGCACATTAATATCCTCTGTAAGT
	TAATTTTTGTTGTACTTCTGGAAGCAAAATTTTCATTTAGTTACTGGATCTCTAAATATCTAGGCAGCTCTAA
	TGTATCTTGTGATGACATTTCCAGGATAATGCATTCAGCTTGACCAGGGAGCAAGAGCAATGGGTTGTGGGTA
	TCATGCTTTCAGTGACTTTGGTGAAATTCTTGCTAATGATTTATTGCCGCTCTTTTACAAATGAGATTATTAA
	GGCCTATGCCCAGGATCACTTTTTTGATGTGATAACTAATGTCATTGGCCTTATTGCTGCACTTTTGGCAAAT
	TATTTTGATGATTGGATGGATCCTGTTGGTGCTATCATTGTAAGTGAATACTTTCCTTATGTTAAAGGTTTTG
	ATTATATTTATGTTAACAATTGATAAATCTGATTCATGCCTGGGGATGAGGTAAAGCACTGTAGTATACAAAT
	AGTATTTCCAGTAATCTCATCCATCAACAACCATAGCCATATGAGTGTCTCAGCCTCTGCTATGCTCCTATAT
	TTAAAAAAAATAAAATCAAGATGTCAGATTCCTTAAGATGGAATTTCTCATTGCAGAAGAGAACCTATCTAAT

CrMTP12	TAGCTTATCATAATTTCTACAATCTCTCGAATTCTATGCATATTATGATGATGCACGGCTTTTATACTCAATT<br>CTGGACTACTACGATTTTAGGGAATCACGGGGGTTCTTTTGAGCATAATGTTTTGCTGGAAACAATTTCTTAT<br>TACTATTTGTATACCTAACTTGTCACATAGCTGGTCACTATAATTTGTACTTGCTTTACTGACATGTAATTCT<br>TCTACTGCAGCTGGCTTTGTACACTATTCGCACATGGTCCATGACAGTGTTGGAAAATGTGAATTCACTGGTT<br>GGAAGATCAGCTGCTCCGGAGTATCTTCAGAAACTTACATACTTATGCTGGAACCACCACAAGGCCATAAGGC<br>ACATTGATACTGTTCGGGCATACACATTTGGGTCTCACTACTTTGTTGAGGTTGATATTGTCCTGCCAGCTGA<br>TATGCCTTTGCAAGAGGCTCATGATATTGGCGAATCATTGCAAGAGAAGCTTGAGCTTTTGCCCGAGATTGAG<br>CGTGCTTTTGTTCATCTTGATTATGAGTTCAGTCATAAACCTGAGCATGCTCAAGCACACTCCTAG<br>ATGGCCGATCAGCCTCACCACCACCACCATCGACCGCACCGCCTCTCCGTGCCACCGCGCACCGCCACTTTGG<br>CGACCACCCCGAGACCCTATCCCACAGTTCGATACTCAACATCAGCATCAACACCCACTGCAACCCCGTCCAA<br>ACACCGTCGTCTTTCTTCTTCTTTATCCTTCTCTAATTCCAAATCCAAATCCAAATCTTCACTCTCATCGCTT<br>TTCCTCCTTCTCTTCTCTCTTCGCTCTCTCTATTCTCTCCTTCCCTTCCTTCGCTCTTCCCCTTCTTTTTCTC<br>TATTCCCTTTCACTTTTCTCGTTTCCCTTCTCTCCTTCTTCCTTACCCTCTCTTTTTCACTATTCTCTTCATC<br>TTCTTCCTCCAAAGATCCTTTTCAACAGCACAAACCTAAGCAGTCTCTTCTAACGGTCACACACTCTCAGCAG<br>AGGATCCTGGTTGCTAAATCAATTGTTCTGGCGCTTGTTTTTCTCCTTCGGTTCCAAGCTCTCCGCTACTGTG<br>GCACCGCCTCAATGATCCTTGCCGAGTTGATGGGAAGCATAGCTGCTCAACCTAAAAGTCAGAACCGGACTAA<br>GGTTCGTGGGTTCGTGTCCTTGTTTCTAGGATTGTTTATGTTGTCTTTTGGTTGGGATCGAATTGAATGTTTT<br>CCCGTTTGGGGATATGCGAAATGTGTGAGGGTTTGGCCCTTGTTGCTTCCTTTTATGTCTGGATTTCTGGCTT<br>GTTATGAACGTGTTTCCATCATGAATTGGGGAACGATTAAGCAGCTTGGTCATAAACGGGTTCGCCTAATTAC<br>CCTGTTTTTCACTACTATTGTGCTATTTGTTCCTGCTGTGATTAGTTTTTTCGTGCTTGAATCTGAAGAAGAT<br>AGTGTTTCATTTGGAAATTTGGTGTGGCCTCTGGCCAATACTGTTGTTTTCGGTGTGCTTTTGAGTGAGAATT<br>ACAGCGATGATTATGTGATGAGTTTCAAAGACTCTAAGAGGGAGTTTTTGGTCACTTTTGTCTGCACTATGGT<br>ATTGGAACTTTTCTATTACCCTGAACTTTCCCTTTGGGGTTTGTTGCTTTGTGGTATATTGCTCTATTTTGCC<br>GTTAGAGACTTGGATCCTTTCCATTCCAATGAGTTTGGCTCGGGAGAGGAATTATTTAGTGACATGATTATGA<br>GGCCTATTAGACATGTTTTGGGTGAGAGGAAGTCCCGGAAAATTGCACTGTTTCTTTTGATCAACACTGGCTA<br>TATGGTTGTGGAATTCGCTGTTGGATTTATGAGCAATAGTCTTGGGCTGATATCTGATGCATGCCACATGTTG<br>TTTGATTGTGCTGCTTTGGCCATTGGGCTGTATGCTTCATATATATCCCGTTTGCCTGCAAATAATCACTATA<br>ACTATGGAAGAGGAAGATTTGAGGTTCTATCAGGATATGTTAATGCTGTTTTTCTGGTTCTTGTGGGTGCACT<br>GATAGTGGTGGAGTCCTTTGAGAGGATATTGGATCCTCAAGAAATATCTACTAGTAGTTTGTTAGTCGTCTCT<br>ATAGGAGGGCTTGTTGTCAATGTCATTGGCTTGATATTCTTTCACGATGAGCATCATCATGCCCATGGATTGT<br>CTGGATCATGCTCACATTCTCACGCACACACAGAGTTCCATAGTCACCACTCCCACCATCATCATGATCTGCA<br>ATGCCACAGTAAACATCATCATGATGAAAGACATGGAAGCAATCAAGAATTTATTTCTGTTTCCAGTGATTGC<br>CAAGATAATTCATGCTCTGGTGATCCTGGTCATCACAACCACTCTAGTCATGGGAGGAAAGCTGAGTTTCATG<br>CAGACAATCACTGCAGTAAGAGTTTTACGCATGATCATGCCCAGCTGCATGATTGTCATGAACACTGCAGTCA<br>TGAGAGGATGGTTGAGTGTCATGTAGACAATCACAGCATTAAGAGCATTGAACATCATGGCCATCAGGACCAT<br>CATCATGGCCATCAGGACCATCATCACCATCATGGCCATCAAGACCATCATCACCATCATGGCCATCAGGACC<br>ATCATCACCATCATGGCCATCATGACCACCATGATCATGCCCAGCATCATGAATATCATGATCTTCACAAAGC<br>TCCACTTGTTCACCAAAAGCACAACCATCGCCATATCGATCACAATATGGAAGGATATTCTTGCATGTTCTT<br>GCTGACACGATGGGGAGTGTTGGTGTTGTTATATCTACACTATTAATTAAGTACAAGGGATGGCTTGTTGCTG<br>ATCCTGCCTGCTCAATTTTTATTTCAATTTTAATTGTATCCTCCGTGATACCTTTACTCAGAAATTCAGCCGA<br>AATTTTGCTCCAAAGAGTTCCAAGGGTGCATGAGCATGAGCTGAAAGATGTCTTAACTGATGTTTTGAAGATA<br>AGAGGCGTTTATGGTATTCAAAAGTTTCATTTATGGAACTTCACAAACACAGATGTGGTTAGTACACTGCATC<br>TTCATGTGTCAACAGATACTGACAAGACATCCGCGAAGTCCCAGGTTTTGCATCTGTTACATAGTGCTGGAAT

	CAAGGATTTAACCTTGCAAGTGGAATCTGTTGGATAA
**Promoter region (ATG upstream 2000 bp, 5′ to 3′)**	
CrMTP1-Pro	ATAGGATATTTTATTAAAGTGGTGTTTGATTTTAAAAAATGTTTATTGTTTTTATTGTTTTTATTTTTATTTT<br>TATTATGTTGTATAGAAAAAATAAAAAAAATATTATTTTTAATACAAAATTTTAAAAATATAAAATAAAATAA<br>AAATAAGATGATATTTTTTATAAATAAAATAAAAAATTAAAAATAAAAGCAGAAAATATTTTCTGAAAGTAAA<br>TAAGATTTAACTTTTTCTTGTGATGATTTAATTTATTTCTTTTACTTCTTAATATATATAGTCTCATAATTCA<br>TTAATTTGTTTGTCTAATATTTTTTATTATTTATTTTTCTATTAATTGTGTATGTTGACATCTTTTGAAGCTA<br>GAAATATCTGAAGTATAATTTTTACAAACTAAATTGTATAATTATTCAATGAAGTAATTAAAATTATATGTAC<br>AAATATATATATATACACTTATTTTTTTTATATTTTTGTCCTGTTTATAGAAGTTATTAGTTTTGATCTTTAT<br>CATTAAAATTATCTATACTTTTAGAAGGTGTTTGGTATATGTACTTTTTTTTCATTCTTAAAAATTATGTTTT<br>CGTAAAATTGTAAATATATTTGATAAAGTTGAAAAATTCACAAAAATGGTAAATGAGTATAGTTAAAATTTTT<br>CTATTTTTAGAAGGATATAGGATTGTCATTCTTTGTATGAGAATGTTTTAGTAAAGACTATAGTTAAAAGTCA<br>TTGTTACGAATTTGAGTGTTTCAGGGATATTTTTTAAAAAAAACATTCATACTATACATAGCAAGCTAATGTC<br>CTCGAATTAAAATTCTTGAAAAATTGAAACGAATCCTTCTAATGAATATCTTTCCAGTTCTAAAAAATGTTAG<br>TGCCCACAATTTCATTCTGAAATTTGTTCATTTTAAATTTTTGAAAATTATTTAATTCTCAAAATATATTTTT<br>TATACATTTTGATTTATGATTTTACTAACTTTATTTTTATTTTATCTATATATTCTTCACAAATTTACAAGAA<br>AAGCATCATAATATAATTAAATTAATATCAAAATGGTTATATTGATATCTATTAAAATTAAAAATTTTAAAAT<br>TTTAGTTTTAATTCATTATTTTAAATAAATTTATTTTGGTTTCTCATTTTCAAACCAAACTACAGTTACTTCC<br>TCATCATTCCTTATCATTAATGTGTTGAGCATTAAAATATTCCATCTATCAACCATTAGTTACTCAATAAAAT<br>AAAAATAATTTATTTTTAAAAATAAGATTAAAATCAATCATTTAAAAAAAATATTAAAATTTTAAAAATACTA<br>AAAAATATATTTAACTCAAAGTAAAAGTATAGAATACAACTTTACTAACTGAATTGTATATTTACTCAATCAA<br>ATAATCAATATCATTATTTAATAGAAATCTTTAATAGCAAGAATTAAAATAAATAATTTTTGTAAACTAAAAA<br>GTAAAATCATAAAAAATTGATAAAAAATAAAAAATTATTAAATTTAAAAATAAATAAAAATTAATTAAATTAA<br>AGTCATGATTCATGATAGACACGTGAGGCCTCCCAACAAAAAAATAGAAGAAGCGCCATCTACATGCATGATT<br>GAGAGAACTTTCTTTGGTAAGAAAGAATATTCGTTTTTCTATTTTTTAAAAGTATTAAAAGCAATTATTCTTA<br>AAAAAAATTACACAATTATACAATGTAGATATATATAAAGTTAATTGTGCAAAGGTATCCGTTTATTAAGGGC<br>AGTTTTATTTGTAGAATTTTATTTTATGTTTGGCTGAGTCAAACTATTCCGAATCAGGAGATTATAATAAACG<br>AGAAAACATGGAATAATTTTTTTGTTTCTATAAAAGAATTTTGATCTGAATGTATTTAATAAAAAATAAGCTT<br>AGATTTTAATAAAATTTATATTTAACATTTAATAATACGAATAACAACAAAAATGTACACAGAAAAAAAAAAG<br>GAACAAAAATATGAATAAATGTTTTCTGAA
CrMTP4-Pro	CATTCCTTTAAAATTATAAATTATTTTAAATTTGTTATACATTATTTATTATTTGTAGTTATTTAAAATTTAC<br>TTTTTTTAATTTTTACAAAGTAAAAAATGATATCAATTATATTTTTTTATTTAATATAATTTTTTTATTTTGT<br>TTGAAATGTTTTTATTATTAGAACCATTTTTAAGTATTTATAATTTTATTTTATTTTTTTTTTATTTTTAAATT<br>AAAATTTACAGTTAATTGTTTAAAATAATAATAATTTTTTATATTAAAACTAACCACTTGCTCCTATAAATTT<br>TATATGTAAAACTTTTAAAATTCAAAGATTTTTTAATTCTATGAATCTTTTGAATTCGAATCCATAAAATTAA<br>AATTAAATAAATTAAAATTAACCTATTTAACAGTTCTACTTCCATAAAAAAAATACTAACTACCCAGATAAAA<br>AAGAGATACGCTTATAGAATTCTCTTTCAAGAAAATTTGAACGGTTGCAAGAATCTTGTCGTGCATATTCTTC<br>ATATGGCCTATAATGGTAAGATTATCAAAGCACTAGTTTTCCTTTCGAAAGACCACTTTTATTATCCAACTGT<br>ACGAGAAATTAAATTAAGTAATAACATGTGATATAATAATTAATGGTGATTTCAGATCAAATTCACTGTGCAA<br>ATCTTCATCAATCCCAGCCCAATAAACCAAAAAGTTAGAGCTATCTAGTTTCGAGTAATCTGGTAAAACGAAA<br>ATGTAACTTGATCCTAATCAATATATGCATTCTCAAATCTAATAAATCCTAACAATTGTATCATTTTATTTTT<br>TACTATTGTGGTGATGCGTATGTCTATCGGGAATATATTGAATTTTGTGTTTATTAAAACATATAAATATAAT<br>TTATTATTAAAATTTTGATTGCAAGATTTTAATATATATATATATATATTATCATTAGTAATCCTATGAACAT

CrMTP5-Pro	ATTTTTTTAACATATATATTTTAAATTGATTTTTAAAGGCTAATTAATTTATGAAATAATATTTTAATTTTTT<br>AAAAATAATTAATTATAAAAATATATTGAAGAGTATATTGTTCTGTCATTATTTTGAGTATATAGTACGATCT<br>TTTATTATACATTATGGTGTTATTTTCTATACACATTTTAAAAAAAAATTCAATAATTAAATTTAGAATTTAA<br>CTTTGATCTAATTCAATTCAAATTAAATAAACATACAAACATCTTTGTTAATTCATGTGATATTAGATGAAAA<br>CTTATAACAAACTCAATTTAACCATAATCTAATTTAATTGATTTATATTTTTTCCAAACCTGATCTATCTTAA<br>TTCAATTTAACCTTTATCAAATTCGATTGATTTAATTTTTTTTAAACCCGGTCTATCTTTACTTTCTGTTCAT<br>TTTTTTAATCATTAGTATGTTATCTGTATGTTAGTCTTTTTAAAATTAAATAAAAAATTAAATTTATTTGACT<br>AAGATTATAATTATTTTAAAATTTAGTTTATCTAAAAAAAGATATATTCAAATTTGTTATAGTACACCAGTA<br>TTAAATATGTAAATTAAATTATTAATTACAAGAAATAAATATTATAAATAAAAAATTAAATGCTTAATATAAA<br>ATAAATGACATGATTTAATAAGAATATAAATAGTTTCTAAAAATATTTCAATTTAACATAGAAAGATAAATTA<br>CTATTTGTATTCATAGATATATGACATTCAACGATACGTTTTTTATTTATTGTAAGTAAGGAATCAATAGTTC<br>TAAGTTTTTGTGTATTTTATATGAATTAAATTAAAGTTAAATTATTAGGTTTTATAAAATAAATAAAAAATTA<br>ATTAAAATATACTCGTAATAAACTGATCATGAATTAATATATATTAAAATTATTAACTTTTATAATAATTATT<br>TAATTGATTGAAAATATAATTTTTTAATCATGCATCAAAATTAAATTTAATAAATAAATAAAAATAATAATGT<br>GTGGAATACTCGTGTGGTAGTGAGGTAGTA<br>TGATAGGAAAACAGGTTGATGAAGCAATGCCGGAATAAAATAGAAACAAAATTTTCATTATTTTTAACTCTAA<br>AATAAATAAATTAGATAAGAATTATTTTTTTGAAATTTGAAATGAAAAATGAACCCATCTCATTCTGTTGATT<br>CTCTAGATTTTTAATGAGAATTTCTATTCCTTCAATATTTTATATCGTTTTATATATTTGAGTGAGAAATAAA<br>AAAAGGATAAAGATTAATGATGTAATAAAGAAATAGAAAAAATAAATTATTAAAAAATGTTATAAACTTTTGT<br>TGTAAGAATAACATAATTCTTTTTGAATGAACCATATTATTTAAGCTATTGAAACTATTTAAAATAATTTTCT<br>AATCATGTACCTATATATTGGATAAGAGTATTGCATCGTCTTTCTTTCTTTTACGATAACACTAAATGCATAA<br>ATTAAATCTAATTATACGTCTTTTAATTAACTCATTTATTTAAGAAGGATAATTATATACAATTAAAATTTTT<br>GTGAGATTCTCTCTCTCTCCTTTGTGTATGTTTTCACTTTAAAGTAAATAAATTAAATAATTTTTTTTTAAAA<br>AAATTGAGATGCTTATAAAAAAATGAACCCACCTAATTATGTTGGTTTTCTAGTTTTTTGAGGAGGAATTTTCT<br>TTTCCTACTTAATATTTTATAACATTCTATATATTTAAGTATGAGATAAAAAAAGAAATAAAAAAATGAATGA<br>TACAATAAAAATATAAAAAAAATGATAAAAATGTTATAAAATTTATTATAGAAATATATAATTATTTTTAAATG<br>AGGCATATTATTTAATTTATTCAACTAAATATTCAAAAATAATTTTGTTAATGTATGTACCTATGTATTGGAT<br>AAACATATTAAATGTATAAATTTTCTTTTAAGGTAATATTAAATGCATAATTTAAACCTAATTATTCGTATTT<br>TAATTAAATCATTTGTTTTAAAAAGATAGCTATGTATAAAAAATTAAGATTTTTGTGAAATACTCCATCTCTT<br>TCCTTAATATATTTTGTCATTTTTCAAATAATTTTATTTATTTTCAATTTATAAGTATGAGAAAAAATAATAA<br>AGAGATAATATTTTATAAAATCTTAACTTACCTCTATATAAAATTAAAAATTTTATTTTAAAAAAAATTTAGA<br>AATTAATTTTTCATATTAAATTATACAAAATTTTATATATTTTTTAATTTTAGTTTTTTAAATACTAATTTAA<br>TTTAAATTTTGCATATCAATATTATTTGAATGTGTATTAATTTTTTTTTATCATGAGGTATAGTCCCCCTCCCC<br>TCCCCACACCCCCATCTATACATCCAAGTCTAAAAATCTTAATTTATTTTTTTTTGAAGTGGAAGAGTCTTAAT<br>TTATTTTCATTCTAGCATATTCTATATTGAACCCCTATGTCTTTAAAAGCAGTTTGGAAACTTTTGAAATTAC<br>AAGAAAACTAGTTGATGAAGTCATATAGGAATAAAATAAACAAAACTTTAATTTTTTTTTAACCTTATACTAA<br>ATAAATTAGATACAAATCGGTTGTTTGAATTTTGAGATTGTTATACAAAATGAACTGACCTCATTCCGTTGGT<br>TTTCTAGTTTTCTAATGAGGATTTCTTTCCCTACTTAATTTTATAACATTCTGTACATTTAAGTGGGAGATAA<br>AAAAGAAAATAAAATGAATGATATAATAGAAAATTTATATGGTTAGTTATTTTAAAAATTAAATTAGTTGAAA<br>TTCTTCATTGACTTGTACTAACAATTTAAATTTGTTTAAAAATTATTATTTAATCATAAATTGTGATATTATT<br>AAAATAACTAATATTTATAATAATTACATTACAAATTGCACTTATTACGATTTTTAATTAATTAATAATATAA<br>TTTTGTTTACATCGATAAAATTAATTAAAAGAGATATTTAGATGAGGTTAATTTGTGTATTTATCAGGCTAGT<br>CCATAGCTAGTTAGAACTTAGAAAAAAATCG

CrMTP6-Pro	TTTTAATTATATATATATATATATCTTTATTTACTTAACTTACAAGATTTAATATTGTTGCATAAAAAAATTA<br>TTAAACAAGTATTTGAAAATTTTATTTAATAAATATGTCAAAAGAACAATTCATGCCTTTAGTGTTCAATGGT<br>TCGAAATGTATAAAAAAGAAACATTCTTAAACACTTGGTATATAGGATATGTAAATTGTATTCTACAACACTT<br>CATTTATCCATACAAACAAACCAAAATCATTAGCAAATAAACATAATTTTCTTATGAAAAATAAAATCATATT<br>AAAAAAAATTTAAAAATTTTTCCATCATCACTCAAAAAAAAATGCTAAACATATTGTATTTAGAGCATAATTA<br>CATAGAACAACTTTAAAAAAAATATGTTATGTAAATAATATTATATATTAAATAGAAGATAAAAAATATTAAA<br>AAATTTAATCCATCTTAAAATGATTATTTTTTGTCATTTTTATATAAAGAAAATTTGTTAAAAAAATATAAGA<br>ATTTTTTTTTAATCATTTACATCAAAATTAAATTTTCAACTTTTTGTTCTTCAATTTTTTCTTATATTGAATA<br>TTCTGTTTAGTTGTTATATGTAAAAATAATAATTTTTTTTTATCATTATTCATCTTTTAATAACAATTTTTT<br>TTTTACATTTACTTAACTTTAAGTTTGAAGATACTTGTTCAATACTATTTAAATTTTAAGTTTAGAGAACTTT<br>GTACTTAAATATTTAGTTAGTTGTATTTGTTATATAAAAATATTTTTAGTACAATTTGTTAGATTTAATTGGG<br>ATTAAATATATCTCTCAATTGTTGAAAATGAATTATTAATATGATGTTTTAATTTTCTGTTTCTTACATCTCT<br>CATTTTTTAATTAACTAGATTAATTGTAATATTTATTTTTATTTTTATTATTTTAGTAAACATTTTATATGTT<br>TCTTGTATTATTTTAAACATGGATTGGTTTAATTTAAAATAGTCATATGAGATAAATGATTTGATTCAATTTC<br>ATATTTTTTATTACTTCAAATTAAACGAAACTAAAGATAGATTATATGTCTCATTAAATTTAGTAAGTATTTT<br>TTATTAAAAATCTAAATAATTAATTATTTTGACTCAATATTCTCTCTAATCAAAATCATATAATATTGCACAT<br>ATAAAAAATAAAAATAGAATAAAATATATATTTTGTCAAAATGATTATTTTAAATAGCCATTATTTAACTAAC<br>ATAATTAGGTATTTTACATGTTAGTTAGAAATAGAGGTTTTACTTGTTTGAAAATCCCAAATCAATACTCAAA<br>CTAAATTTGACTAAATTTTTATTGATTTGTTTTGATTTCAAAATAAACATATAAATCAACTGAATTGGTTCGG<br>CTTCATAAAAATCAATAACATCCCTAACAAAGTTATAAAAACTTAAACATATCGCCATTTTTTGTAACAACAC<br>GTGCCAGCATTTGATTAAATGGCTTAATGGTAGAGATATAAGAATTTTAATCTGAATTATTTCTTTAATATAA<br>AAATCTATCCATTTTAAGTAATAAAAAAATGAAATTATGAGTAGACAACTGATTCAAGTTTTTAAATGCAAGT<br>CAACTAAATCATTTAAAATTAACATTTTCAATATTTACTTAAAAAAAATGTAAGTTGGATCAAATTACTTATT<br>GAAAATTGAGTCAAGTTAAATCATTTTGACGTTGTTGGTTTCAAGGGTAAAATTTATATTCATCCCCCTCTCC<br>CCCTCCAATTAAATGGCACATAGATATTTTTTAATAAAATAAAAAAAGATTAGTATGTGTTGTATAATTTTAA<br>ATAAATATGTACTGATAATTTGATTAGGAAAATTAATTATAATTAAAAACTTTGACCCGATTAGAATATTGTT<br>TTTAAAAAATCTTAATTCATGTTCGGTAAGAGCAAAGATTCACCACGTGGCGCGCGAGTGTTATGACTTAATG<br>CGCTTTGTGACACGTGTCATACCTCACACA
CrMTP7-Pro	ATAATAGGGAGATGTTTGGCTAATGTTTTATTATTTTTATACAAAATTCTAAAAATAATAAAAAGTATAAAAA<br>AAGATTATATTTTTTAATAAAAAAATTATTTTTTCAAATCAAATGACACCTTAACAATACAACTACTATTGGA<br>TGATCATGCAGAAATAGAAAATGAACAATAGGAACAACAATCTTCAACAAGATTTAGAAAAGGACAGGATAAT<br>TTCTTTTCTTTTGAAATCATGTTTATCCAATGAAAAAATTGTTAGAATAGCTAAAATTTGTGAAGACTTTTAA<br>GGATAATTTTCAGATAAATTAGGCCATTCGAGATCGTACATTGATCCTTCGCAAGTTAAGAAGAAATTACACT<br>CTAAGTCATGAATAATGTTTAAATTTCAGTATTCTTTACTTTAAAAAATTACATACTCCAATCGTTAGAAATT<br>AATAAATTTCTCATTCTAAAGAAGCAAAATTTATTTCCTTCTTATCATGTCTCACAAATAAATATTTTACCTA<br>TCTAAAAAGAAGAAAGAAATGCACATGTAAGTAGGAATACACTTACCATAAGTATTAGACACTTTACTTAAGA<br>TCTTCCTTATTTTAGATTTTGACACAACTGATTTAAAAGAAATTTGTTTTATAAAATTGATTTTTATTTGGAC<br>ATGATGTTTTAAAATGAATTGAAAAGAATTGATTTTATTTGATAGATGAAGTAGAAGTATTTTCGAAAATAAA<br>GATACAATATAATCTATGGATCTCATATTCAAAATTTATTTATTAAATAGAAAAATTAAAAAATTCATTTCTT<br>CTAGAATTAATTGTCTACAGCAATTGGTTCTACTAATCACTTTTTTCAAAATTGATATGGGTAACTACATGTT<br>TATTAAAGATATCCTCCATTAATAGCTAAAGATTAATACATACACACAGTCAAAAATTGATCCTATCAGCATA<br>CTTAGAAATATAACTATTATCTATATACTGTGTTGAGCTTTTTGATACTGTACAATTGAATTTAAAAGTAAAA<br>TAAATGGGTTAAAAAACATACACTACCAATAAAAATTTAAATAATCTTTCAAAATCATTAAGGTCTATTGGGG

	TATCATAAGTTTTTTAATGCTAATTTAAAAATAAAATATTTTTAAGTAATACGAAGTTAAATTATCTTTTTAA<br>CTCGATTCTGAAACTTTAAAGAAAATTTTAAAGGAAAATTTTTAATAAAAATAAATTTTCAATTCTTCTATAT<br>TTTAAAAATACACACATATTATAATTATTATCATTATTTTTTTTCTATTATTCCATCCACTATAATTATTATA<br>ATTATTAATTTATTTAGTTATTTTTTTACATATACCTTTATATTAATCTAGTTATTATCAACAATAATAAATA<br>AATAAAATTAAAATTAAAAAATAAATAAAAATTAATTATATTTTAAAATTAATATTTTTTATTTTAAATAGAT<br>AAAAAATAGTAAAAAATTATTATCCCATTTATTAACATTTCATTTTCTATTCATTTTAATATAATCATTATTA<br>TCTATATACAATTTCATAATTATCATTATTATAAATTTATAATTGTTATGATGATCACCATCTTTAACATTAA<br>CATTAAAATTATTATTACTATTATATACAAATTATACTTATCACAAATTTAAAAAATATGATAAATAATAATT<br>TAAAAGAAGAAATGATAAATAATTTATGTAATTAAATTTATAATATGTTTGATAATAAACTATTTTTAAAAAA<br>TAATTACTATTTCTTTTTAAAAAATCTTATAATATTAATAAATAAATGATTATTTCATTAAAACAAAATACTG<br>ACTTAATAATTGACATTAAAGTTGAAAAGCCGAAAATCTAAAAAATAAATTCTAATTTGTATTTTAATATTTT<br>TAAAAAATTACAACTGGTTCATTCAGGTTCAGACATTTGGAAGCAATTTCCTTGATGATGTTGATTGGTGTGC<br>AAACCTTTCTCCAAAATATTTAAAAAAGAA
CrMTP8-Pro	GATTATATTTAGGAAGATCCTGTTTTATGTTAATCATAATTTCTAAAACATTAAAAATATGTTTGATATATTT<br>AAATAGTTTTTAAAAGTTTTTTTTGTGAGTTTAACTAATATTTTTTTATTAAAATCTTAAATTATCACTCATT<br>TTGTGAGAATATTTTAATAAAAAATATAAATAAAATGCCCTTAAAAAATTAATTTATGAGAATATTTTTTAAG<br>ATTCATATTTAAATTAAAAAATAAATTAAGGTTCAAAATTTAATTGAAAATTTTATTATTTTATCATATAACC<br>TCTTAATTTTAAAGGAAATATATATATATATATATATATATATATATATATATATATATATATATACATATAT<br>ATACTTTTTTGGTCAGACTTCAATTTTTTCTCTCCTCGAATGTAAAGAAAATTCAAAATTTAAGAAAATAAGT<br>CACTTTTTATTTCATTCCCTTAAAGAAATTATCATAAAATTCTACAATTTTAATTACATAATAATCAATAATT<br>ATATGATAGCATAAATTTTATTTTACAGTATTAATAAATTTTAATCAAAATCAACATTCAATACATGGTATGA<br>ATATTTATAAATAAACTTAAATATTTATAATCTCTGTAAATATACTTAATTTTTAGTGTCTATAAAAATTCTT<br>TTATTTCCATCACTAACTATTTCAAAATTTAGCTTTTGATGTTTGTTATAAACTTGTAGTTATAGATTTAATT<br>AATATATGTTTGACAAAATTTTATTGCATGTAGTAATTTAGTGTATACATTAAAAAATTGCAGAAGTAATTAA<br>TTTGAGTGAAATATTAAAATAATTTTTTTACATGATTCAAGATGAAAACCAAATTTATATTTTGTATATTTTT<br>TAAATATATACAATAAATTAGTAATAATTTTTTTAAAAATATTTATTAATCAAAAGAAAATATCTAACAGTAA<br>AAATTAAAAAATCTAAGATAAAAATAAAATAAAAAATTTACAATAACTAAAAAAATTTTCTTGTATCTATGAA<br>AACTAAAATATATTTAATCATATCAAATATATGTGAAAGATAAAAAAATTTAAAATTAATTTTTTTAAGAAAT<br>ATTATTTATAGATAATAACAAATAAATTAAAATTCAAATATAGGAGATTAAAACTTTAAACTTGGAAAATATT<br>AAAAATCATTTTAAGTACTAAAATACAATTCTAATGATGATATATTAAAATCTTATTGATTAAAATTAAACTA<br>ACTAAAGTTATTGAATTTTGTTTGATTTTACAACTATTTAAAAAGAGGAAAAATTATTAAATTGATATATGAG<br>TTAATAAATACGCATCATTACAAAATAAAAAATACACGTAATTATATAATAACAAGAATATTTTATAAAACTT<br>GTTTTATTTTTAAGAATGAAATATAAATTTATAATTAAATATATATTATGTAAAATTGATGATTATGTGAATT<br>ACTAGTATTCAAATTTTAACATCAAATATCAATCCAAGTCCACGTGATAAAAAAATATTAATTCAAAATGATT<br>TATTTCAAAAATAATGTTTTAAAAATTTAGTTTCTTTTGACGAACAATGTATAGTTTTTTATTTTTAATAAAA<br>TAATGATACATTTTCATTTACAAAATTAATCATTTTAATACTTCTAAATTTAGAACTTTATATTTTACTTAAA<br>GTAAGAACTTTTATATAATTAAAATAAAAATATTTAAATATATTTTTAATTTATTCAAAATTTTAAATTTTAA<br>TTTTATTCATATTTTTCAAAATAAATATTTTTAATTTCTTATTTTTTTATAGATAATTTTTAATCTTTGTTAA<br>ATAGAATATATATCAAGTATTAAATATTAATTTTTAAAATTAAAAAAAAATATATTTTAAAAATAAAAAAGTA<br>AACTAATATTTTTATAATAAAAGATTAAGATTAAAATTTTAAAAAATTAAACCACATTTAATCCAATAAAAAT<br>TGTGGTATCAATTTATGTGTTTTTTTAAAT
CrMTP8.1-Pro	ACGATAGAGTCGTGATTACTCTTTGGCAGTTTAGCTAACTTGTAGTCTTGTACAGTTTTTTTTGTACATTGTA<br>TTTTATTTATTTTGTCCCGTAGTCATTTATGTATATTAAAAAGGGGGCTTTTGCAATTATGGAAAATTATATA

	CCACAAAATGAAGTGGTCCATCTCAAATAATTTATTAATAATGTCCGCATGTTTGTCCAAAGTCAGACTGTGC
	TAAAATGTTAAATTGGTGAAACGTAATAAATCAGTAGGCATCCATAGCCATGGCTTTTTCTTTTTGGCTTTTG
	CCATTTTTTCCTTACTGTAATTTACAATTTACAGATTCCATTCACTCCTCAATCATTTTTATTCACTTTCAAA
	CTAGTATTGCTTAAAGATTTTCTATATTTTCACTCTTGGAAAACATATATATATAAATACTGTCATAAATATA
	ATTACTTTTTTTCTATTTATAAAAATTTTGAATTTTTCTTCCTTTTTAATGAAAATATTTGGTTGAAAATATA
	TTGTAAACATATTTCCATTCTCCAAAGTATTTTTTGAAATATTTATATCAAATTTACTTATTTATGATGATGT
	TAGTTTGTAAAAATATTTTTTATTTTTTATTTATAAAAAAAATATTTATCTTATTTTTTATTTTTAAAAAAAA
	GGTTAATATGAATTTGTAAAAACAATAGAAACTGTGTTTAGTAATTTCATTATAAATTTTGGAAAACATAAAA
	TAAAAATAAAACAACAAATTTATAATAAATAAATAAAAATATTTTTACAGATTAAGCAGTTCCTAATATTTTG
	AATTTTTTATTTGTAAAAAAAATTAAAAAGGACTTCTTACTAAATAACACCTATGATTAAGAAGTTATTAATT
	TTTTTAAAATAATAAAAATTTAGTTTATTACATTTTTATTCTATTTAGTATATATAGTTAAATAAAATAACTA
	ATATTTTCTTATTTTTTAAATGGTAAATAATTTAGAATGAGCTAAAACATCTAACATAACAAAAAGAGAATTT
	ACCGTGTTTATATTGTTATTTTTTGTTGCAAAAAGTTTACTTTTATTAAACTGTTTTACTTAGAAAAAGAAT
	ATATTTAAAAAAACATTAGAACGACAAGATGTTATAAATCTAAATCAAAACGTTTCCTACTTAATTGGGGATA
	TATATCTTTCAGTTTTCTATTTTTCTCTAACCGCCGAATGGTAAAGACACAAAATGAAATCTTTAGAAAATGA
	CCCTATTAAGAAGGAAATTAACTTTAAATTTTAACTTCTTCAAAGCATAGGATTCCTCAATAAAACACAAAAT
	GCAAATCATAAACAATAATAAAGAATATAGAAGACAGATTTTGTTAAAAGCATTATTTTTATTGAGATATTGT
	AATATAATCAAAACATTATATTTTTTTTCATGGACAAGAACTCCCCCAAAAGAAACCGTGTACTTGAAAATGA
	AAAACCAAATGCATAGAACTTGACTACTTACCTAAACATTTTTTTGTTTTTGTTGGGTAGAATATCTTTTTAA
	GATTCATCTAGTTCGTCCACTTTCGACCTTTAGCAATGATAGTTTGCAAGAATATATAGATTTTATCTTTTCT
	ATTAATTGTCTATATATACAAAATATTTATCTATGAAAATTCACTTAAATTTTCAATTTTATAAAACTTTCAC
	GTTAATATTTAATTTTCTAACCTATAATTTATCTTAATATTTTTACTGCATTTTTTCACTAGCATTAAATATT
	TAAATAAAAATTGTATAATATTTTGTCAATTAAAAAAAAATGATGAACAACACTTACAGCGGTATTATATACT
	CCTTTATCTGTGGTCGTGAAAACTCACCCTTGTGAATTCAACGGAGGCGTGACTGAGCATCAAACTCTTCAAA
	GAATATTCAAATTGAATTTTAACATAACGAGTGATGTTATATATATATATATATATATATATATATATATATA
	TATATATATATATATATATATATATATATA
	GTAAATAATGAAATATTTAAATGCTCGAAATATGTTATTTTACCTCTACGTTTTAGAATATGATTATGTTAAA
	ATTTAGCACGATTATACTAAAACTAAATTGTATGAATTTTTAATAATTTATAAATGTAACATGGTCATTCTAA
	TAGTTAGCATGATTGCATTAAGAATAAATTTTATAAATTTTTAAATTTGTAATTTTAGCATAGTCATAATGAT
	AGTTAGCATGATCATGCCTACAATAATTTGTATAAATTTTTAGGCCAGCATTTGAGTATGATCTTGTAATTCA
	TTAGCATGGTTTTGAAGTTCATTGATCAAGGTTGTATAATTAATTAAGTATGATTATGTAATTCATTGAGTAT
	GGTCGTTCAATCCATTGAACATAATCATGTCAAACTATAAAGGTGAAGATAAGGAGGACAAAAATTAATACGA
	GTAAGCAAAACATGAAAAATATGATTGTACATGTAAATTCACAACGTCATGCAATGTCACATAAATATAGTCA
	GGTAAGATATTGAGTACGATGTACAATTTATTTGAAGATAAAAAATTGTACATTATTATAATGAAGAGATGAA
CrMTP9-Pro	AAGTTCAAAATATTAACAATGTGTTTTGTATTAGATGAAATTAATTAATGAATAAAAGTTAGCAACCTTATTT
	GATGTATAGAGATTATATATTTAATTTTATAGAACTATTGAGATAAGTACACATATAATAAGTAAAAATGAAT
	AATGAGAAATTTTAATAAGAATGTAAGTTATTTAGTTAAGAATGATGAATGAATATATTGAATAGATGGTTGA
	TCATGTATCATATACATACAAGATTACTGTGTAAATCATCGATATGAGGATAATTTACGAGATTCTTTATGTG
	AATGATACATACAAGATTATTATGTAAATCATCAATATGAGGATAATTTACGAGATTTTTTATATGAAGTAAT
	TAAAAAAATATAAGGGTTGACTCTTGATGGAAATAAATTATTTATAAAAATAAAAATTTTTAATCTTAAAAAGT
	ACCTGAGTTAAGTTTGAGTCTCGCATGATATAAAGTTATTTTTTGTCGTTGAATACAATTATCTTGATTAAAT
	CCTGAAAATTATCGTTACTAATTAATAAATTTCATAATTTTTGTAAAATATTTTAAAATAATGATAAATATAA
	AATGATGTTAATATTGTTAAAAATAATTATCACCTTAATTATAATATTTTAAATTATTTATATTTAAAAAATA

	TTAAATTGATATATCTCAATAAAAATATAATATATATATATATAAACTTTAAATTTTTAAAATGATTATATGA<br>TTTGAAAAATCAAATACAATATATGTGTTTATTATATATATATATAATATGAAGGTTGCGATAATTATATAAT<br>TTAAAATTATTTTTATTATTTAAAAATATTTATTTCAATATAATATATATAAAAATATAAATAAAAAATAAAA<br>TATTAAATTTATTTTTAAAATTAATTTTAAACATTTAAAAATGATATATTTTATGAAAAATATAGGTGTTTAA<br>TATATACGGGTTTAGTTTTGGATTGTTAGTGTAAAGTAGACATTTTTTTTATCATGAACCAATTAAAATATTT<br>GGCAGAGACAATTTTAAAATAAATATTATTAAAGTTAATAATTTTTAATTAGATAAAATTATAAAAAATTATT<br>TTGTTAATTAAATTGTTATTATGTAAAAAAAATATAATAATATTTTTTAGTAAAATTTATTATTTTAAAATAT<br>TTAAAATCAATCTCAAAAGCTAAGAACGAAGCAGATTAACAAAAAATGCAGACATCTTTAATTAATTAATTAA<br>TATTTTTCTATATTTGTATTGTACAGTCGTAGTCCACTTTGTTCCAGGTGATCGTACATGCTTTTCAGCATAT<br>TTTCTGGATCAAAATACAAAACCAATCAAACGATGCCACGTGTAAACCGCACTTGAAGTATCCTACAAAGTCA<br>CACACTAGTGACTTCCTTAGAAGGTAACAA
CrMTP9.1-Pro	TTTAAAACGGAAAAGAATTATTAAATATTATTCTTAACTTTAAAAGATTTTTATTCTTAAATATATATGATCT<br>TATCTCTGTTAAACCATAATCTAATAATAATAATATAAATATATTTTTATTATTTTAAAAAATTAATTTTAAT<br>TTATTTATTTTTATATAATAAAAATAAAATAAATTCTTATTTAATATATAAATTTTATAAAAATCATAATATT<br>ATAATATGAATTTATACTATATAATCATTTATAATATAATATTATTATATATATTATTTTTATATCAAAATAA<br>ATATTAAATTATATAAAAATATTATAATAATATATAAATTAATTATTTTAAATTTTAATAATTAAATAATATA<br>TAAAATAAAAATTTATATAATTATATAATATTTTTATATTATAATCAGTATATATTATTATTATTTCATAAAT<br>ATTGTTTTATTTTATAATTATTAATATTATTTTTAGTATTATAATAATAATAAATTTTATTTCAAATTTTTTT<br>ATTATTATAAAAAATTAATTAACTATCAATTATTTATTTATTTTTTATAAATTTATAAATAAATAACATTAGT<br>TAATATATAAAAATTATATTTAATTTGTAATATTATAATATAATTTTATATTATGTAAATTTTTATAATATAA<br>TATATATTATTTTTATATAAAATAAATATTAATAATATAAAAATTATAATAATAATAATGTATAAAATTAATT<br>ATTTTATATTAATTATTAAATAATATATAAAATATAAAATTATATAATTATATATCATTATTATATTATAACA<br>ATCAATATAACTATCATAATAATAATAATAATAATAATAATATAATTATTATTTAAAAGAATTTAATTTTTTA<br>TTTATTTAATTTATATATATTAAAAATAGATAATATTAATTTTTATATAAATTATATTAAATTTATAATATTA<br>TGATATTTTTATATTATATAACTATTTATTAAATAATATTATAATATATATTATTTTTATATAAAATAAATAT<br>TTAATAATATAAAAATATCATAAAAATAATATATATAAATTTTTTATCTTAACAGTAATAATTAAATAATATA<br>TAAAATATAATTTTATATAATTATATAATATTTTTTATATTATAACAATTATATTAAAAATCAGAATAACTTA<br>ATAGAAAGATTTATAGATAAAATTTTAATTCAATATATAATTATAACTTCAAAATTACTTAAATTTTTAGAAA<br>ATAATAAATAATAAATATATGATATTTATATTTTAATATATTTTACTTTTTCTATATTTTTTTTTCTATTATA<br>TCTCGTCTCTTTTTTATTTTTTATCTTTTATATTTTTTTCTTTCATCTTAACATATCTTTTTTTACTTTTTTT<br>CCTTTCATCCTATCTTTTATAATATTTTTATTCTGATATTATATATATATATATATATATATATATATTTATC<br>ATATTTTATCTTTTATTGTTTAATACATTTGAAATAGAATATATAAAAAAATATTTTTCTCATAAAACTAACC<br>AATTAACAATATACTAAAAATTATATTGATCATAATTCTAAAAGATAATAAATGTATATTTTCATATTATTAA<br>TATTTTCATATATTCATTTTTTTTTATTTCTATTCTTTTATATTCTAATACACGAATGAATGGAGTATAAAAAT<br>ATCATGCACAATAATTCTCCCGAACTAGTGAAACAAACATCAAGCAAGCACAGCATCGTACAATCCACAATTT<br>TTTTTTCATTGCAGAAGCAACCCACAATTCTCGTGATTCTTGTTACAAGGATTCAAATTTATATGTGTATGAA<br>AACAAAAATAATAAAAAACAGTAGTTCTGGTTACAAGGATTCATATATATATTAAAGTAAAAATAATAAAACA<br>CAGGGTCCACATGTTGCCACGTGTGGACCACAATTTCACTATCGTGAGGAAGATACACACAGTGAAGTTTCCT<br>CTTCCGTGTATAGGTCCTATCATTAGGGAG
CrMTP10-Pro	TTTTGAAAAAATATTTGTAAAAGTTGGGTTCAAATATAATGGTGGGAACGTCACGTTACTAATAAAATAAATA<br>AAAATACATTAGAAACTAACAAGTAAATATATTAATTTTAAATATTAAATTAATTTTAAAACTTTAAAAAGGT<br>TAGAATTAGAATTAAATCTTAATAAGAATAAATTTTTTAAATCTTCTTTACCTTTCATTAAAGATATTTATTT<br>AAAGAAAAAAATAAAAAAGATATTTATTTTAATTTTTTATTTTAAATTTTAGATAAATCAATAAATACTACATT

CrMTP11-Pro	ATATACATATTTAACTTTTTTTTATGTTATAAAAAATTTAGAGAAGATGAATTTATTTTTTTTACTTATTAGG<br>GTAATATATATTTTAAGATTCAGAAAATACTTACAAATCATCTACCATTAAGATTCTCTTATGATCTTATAAA<br>TGAAGAATCTTATTCTTATCAACTTGATCAATGGTGGATTGAATCAAAACAATAATGATTGAATCTCATTAGA<br>TTATGATAAGAATTGTGAAAGAAAGATTACTTTGTTACTTATAATATCTCATTGCATATTTAATTGAGTGAAA<br>GATATTATTTTACTTTACAAAACATACAATATTTTTACTTCCTAGAGATATTTATAGATGGTCATTTTTTCTT<br>CAAAATGAAAGTTAATAAAAAAGTACAATCTTAAGTAATAGAGATAAAAAATAATAGTTATTAAAAAAGTTAA<br>AGACCTTATCAAGTAGAAGTTATAGATCTCCTAGGATCTCTGGAGTGCATGAAGTTGAGTTGAATGATTCTAA<br>TTCTAAGAGGATAAATAAAACCATGACCATAAAGTCAAAGAAATAATAGAAAAATCAATCACCTCTTTAAAGG<br>CTCTCAAGGGAGAAGAGTTATTAGAAAGTTCAAAAGAATTAAAAAAGAAAAATTTAAGACTAATGATGACCTA<br>TCTATTATCATCACTTCACTTGACAAAAAAAATATTTAGAGAACATTTGTGAGGTGAGGTGTAGGATTTTGTA<br>TAGAGAAAGTTATAAAGTATACATTAAGAATACTCTTTTATGAATGGTTAGGGTTTTAAATTTAAAAAAAAAA<br>TGATAAGGTAAAGTGTAGAATTGTGTATAGGAAAGGGTGTTCATGGACAATATTTGGGAGTAAAGTTGGAGGA<br>CCATTGATAAATAGGGTAAGATGAAATATTTTAAGTGGTTGCAAATACTGGCTTTTCAAAACAAATATTTGGT<br>ATAATTTTATCTCATGAACAAGAATATGCCAAGCAATTACCAACTAAATCAACAGTACCTTAAAACAATTATC<br>AATTAAACCAAAGATACCCTTCCATGCTGCTGCCATATGCCAGAACAGTGATGCCTCCATCAACACTGTTAAT<br>TACCTAACAAAGACCACTTTATATGCAACCATTTCAATTTTTTTAATTAACAAGTATGAACACAGGATTCGTT<br>TTATGTTATTATTATGTATATGATCATTTCATTTTAGGAAATTTAAAAAAAAAAAAAAACTGAATGGAGAATC<br>CTTATTAGAAACGATATAAAGTAATTCCAATAACTCATTGTTGAGTTTGGGTTATTTCCTGGTAACTTGGACA<br>ATGGACTAGTTGTGCTAAAAACAATAAAAAATTGTAAGTTTTTTAATTTAAGTAAAAGTTGAATGTTCTAATC<br>CAAAAATAAAAAATTGAAGCTTGAAATGTGAACACTGAAACACTAAACAAATATTCCTAAGGTGGATTCCGTT<br>CATATTAACATAGACTACTTAAATTCACAAAGTCAATTTTTTTATAGACATTATATAATAGAAAGACAAATAT<br>ATGGTTCATGCCAGAATTAGTAATCAAGCATTGTCCATGCTTTCTATAGTTAAATTATTCTTCCCAAGTTTAC<br>CAAGTTATAATATTCCATATTAACTACTACAGTAACCACTTGCTTTATGGTGCTTTAATTACTATATATATAC<br>ATAAGGTACAAACAAATGCTTAGAATTGAA<br>ATTTTTATATTATTTTAATTTTCTTTATATACAATTTCATACAAAATTTATGAAAGATTAGTAAAAAATTTGT<br>GTAATTTAAAAATATTTGATATTATATTTTTATATAAATTTTAAAATATAAAATTATTGTATCAATTTTATTA<br>AAATTTTAAAAATACAAATATAATTTATCCTAAAATTTATGTTAATACTATTTTATAAAATTGAAAATTTCAT<br>ATACAATTATTTTACAATATTTTAGAATAAAAACTGATGTAGAATACCTGCTCATTTTATTTTAAATAATTAC<br>ACTAATTTTTAAAATATAAGTTTGAATTTTTTTTAGTTGAAATAGATAGATCAAATATATTTTTAATTATTTT<br>AAAATAAATTAATTTTTAATTCTTATTATTAGATATATAATAGTATCAAGTATAAATTATATAAAACATTAAA<br>ATTAATTTATTTTAAATATAAAATTAAAAATTAAAAATATATTTACCCCACCAGATTTGAAGTTTGAATAAGG<br>CGATACCAGACGGTATTAATGATGGGATGGGGATGTCATCCATATCATACAGTGGGCCCCAGAATAACAGAAT<br>CCCACTCACCACACCACATAATGCCCCTCCATTATTACGGTCACATATCGCATAACAGTATGTATTTTTTGAA<br>TATTCTCATCCATTAAAGTCCCTATTCAGCTATTGTCAGAAAATTGATTTCATGCAATAAAATGAGAATTATA<br>TAACTTTTTATCATTTTAAATTCATATATATGCACCTTAATATTCCATATTTAGTTATTTATTTATATAAAAT<br>TATTCTCCTTCTATACTATAAATTTCTCTTACTTTTGATATACAGTGTATTTATAGTAAAATATAATTAAAAA<br>GTAAAGAAAATAATTAATTAAATTATTTGTAAGATAATCTTAACTTTATTTAGCATGATGATTATTTTAATAA<br>TTAAACGAAATAATTAAAAATTTTAATATAATAATGTTTAAAATTTTAATTATTAGTTAATTTTTTTAATATA<br>TTTAATATTTTTTATTTATTATAAGATATTAATTTTATATTATTATTTAATATTATAATATTTAAATTAGTTA<br>TTAAATATTTTATTTAATTATTTATAATAAACATTTAATTTTTTATAAAAATTTAAAAATGAATAAAAACTTT<br>TCATAGAAATATTTTTAAATTTCAATTATGATAATTAAAAATAAATATATTTAAATTTTAAAATAATTTAAAA<br>GTATAAATTTTATTAAAATTATATCATAAATATAAAATAAATAAATAAAATTATCAAATTTTTTTAGTATATA<br>ATCATCAAAATCATTATAAATTTATTATATAATTTTTATAATAGAAATATATAAAAAGAAATAAATATAAAAA

	TATTATCCTTATTTTTAATATAATAATTTAATTTAAATAATATAAACTTAAAATTAATCATTTAAATTATAAT<br>ATTTAAAATTTTATTTATTATTTTATCTTTATTAACTAATTTAAAAATTAAATTTATTAAATATTTTTTATTT<br>TAATAAAAAAATTTCAGCTCACAAATTTTAAATAATTTTTCAATTTTAAATTTTAAATTAACATATATGATAA<br>TTTACTAAACACATCCATAATAAAAACATTAATTTTATAATTTATTATCATATAATAATTGTTTATTTTAAAG<br>CCACTCCAACACAAAACTCAATGCCTAAACCGGCTTTAAAGCCACCGGGACCCCACTGGCCACTGGTCCTGAT<br>TCATGTGGCTCTTGGATAGTAGCCACATCAAACCAATCTTTCGAACACTGAATCTCAGCCGTCCACGTGTTAA<br>TAACTCAAATTCTTAACAGACGCTCGATTAATCCACGCTCCAACACCAACCAACAAAACCGGAACACGTATCA<br>ACATCTTCGGAAAGTCACGATCCCGTGCATCACACGCGCTATCAGATCTCAGTCTTTTCACTTTTCATCTACC<br>AACATTGAATCCCCATGTTCTCGGTTCAAA
CrMTP12-Pro	GAAATTTTTAATAAAAAAATATGCATTTTTCTAATTAATTTTTTTTAGGATTTTGATTTAAAATTTAATAAAT<br>AATTTTTTATTAAGTTTACAGTGGAAAATATTAAAAATTATGATAGCAGCAAATTAAAAACATTTTAATAATT<br>TTTTACTATTTTTACAATATGGGCATAAAAAAATTACTTATCTTTATTCATTTTCTGGATTACTTTTGATTAT<br>ATTTCTTACTCATGCAATTACATTTTAATGTCTAAACTTATTAACATTATACAAGATTGTAAAATTTATATGT<br>AAATGATTTATTTTAAATTACACAAAAAATGTTTGTATCATCATGGTTTGGTAATAAATACTGATATTTGCGT<br>TTGAAAATGATTTAAAATACTAACTTTTGATGATGAAAAAATATCTAAGTTCTTCTATTAGGAGAAATTTTGT<br>TTATAAAGAGTTTTTCTTAATGTTTGATTATTTTATAAGAATTTAAATGGAATGTATTAGTAATTTAAATATT<br>TTTATATTTTTATTTAATTATAAATTATCATATTTTATAAATTATTAATTTTATAATAATTATATTATCATAA<br>TTTTTAATTAGTTGACAATGTAAAATTTTTATATTAATAATATATCAAAAATTAAATTTAATTATTTTTAACT<br>TAAAAATTGTTGTTATGAAATATATTTATTATTTCCCCTTTTAATGGATCTTATAACGATAATATACATTTAT<br>TTACTTTCTTTAATTTTAATTTTAAATTTATTAGTATTTATTTTTATTCTAAGAAAAGTATTGCTATAAATTA<br>GTTTTCTTCTTTTTCCTTATAATGTTCTTATATAGTGTTAGTCATATTAACATTGCTTTCGTGTTAATTTTTC<br>ATATTAAAATTGTCTTATTACCATAATATTTATATTTTATTAAATAATTATTTTGACTCACATAAAATCTTGA<br>TTAAGATATTTTTCTATCAATATTATTTTATTTTCTTTATCAAAAATTTATTTACAGAAAATATATATTAATA<br>AACTAACAAGTTATCCATATGTTACAAAGATTTTTTTTATATTAAATAAAATTTATGAATAAAGATAAATTAA<br>AAAAAAGATACGTATCATTATTATTTAAGAATAAATGTTTTATTATATTAAACAAAAATGAATTAAAGATATA<br>AATTTTTTAAATTAAATAAAAAATTTTAAATTAATATTATCATCTTTTTTTATAGAATATCAAAATTTAAATT<br>AAGATTAACTAAATTATAACAATCTTAAAATTTAGTTTATATAAAAAGAAGATATTTTCAATTTGATATATTA<br>TTTCAAAATTTAAATTTTAAATAAATAAATAATTTTAAAAAAATAATTTCTACTATATTGATTAAAATTATCA<br>TTTTTAAAAAAGAAGTGATTAGTGAACTGCATTTTCTTAATAAATATATTTTCTATATTATCTCTCTTTCCTT<br>TTATAGAATGTGATTTTTTTTGAAGTTATAGAATACAATTTTCACACAAAATTTTTTTATTATTAAATTTAAA<br>TATTTAACCATATATTAATATATTTATTTAAATTATACCGATTATTCTTTAATATTTATGTGCACATTAAATT<br>TGACTTTAAATGAATTATCTTCTACTAATAGTTATATAATTGGTATATAATAAAACAATAAGTTTAATATAAT<br>TATTAATAATAAAAAATACTATTTTATTTAATTTCTTCTTTAAAAAATACTATTTAGTTAACCTAGCTTCAGC<br>TAACCTATCTAATCCTAGCTTCGCCTAACCTAGCTAATTAAGCCACTATTGAAGTTAACCTAGCTTCGCCTAA<br>CCTAGCTAATTAAGCCACTATTGAAGCTGCCGTACCTCATTGATCGTGGCTTGAAAGTTCACTCGATCTTTTA<br>TTTCGTTTCCAAATGCAAAATCACTTAACCGAGCAGGTGTTGATATTTCCCTTTTTCTCCCCGTAGGGAACTG<br>TAGACCCATAGTCCACCCTTCTCCACTGCG

Table S2. Primer sequences used in this study.

Primer ID	Sequence (from 5′ to 3′)	Usage or purpose
CrMTP1F	TACCGAGCTCGGATCCATGGTTTTTCTCCAACTCAAAA TG	Primer pair for construction of CrMTP1-pYES2 for functional verification in yeast
CrMTP1R	GATATCTGCAGAATTCCTAGCGCTCTATCTGTATAG	
CrMTP4F	TACCGAGCTCGGATCCATGAAGCAAATGGAACATGAG	Primer pair for construction of CrMTP4-pYES2
CrMTP4R	GATATCTGCAGAATTCTTACTCAATTTGTATTGTTAC	for functional verification in yeast
CrMTP5F	TACCGAGCTCGGATCCATGGAAGCCAGGGAGACTCC	Primer pair for construction of CrMTP5-pYES2
CrMTP5R	GATATCTGCAGAATTCTCATGCATCGTCGGTCTGCAC	for functional verification in yeast

CrMTP8.1F<br>CrMTP8.1R	TACCGAGCTCGGATCCATGGACAGGAATTCGGGTTCG<br>GATATCTGCAGAATTCCTAATTGTTGGGCAGTTTGC	Primer pair for construction of CrMTP8.1-pYES2 for functional verification in yeast
CrMTP10F<br>CrMTP10R	TACCGAGCTCGGATCCATGGGCACTGAACTTAGAAC<br>GATATCTGCAGAATTCTTAATTTGACTTGTGCTCCAGC	Primer pair for construction of CrMTP10-pYES2 for functional verification in yeast
CrMTP11F<br>CrMTP11R	TACCGAGCTCGGATCCATGTTGGAGCCGGTGGAGCTTC<br>GATATCTGCAGAATTCCTAGGAGTGTGCTTGAGCATG	Primer pair for construction of CrMTP11-pYES2 for functional verification in yeast
CrMTP12F<br>CrMTP12R	TACCGAGCTCGGATCCATGGCCGATCAGCCTCACCAC<br>GATATCTGCAGAATTCTTATCCAACAGATTCCACTTG	Primer pair for construction of CrMTP12-pYES2 for functional verification in yeast
CrEF-αRTF<br>CrEF-αRTR	GACCTTCTTCGTTTCTCGCA<br>CGAACCTCTCAATCACACGC	Primer pair for qRT-PCR of reference gene *CrEF-α* in *C. rosea*
CrMTP1RTF<br>CrMTP1RTR	AGGCTAACAGTCTTGCGATATT<br>CCATCCAGCAGCCCATAAA	Primer pair for qRT-PCR of *CrMTP1* in *C. rosea*
CrMTP4RTF<br>CrMTP4RTR	GCTGAACCGGAGTGGTTTATAG<br>TGGTGTCCTTTCCATCAGAATG	Primer pair for qRT-PCR of *CrMTP4* in *C. rosea*
CrMTP5RTF<br>CrMTP5RTR	TGGCTGCATCTAGGAAGAAAG<br>CCTCAACTGCAAGGGAGAAT	Primer pair for qRT-PCR of *CrMTP5* in *C. rosea*
CrMTP6RTF<br>CrMTP6RTR	GCATCCTACCGTGACTGAAAT<br>CATGTCTCGGCTCCAACTATC	Primer pair for qRT-PCR of *CrMTP6* in *C. rosea*
CrMTP7RTF<br>CrMTP7RTR	CATCATTGGTTGCTGTGAATGT<br>CATGTCGGTTCCTCTGGATAAG	Primer pair for qRT-PCR of *CrMTP7* in *C. rosea*
CrMTP8RTF<br>CrMTP8RTR	GCTGACTGCACTGATGTAGAA<br>CGCTATTGACCCACTCCTTATT	Primer pair for qRT-PCR of *CrMTP8* in *C. rosea*
CrMTP8.1RTF<br>CrMTP8.1RTR	GAACAGGCTCAACAAGAGAGAG<br>CCACTCCTAACCGTAGCATAGA	Primer pair for qRT-PCR of *CrMTP8.1* in *C. rosea*
CrMTP9RTF<br>CrMTP9RTR	GATTAGCTGCTGCTGTCCTT<br>ACTGTCTTCGCCCATGTATTT	Primer pair for qRT-PCR of *CrMTP9* in *C. rosea*
CrMTP9.1RTF<br>CrMTP9.1RTR	GTCTCACCGAGGATGAAATGAA<br>GCTCGCAATTGAGGCATAAAC	Primer pair for qRT-PCR of *CrMTP9.1* in *C. rosea*
CrMTP10RTF<br>CrMTP10RTR	GGTATCAAGAAGTGGACTCGTATC<br>CAGCACCATGTTCCCTATGT	Primer pair for qRT-PCR of *CrMTP10* in *C. rosea*
CrMTP11RTF<br>CrMTP11RTR	CTATCAGCAGCAGGTTGAAGT<br>TTTGTCTCGCTCCTCCTTTG	Primer pair for qRT-PCR of *CrMTP11* in *C. rosea*
CrMTP12RTF<br>CrMTP12RTR	GGGTTCGTGTCCTTGTTTCTA<br>GCCAAACCCTCACACATTTC	Primer pair for qRT-PCR of *CrMTP12* in *C. rosea*
